# Supplementary material for: Epigenomic priming of immune genes implicates oligodendroglia in multiple sclerosis susceptibility
Source: Neuron. Author manuscript; Available in PMC 2023 Apr 6. (PMC9810341; doi:10.1016/j.neuron.2021.12.034)
Supplement: Supplementary Material [file NIHMS1854707-supplement-Supplementary_Material.zip › 1-s2.0-S0896627321010898-mmc1.pdf]

Neuron, Volume *110*

Supplemental information

Epigenomic priming of immune genes implicates oligodendroglia in multiple sclerosis susceptibility

Mandy Meijer, Eneritz Agirre, Mukund Kabbe, Cassandra A. van Tuijn, Abeer Heskol, Chao Zheng, Ana Mendanha Falcão, Marek Bartosovic, Leslie Kirby, Daniela Calini, Michael R. Johnson, M. Ryan Corces, Thomas J. Montine, Xingqi Chen, Howard Y. Chang, Dheeraj Malhotra, and Gonçalo Castelo-Branco

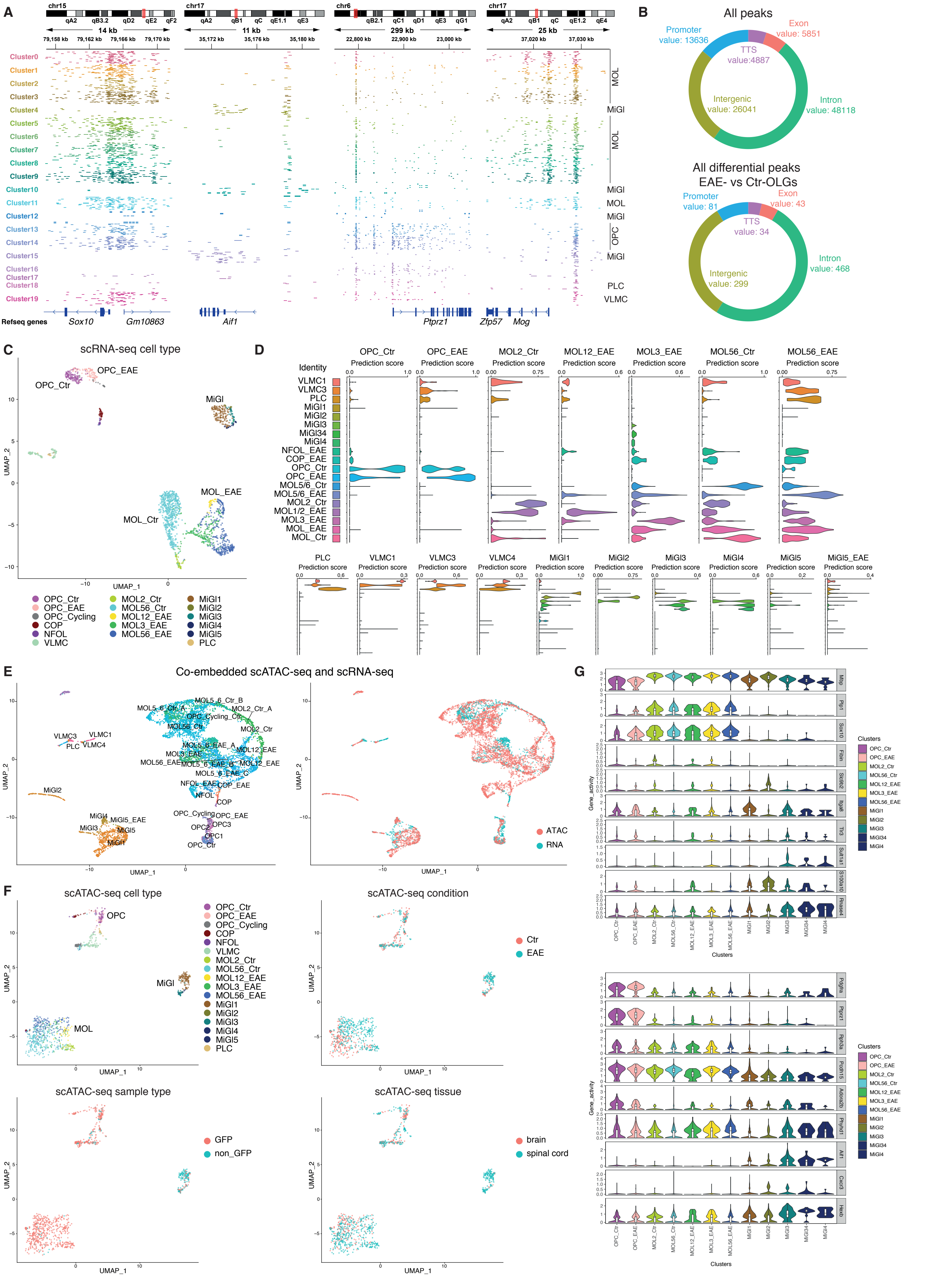

Figure S1. Single-cell ATAC-Seq in the EAE mouse model of MS; integration with single-cell RNA-Seq, related to Figure 1

A, IGV tracks of chromatin accessibility in 50 randomly selected individual cells for each of 20 cell clusters identified with unsupervised clustering. Colors represent the cluster as in Fig. 1b. Marker genes are shown for OLG (*Sox10*), MiGl (*Aif1*), OPCs (*Ptprz1*) and MOLs (*Mog*). Genomic coordinates are shown. **B,** Distribution of accessibility of all peaks in all cells (All peaks) or differential accessibility peaks between EAE- and Ctr-OLG. TTS = Transcription termination site. **C,** UMAP clustering of scRNA-seq data (Falcao *et al.*, 2019), showing the major clusters, colors represent the clusters as in Fig. 1d. **D,** Prediction scores for each population to show the performance of the scRNAseq integration with scATAC-seq data to predict cell-types. **E,** Co-embedding of scATAC-seq data with scRNA-seq data (Falcao *et al.*, 2019). Colors represent the clusters (left) or method (right). **F**, Plate-based scATAC-seq (PI-ATAC) on GFP+ and GFP- cells (Sox10:Cre-RCE:LoxP(EGFP)) collected from brains and spinal cords from EAE and Ctr mice. Clustering performed with UMAP based on differential accessibility. Shown are cell-types (label transfer from scRNA-seq (Falcao *et al.*, 2019)), condition (EAE vs. Ctr), sample type (GFP vs. non-GFP) and tissue (brain vs. spinal cord).**G,** Violin plots depicting enriched chromatin accessibility at the promoter regions of marker genes identified in scRNA-seq data (Falcao *et al.*, 2019) in the identified scATAC-seq populations.

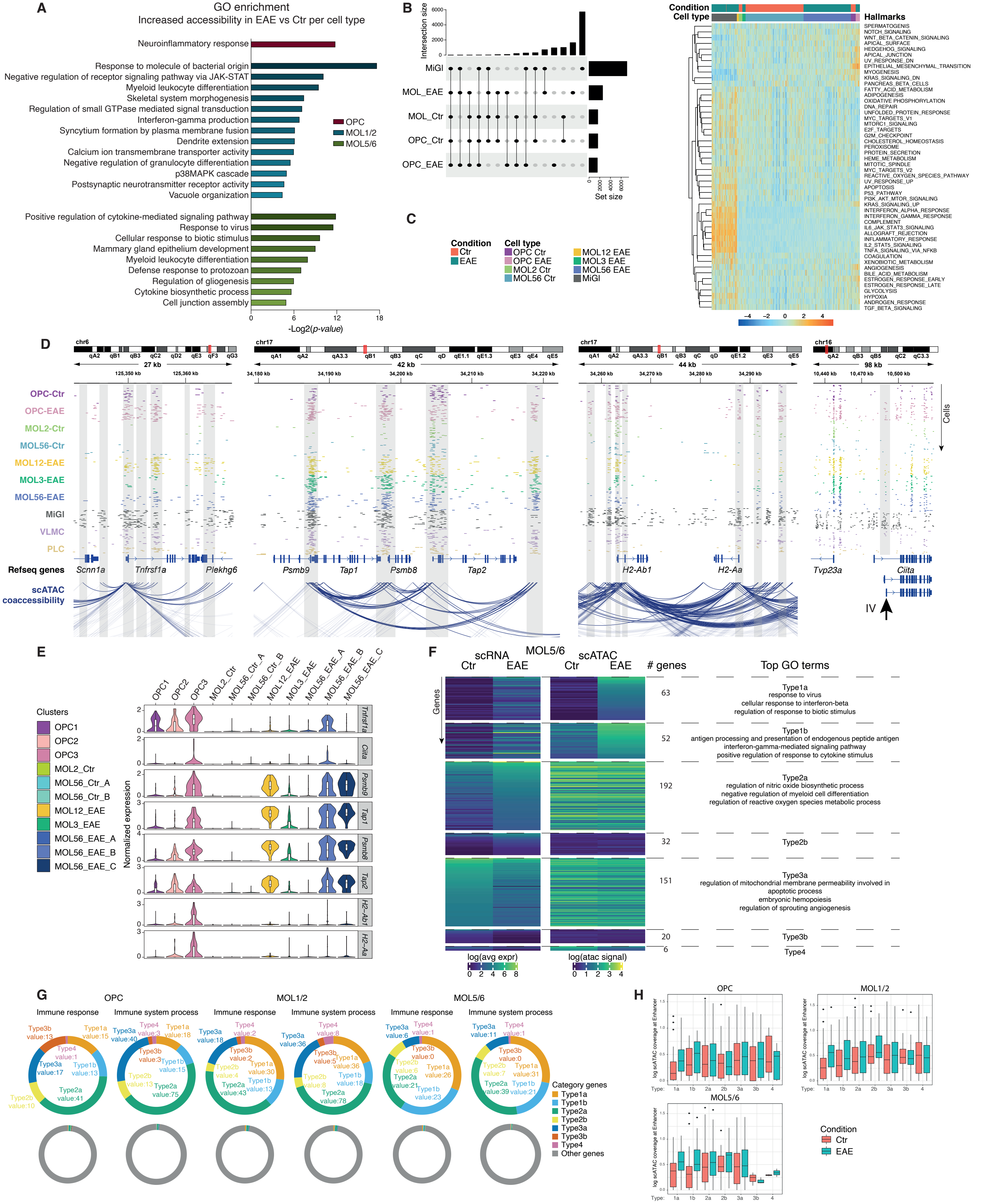

Figure S2. Single-cell ATAC-Seq in EAE mouse model of MS; Gene Ontology, hallmarks and patterns of coverage at promoters and enhancers, related to Figures 1 and 2. **A**, Gene Ontology enrichment analysis (biological processes) of the nearest genes to enriched accessibility peaks in EAE are shown for OPC, MOL1/2 and MOL5/6 populations. **B**, Upset plot with proportions of the promoter peaks with chromatin accessibility in microglia that also presented chromatin accessibility OLG clusters. **C**, Gene set enrichment analysis (GSEA) of gene loci have open chromatin in OPCs/OLs and MiGl. **D**, Integrative Genomics Viewer (IGV) tracks of chromatin accessibility in 50 randomly selected individual cells for each selected cluster, with MiGl clusters grouped together. scATAC accessibility connections are shown. Highlighted with grey boxes are regions with differential accessibility in specific clusters or promoter priming and connections between regulatory regions. Genomic coordinates are shown. Highlighted with a grey box and arrow is *Ciita* promoter region IV. **E**, Violin plots showing normalized RNA expression values per OLG cluster for selected genes (data from Falcao *et al.*, 2019). **F**, Genes in MOL5/6 are clustered based on gene expression differences between EAE vs. Ctr and correlation with chromatin accessibility activity score (accessibility over 500 bp promoter region). Top GO terms are shown for Type1-4. **G**, Distribution of immune genes found in GO terms as 'immune response' (GO:0002250) and 'immune system process' (GO:0002376) within the different RNA/chromatin accessibility subtypes define in Figure 2. Only a minority of genes in these immune GO categories are expressed or present open chromatin in OLG (bottom row). **H**, Normalized aggregated scATAC coverage over the putative enhancers (as predicted by ArchR) of Type 1-4 genes (from Figure 2a and S2F), in OPCs, MOL1/2 and MOL5/6 from Ctr and EAE conditions.

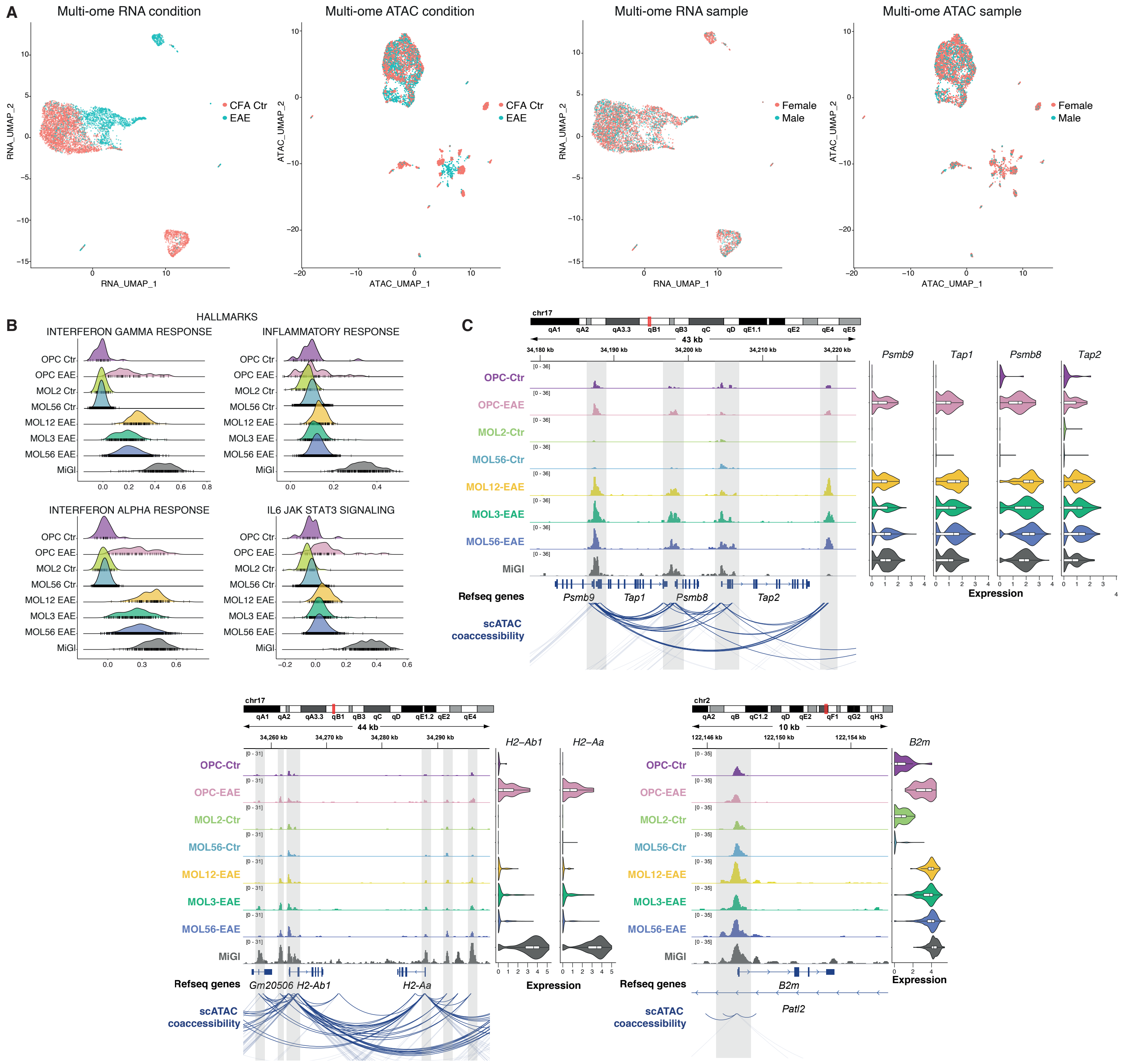

Fig S3. 10x Genomics multi-ome (simultaneous single cell chromatin accessibility and transcriptomics) of Sox10-GFP cells sorted from the spinal cord of Ctr (2) and EAE mice, related to Figure 2.

A, UMAP based on chromatin accessibility and transcriptome. Shown are condition (EAE vs. Ctr), sample type (GFP vs. non-GFP) and sex.

B, Gene set enrichment analysis (GSEA) of the nearest genes to enriched accessibility peaks for immune Hallmarks categories.

C, Left - Integrative Genomics Viewer (IGV) tracks of chromatin accessibility for each selected cluster, with MiGl clusters grouped together. scATAC co-accessibility connections are shown. Highlighted with grey boxes are regions with differential accessibility in specific clusters or promoter priming and connections between regulatory regions. Genomic coordinates are shown. Right – Violin plots depicting the expression of selected genes in each individual cluster.

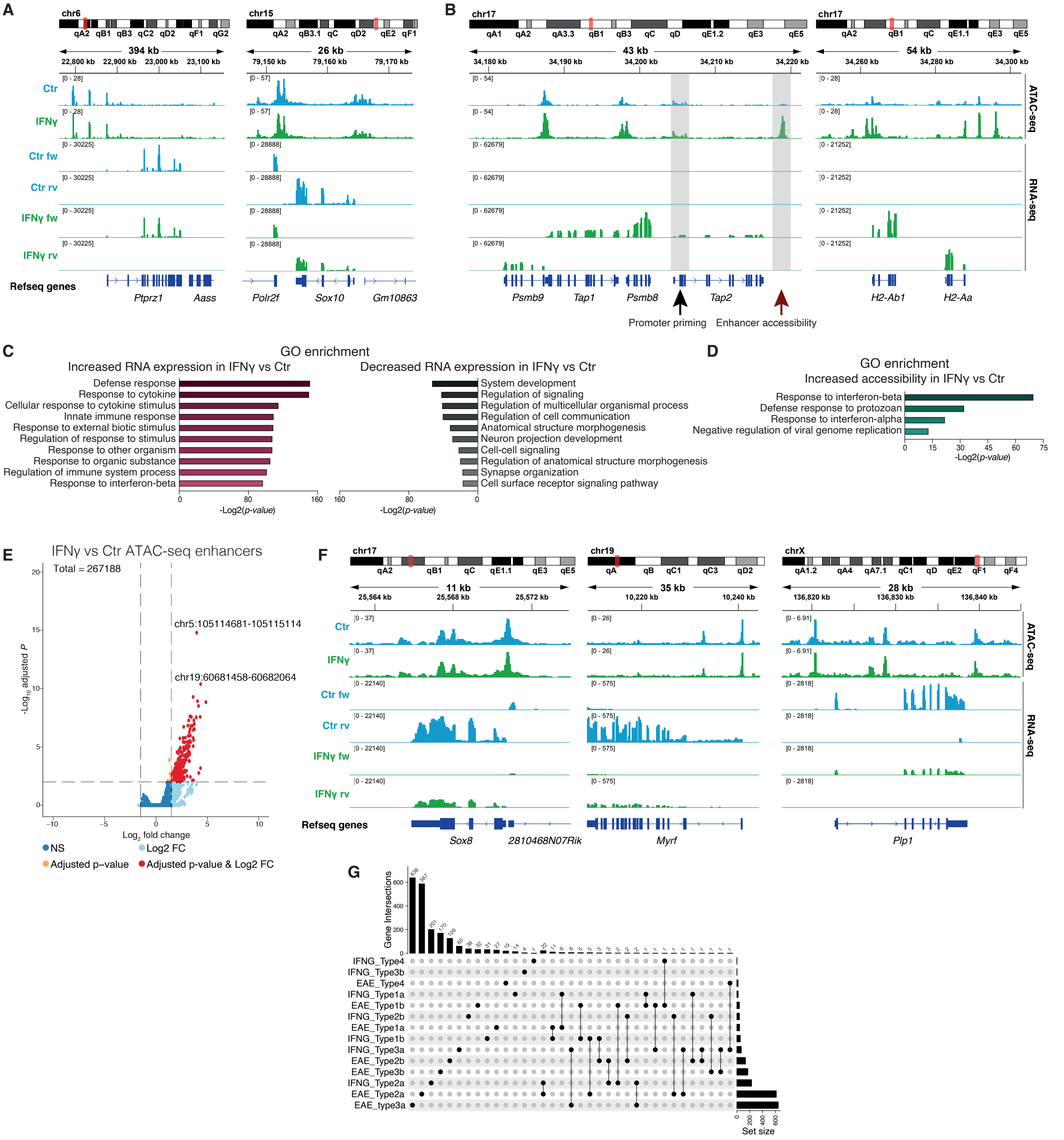

Figure S4. Chromatin landscape upon treatment of mouse primary OPCs with IFNγ, related to Figure 3

A, B, IGV tracks of RNA-seq and ATAC-seq from Ctr-OPCs and OPCs treated with 100 ng/ml IFNγ for 48 hours, centered at loci for marker genes of OPCs (*Ptprz1*) and OLG (*Sox10*) **(a)** and MHC-I pathway and MHC-II genes **(b)**. Highlighted with grey boxes and arrows are regions with differential accessibility in specific conditions or promoter priming, black arrows indicate promoter regions and red arrows indicate putative enhancers. Merged tracks of 3 replicates are shown for ATAC-seq and 4 replicates for RNA-seq. **C,** Top 10 Gene Ontology biological terms for genes upregulated upon IFNγ treatment in OPCs and downregulated upon IFNγ treatment. **D,** Gene Ontology biological terms for genes with increased chromatin accessibility at 500 bp promoter regions upon IFNγ treatment in OPCs. **E,** Volcano plot for chromatin accessibility at annotated enhancers between IFNγ-treated and Ctr-OPCs. Loci with statistical significance are shown in orange and genes with statistical significance and log2 fold change above 1.5 are shown in red. **F,** IGV tracks for genes with decreased expression in IFNγ treatment. **G,** Upset plot with intersection between genes with open chromatin in OPCs in vivo in EAE and in OPCs upon IFNg treatment.

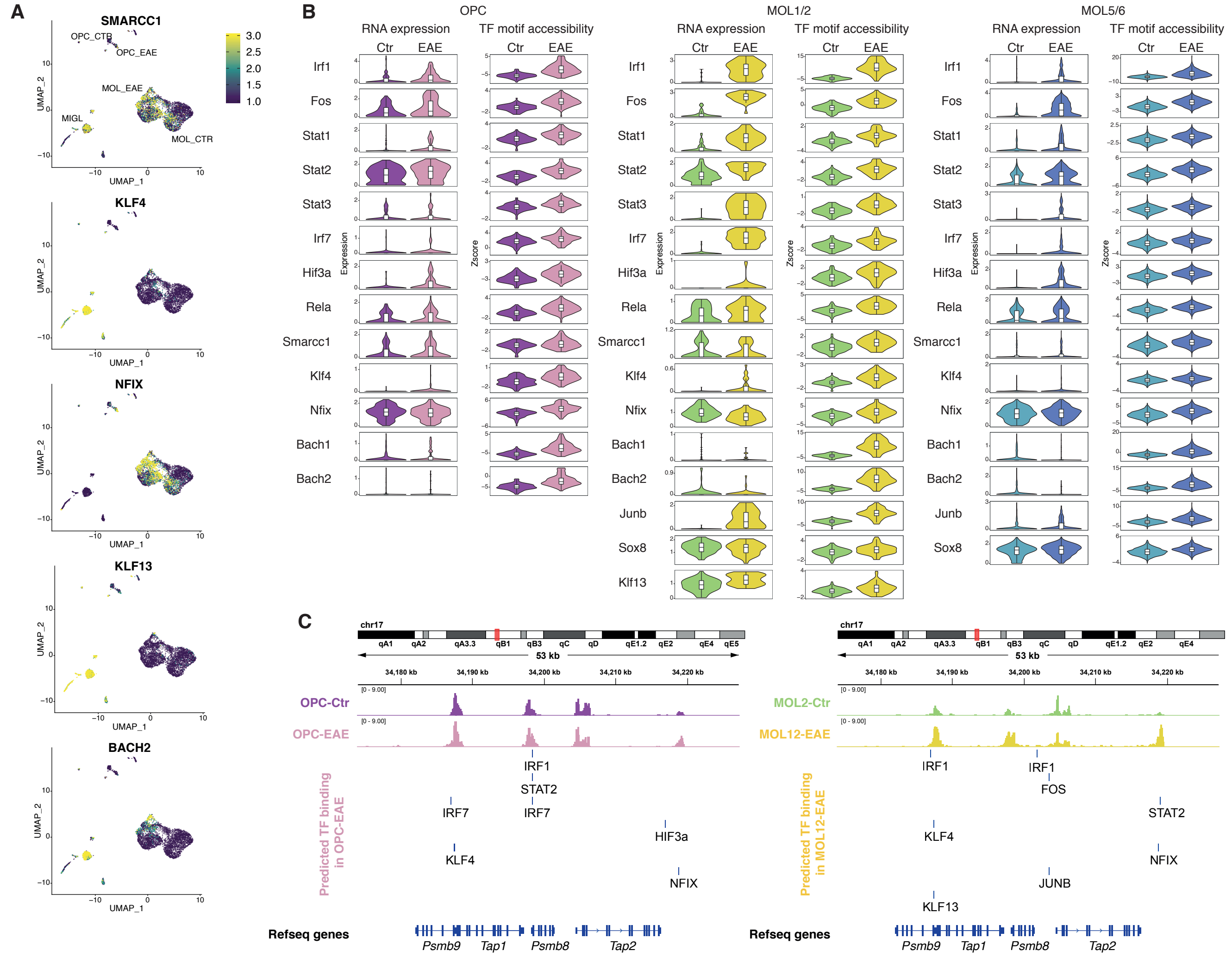

Figure S5. Transcription factor motif variability in oligodendroglia in the EAE mouse model of MS, related to Figure 4.
A, TF motif variability projected on top of UMAP clustering. **B,** Violin plots showing RNA expression values of selected TFs and their TF motif accessibility score in OPCs, MOL1/2 and MOL5/6, comparing cells derived from CFA-Ctr and EAE. **C,** IGV tracks of merged single cell chromatin accessibility of different populations and TF predicted binding sites in OPCs and MOL1/2 cells derived from EAE mice in the *Psmb9*-*Tap2* locus.

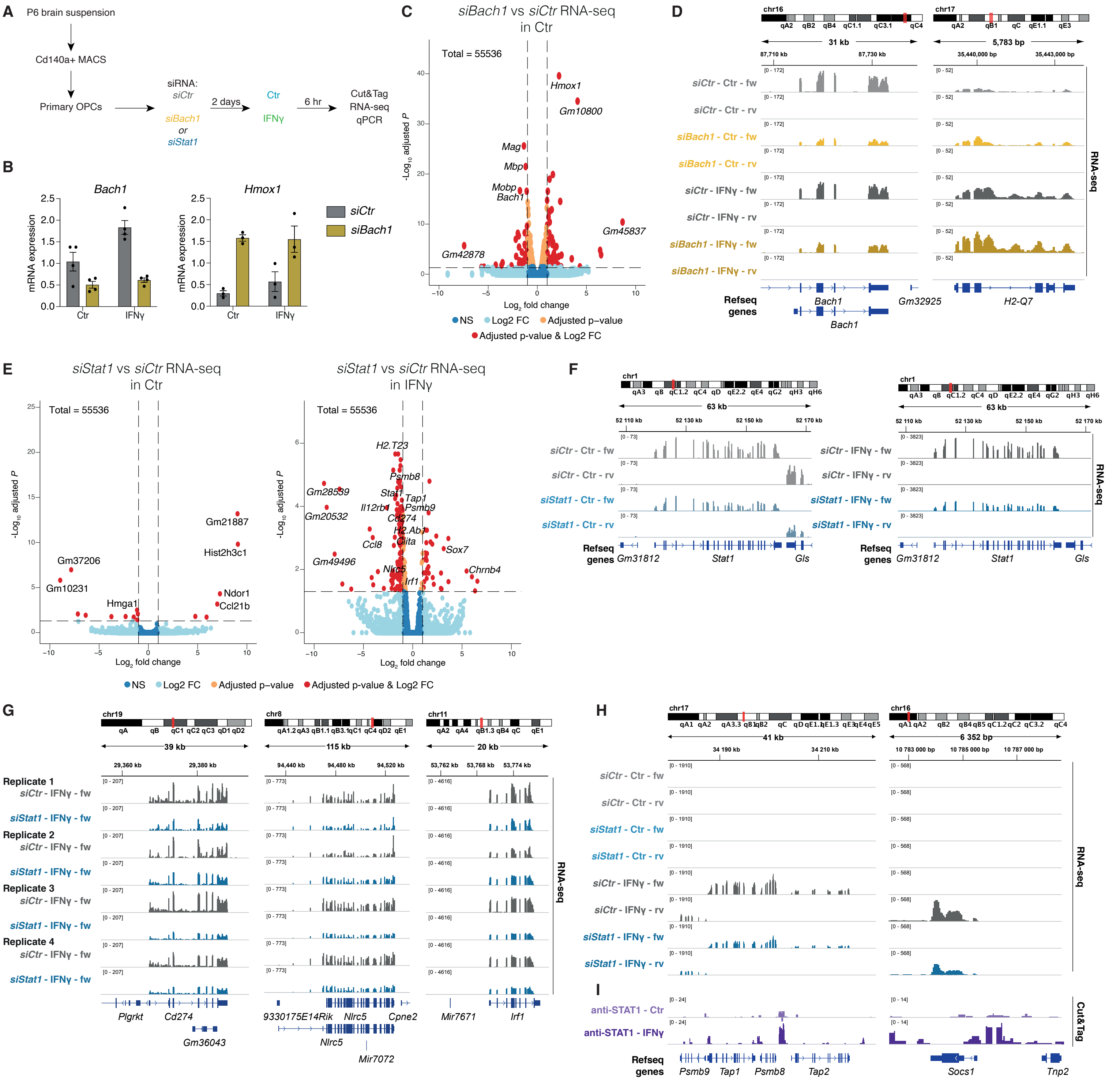

Figure S6. BACH1 and STAT1 transcription factor knock down in primary OPCs upon IFNγ, related to Figure 4

A, Schematic illustration of the experimental set-up of transcription factor knock down experiments. **B**, qRT-PCR targeting selected genes upon transfection of primary OPCs with siRNAs targeting *Bach1*. **C,** Volcano plots showing differential expressed genes in RNA-seq upon transfection of primary OPCs with siRNAs targeting *Bach1*. 4 biological replicates were performed. Genes with adj p-value < 0.05 and log2 fold change above 1.5 are shown in red. **D**, IGV tracks for RNA-seq upon transfection of primary OPCs with siRNAs targeting *Bach1*. **E**, Volcano plots showing differential expressed genes in RNA-seq upon transfection of primary OPCs with siRNAs targeting *Stat1* before treating with IFNγ for 6 hours. 4 biological replicates were performed. Genes with adj p-value < 0.05 and log2 fold change above 1.5 are shown in red. **F**, **G**, **H**, IGV tracks for RNA-seq in IFNγ-treated and Ctr-OPCs after transfection with with siRNAs targeting *Stat1* for selected genes. **I**, IGV tracks showing STAT1 binding in OPCs upon IFNγ treatment and in Ctr-OPCs, assessed with CUT&Tag. 3 biological replicates were performed.

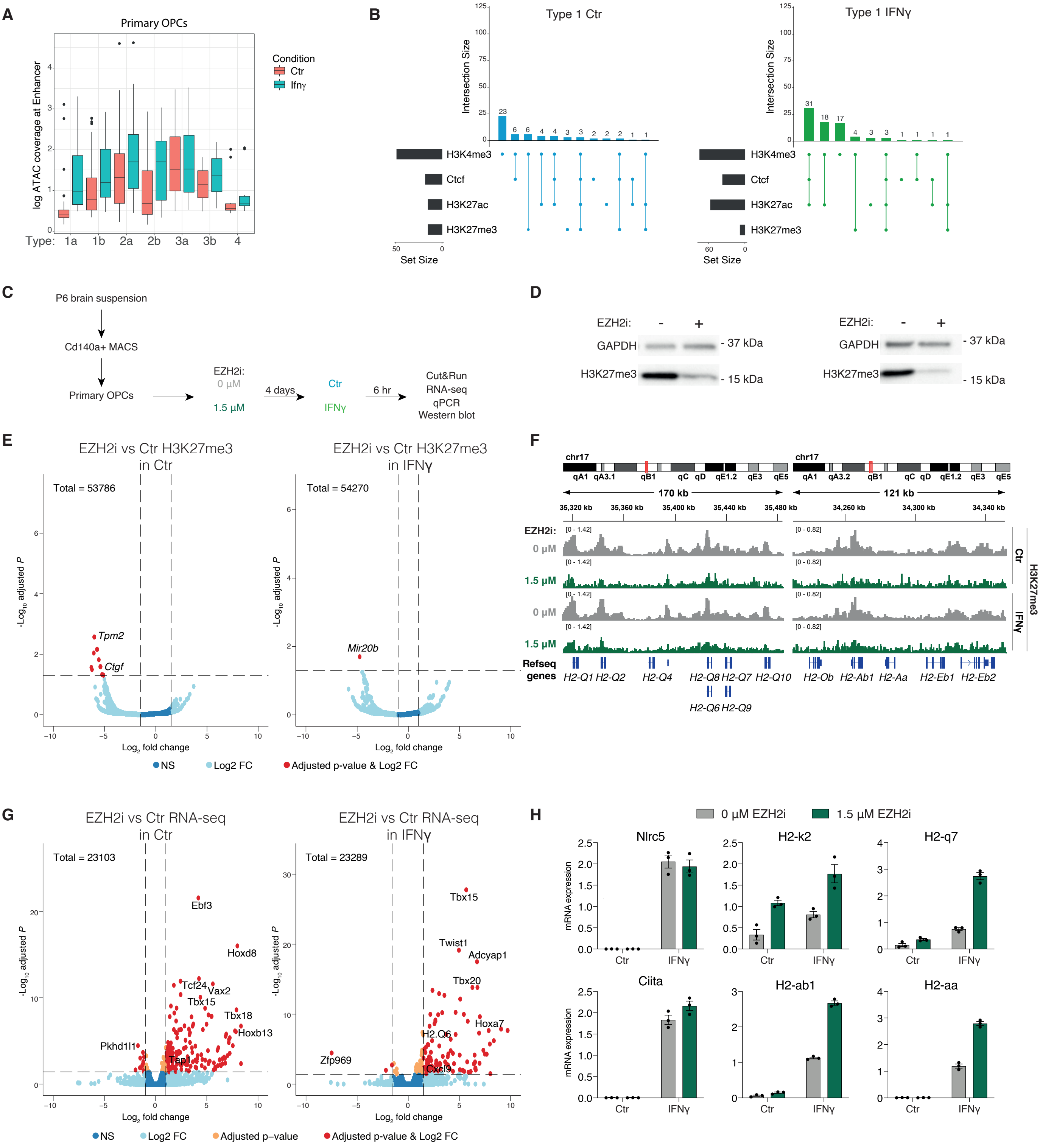

Figure S7, Chromatin accessibility and transcriptional changes upon treatment of mouse primary OPCs with the EZH2 inhibitor EPZ011989, related to Figure 3, 5 and 6.

A, ATAC coverage over enhancers (as predicted by ABC) of Type 1-4 genes (from Figure 3b) in OPCs treated with IFNγ. **B,** Upset plots of Cut&Run Ctr-OPCs and IFNγ treated peaks intersection in Type1 (genes with increased expression and chromatin accessibility with IFNγ). Top barplot shows the number of intersecting peaks per combination, left barplot shows the size of each peak dataset, the matrix shows the Cut&Run peaks sets (dots) and shared (connecting line) in each combination. **C,** Schematic overview of EZH2 inhibitor EPZ011989 (EZH2i) experiments. **D,** Western blot with antibodies against H3K27me3 and GAPDH (control) in EZH2i cells vs. Ctr in IFNγ-spiked OPCs, two replicates are shown. **E,** Volcano plots for differences in H3K27me3 occupancy between EZH2i vs. Ctr, in Ctr-OPCs (left) and OPCs upon IFNγ treatment (right), assessed with Cut&Run. Three replicates are performed. Genes with statistical significance and log2 fold change above 1.5 are shown in red. **F,** IGV tracks for H3K27me3 occupancy upon EZH2i vs. Ctr, in Ctr-OPCs and OPCs upon IFNγ treatment at MHC-I and MHC-II loci. Merged tracks of three replicates are shown. **G,** Volcano plots showing differential gene expression between EZH2i vs. Ctr, in Ctr-OPCs (left) and OPCs upon IFNγ treatment(right). Genes with statistical significance are shown in orange and genes with statistical significance and log2 fold change above 1 are shown in red. Three replicates are performed. **H,** qRT-PCR analysis of MHC-I and MHC-II pathway genes in OPCs upon treatment with 1,5 µM EZH2 inhibitor EPZ011989 (EZH2i) for 4 days, with subsequent co-treatment with 100 ng/ml IFNγ for 6 last hours. Error bars represent SEM, three replicates are shown.

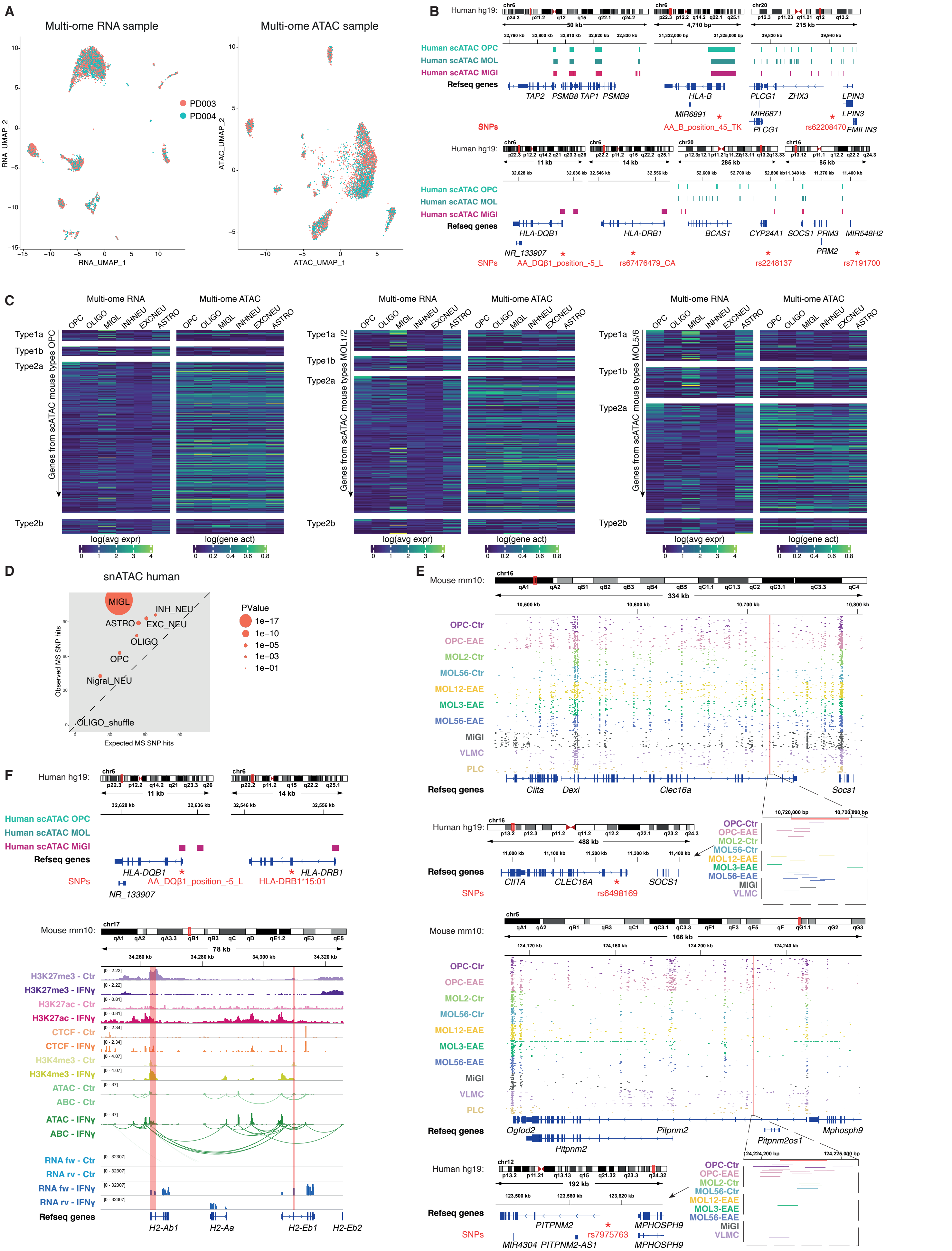

Figure S8, Chromatin accessibility at MS susceptibility loci in human and mouse oligodendroglia, related to Figures 2, 7 and 8. A, UMAP based on chromatin accessibility (right) and transcriptome (left) of 10x Genomics multi-ome of brain grey matter from 2 healthy individuals, by individual **B**, SNP coordinates for MS SNPs in the hg19 human genome reference and scATAC-seq data from human healthy individuals. **C**, Analysis of Type1 and 2 genes from mouse OLG (Fig. 2A) in the human multi-ome dataset. **D**, GREGOR overlap with MS associated GWAS variants. overlapping the human snATAC-seq peaks. Dot size scaled to adjusted p-value and adjusted p-values below 0.01 in red. **E,** SNP coordinates for two MS SNPs in the hg19 human genome reference and chromatin accessibility in scATAC-seq in corresponding locations in the mouse mm10 genome reference. IGV tracks of chromatin accessibility in 50 randomly selected individual cells from scATAC-seq are shown. Red boxes show scATAC-Seq peaks from mouse overlapping with SNP location. **F,** SNP coordinates for two MS SNPs in the hg19 human genome reference with corresponding chromatin accessibility regions derived from merged scATAC-seq populations from the adult brain from human healthy individuals. Corresponding locations in the mouse mm10 genome reference with IGV tracks for bulk ATAC-seq, RNA-seq, ABC model and Cut&Run against H3K27me3, H3K27ac, CTCF, H3K4me3 in IFNγ treated and Ctr-OPCs. Red boxes show chromatin accessibility peaks from mouse overlapping with SNP location and their ABC connections.
