# Supplementary material for: Epigenomic priming of immune genes implicates oligodendroglia in multiple sclerosis susceptibility
Source: Neuron. Author manuscript; Available in PMC 2023 Apr 6. (PMC9810341; doi:10.1016/j.neuron.2021.12.034)
Supplement: Supplementary Material [file NIHMS1854707-supplement-Supplementary_Material.zip › 1-s2.0-S0896627321010898-mmc10.pdf]

Neuron

Article

Epigenomic priming of immune genes implicates oligodendroglia in multiple sclerosis susceptibility

Graphical abstract

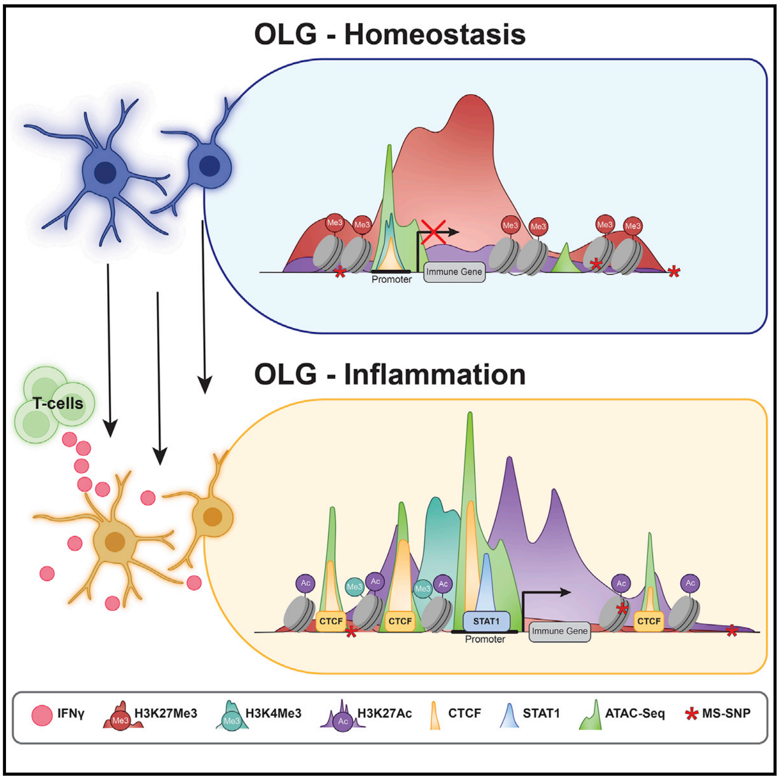

Highlights

- Chromatin priming of immune genes in OLG in homeostasis and disease
- BACH1, STAT1, and Polycomb involved in IFN-γ-mediated immune gene regulation in OPCs
- MS susceptibility SNPs overlap with open chromatin regions in OLG
- IFN-γ leads to altered chromatin and gene expression at SNP loci in mouse OPCs

Authors

Mandy Meijer, Eneritz Agirre, Mukund Kabbe, ..., Howard Y. Chang, Dheeraj Malhotra, Gonçalo Castelo-Branco

Correspondence

goncalo.castelo-branco@ki.se

In brief

Meijer, Agirre, et al. show that mouse and human oligodendroglia present open chromatin at immune genes and MS susceptibility single-nucleotide polymorphisms in homeostasis and disease. Transcriptional activation of these open chromatin regions involves transcription factors such as BACH1 and STAT1 and changes in histone modification deposition and chromatin architecture.

Meijer et al., 2022, Neuron *110*, 1193–1210
April 6, 2022 © 2021 The Author(s). Published by Elsevier Inc.
https://doi.org/10.1016/j.neuron.2021.12.034

CellPress

Neuron

CellPress
OPEN ACCESS

Article

Epigenomic priming of immune genes implicates oligodendroglia in multiple sclerosis susceptibility

Mandy Meijer,[1,13] Eneritz Agirre,[1,13] Mukund Kabbe,[1] Cassandra A. van Tuijn,[1] Abeer Heskol,[1,2] Chao Zheng,[1] Ana Mendanha Falcão,[1,3,4] Marek Bartosovic,[1] Leslie Kirby,[1] Daniela Calini,[5] Michael R. Johnson,[6] M. Ryan Corces,[7,8,9] Thomas J. Montine,[9] Xingqi Chen,[8,10] Howard Y. Chang,[8,11] Dheeraj Malhotra,[5] and Gonçalo Castelo-Branco[1,12,14,*]

[1]Laboratory of Molecular Neurobiology, Department of Medical Biochemistry and Biophysics, Karolinska Institutet, 171 77 Stockholm, Sweden

[2]Instituto Gulbenkian de Ciência, 2780-156 Oeiras, Portugal

[3]Life and Health Sciences Research Institute (ICVS), School of Medicine, University of Minho, Braga, Portugal

[4]ICVS/3B's Associate Laboratory, PT Government Associate Laboratory, 4710-057 Braga/Guimarães, Portugal

[5]Roche Pharma Research and Early Development, 4070 Basel, Switzerland

[6]Faculty of Medicine, Department of Brain Sciences, Imperial College of London, SW7 2AZ London, UK

[7]Gladstone Institute of Neurological Disease, San Francisco, CA 94158, USA

[8]Center for Personal Dynamic Regulomes and Howard Hughes Medical Institute, Stanford University, Stanford, CA, USA

[9]Department of Pathology, Stanford University School of Medicine, Stanford, CA, USA

[10]Department of Immunology, Genetics, and Pathology, Uppsala University, 751 85 Uppsala, Sweden

[11]Howard Hughes Medical Institute, Stanford University, Stanford, CA 94305-5101, USA

[12]Ming Wai Lau Centre for Reparative Medicine, Stockholm node, Karolinska Institutet, 171 77 Stockholm, Sweden

[13]These authors contributed equally

[14]Lead contact

*Correspondence: goncalo.castelo-branco@ki.se

https://doi.org/10.1016/j.neuron.2021.12.034

SUMMARY

Multiple sclerosis (MS) is characterized by a targeted attack on oligodendroglia (OLG) and myelin by immune cells, which are thought to be the main drivers of MS susceptibility. We found that immune genes exhibit a primed chromatin state in single mouse and human OLG in a non-disease context, compatible with transitions to immune-competent states in MS. We identified BACH1 and STAT1 as transcription factors involved in immune gene regulation in oligodendrocyte precursor cells (OPCs). A subset of immune genes presents bivalency of H3K4me3/H3K27me3 in OPCs, with Polycomb inhibition leading to their increased activation upon interferon gamma (IFN-γ) treatment. Some MS susceptibility single-nucleotide polymorphisms (SNPs) overlap with these regulatory regions in mouse and human OLG. Treatment of mouse OPCs with IFN-γ leads to chromatin architecture remodeling at these loci and altered expression of interacting genes. Thus, the susceptibility for MS may involve OLG, which therefore constitutes novel targets for immunological-based therapies for MS.

INTRODUCTION

Genome-wide association studies (GWASs) have allowed the identification of hundreds of single-nucleotide polymorphisms (SNPs) associated with susceptibility for multiple sclerosis (MS), many of which are located near genes expressed in the central and peripheral immune system (International Multiple Sclerosis Genetics Consortium, 2019). The etiology of MS is thought to involve malfunction of peripheral immune cells, which migrate to the central nervous system (CNS) and target oligodendroglia (OLG)-derived myelin (Sen et al., 2020), and many of the current therapeutical approaches in MS target different modes of action of immune cells (Tintore et al., 2019). Oligodendrocyte precursor cells (OPCs) in the adult CNS are recruited to MS lesions and are thought to contribute to remyelination during disease remission (Franklin and Ffrench-Constant, 2017), although this capacity is hindered upon disease progression (Jäkel et al., 2019; Yeung et al., 2019). OPCs transition to an immune-like state in the context of MS and demyelination (Absinta et al., 2021; Falcão et al., 2019; Fernández-Castañeda et al., 2020; Jäkel et al., 2019; Kirby et al., 2019). Immune OPCs can present antigens to CD4 (Falcão et al., 2019) and CD8 T cells (Fernández-Castañeda et al., 2020; Kirby et al., 2019).

This is an open access article under the CC BY license (http://creativecommons.org/licenses/by/4.0/).

Here, we have investigated the chromatin accessibility (CA) landscape at single-cell level in OLG in the experimental autoimmune encephalomyelitis (EAE) mouse model of MS, using single-cell assay for transposase accessible chromatin using sequencing (scATAC-seq) alone and in combination with scRNA-seq. We found that a cohort of immune genes increases CA in the context of EAE, whereas others already exhibit open chromatin in control (Ctr)-OLG, suggesting chromatin priming for these genes. Interferon gamma (IFN-γ) leads to their transcriptional activation, by a combination of increased CA, changes in the chromatin architecture, removal of histone marks, and/or regulation of the binding and activity of the transcription factors signal transducer and activator of transcription (STAT)1 and BTB and CNC homology (BACH)1. Single-cell multi-ome analysis of the healthy human brain indicated that chromatin priming occurs in several neural cell types. Moreover, MS susceptibility SNPs overlap with CA regions in OLG, some of which associated with genes regulated by IFN-γ, suggesting that MS risk might not be exclusively associated with immune cell types.

RESULTS

Differential CA allows identification of disease-specific OLG in the EAE mouse model of MS

We profiled CA of OLG at the single-cell level in EAE, by performing two iterations of scATAC-seq on the CNS of Sox10:Cre-RCE:LoxP(EGFP) mice (Falcão et al., 2019; Matsuoka et al., 2005; Sousa et al., 2009) induced with MOG35-55 peptide in complete Freud's adjuvant (CFA). The tissue was collected at the peak of EAE, score 3 or from control mice treated with CFA alone (CFA-Ctr) (Figure 1A). We sorted and pooled GFP$^+$ (labeling OLG) and GFP$^-$ cells 4:1 from freshly dissociated spinal cords of 4 CFA-Ctr and 2 EAE mice, before tagmentation and droplet generation (10x Genomics Chromium scATAC-seq, Figure 1A). We also used plate-based scATAC-seq (Pi-ATAC [Chen et al., 2018]), with fixed dissociated GFP$^+$ and GFP$^-$ cells from brains and spinal cords of 2 CFA-Ctr and 3 EAE mice tagmented and sorted in single wells, prior to barcoding and amplification (Figure 1A).

Clustering of the 10x scATAC-seq dataset, considering genome-wide differences in CA, led to the identification of 20 clusters (Figure 1B). CA at the *Sox10* locus was detected in all clusters except clusters 4, 10, 12, 15, 16, 17, and 18 (Figure S1A). Clusters 4, 10, 15, and 17 presented CA at the *Aif1* locus (marker for microglia and related cells, which might include macrophages [MiGl]). CA at the *Ptprz1* (marker of OPCs) and *Mog* (marker of mature oligodendrocyte [MOL]) loci was detected in smaller distinct subsets of clusters presenting CA at *Sox10* (Figure S1A). Notably, both OPC and MOL populations derived from EAE mice clustered separately from CFA-Ctr animals, whereas MiGl came exclusively from EAE (Figures 1C and 1D).

CA was observed mainly in intergenic (26.43%), intronic (48.83%), and promoter regions (13.84%) (Figure S1B). To classify cell types, we integrated EAE scRNA-seq data (Falcão et al., 2019; Figure S1C) with the scATAC-seq gene activities over promoter regions by identifying shared correlation patterns (Butler et al., 2018). We classified MiGl, vascular leptomeningeal cells (VLMCs), pericyte-like cells (PLCs), OPCs, and different MOL populations, segregated between CFA-Ctr and EAE (Figures 1D, S1D, and S1E). Similar results were obtained using Pi-ATAC, with a stronger EAE segregation in the spinal cord than in the brain (Figure S1F). Genes expressed in specific populations showed enriched CA in the scATAC-seq populations, for example, *Pdgfra* and *Ptprz1* for OPCs, *Mbp* and *Plp1* for MOLs, and *Aif1* and *Cxcr3* for MiGl (Figure S1G). These results indicate that OLG derived from EAE mice have an altered CA state.

Increased CA at promoters and enhancers of immune genes in single OPCs and MOLs in EAE mice

We inspected the genes that are closest to peaks differentially accessible in different OLG populations between CFA-Ctr and EAE mice, by performing gene ontology (GO)-analysis (ClueGO [Bindea et al., 2009] and using the Genomic Regions Enrichment of Annotations Tool (GREAT [McLean et al., 2010]; Table S1). Genes nearest to the regions with higher CA in EAE-OPCs were involved in "neuroinflammatory response," whereas in MOL1/2-EAE in "response to molecule of bacterial origin," among others (Figure S2A; Table S1), and in MOL5/6-EAE in "positive regulation of cytokine-mediated signaling pathway," "response to virus," among others (Figure S2A; Table S1). These GO terms indicate that CA changes in EAE-OLG populations are related to immune pathways.

We then investigated which gene loci have CA both in OPCs/OLs and MiGl, the resident immune cells in the CNS, and which are specific for each of these cell types. We found that while there is overlap between MiGl and OLG, most genes were unique to MiGl (Figure S2B). Gene set enrichment analysis (GSEA) (Borcherding et al., 2021) indicated that biological processes involved in IL6/JAK/STAT3 and TNF-α (via NF-κB) signaling are active specifically in MiGl, whereas IFN-γ and IFN-α responses are also induced in OPCs and MOLs in EAE (Figures 1E and S2C). The chromatin at gene loci involved in the inflammatory response is open in MiGl and, to some extent, in Ctr- and EAE-OPCs, unlike Ctr- or EAE-MOLs (Figure 1E), suggesting a differential chromatin immune profile within OLG, depending on their differentiation stage.

Cell-type-specific expression has been associated with distal enhancers, while promoters have been suggested to provide limited information (Heintzman et al., 2009; Thurman et al., 2012). We observed that 11.48% of differential CA peaks between OLG from EAE and CFA-Ctr mice were at promoter regions (Figure S1B). *Tnfrsf1a* (Figures 1F and S2D), a MS susceptibility gene encoding for TNF receptor superfamily member 1A (De Jager et al., 2009), had increased CA at the promoter in both MOL1/2-EAE and MOL5/6-EAE populations, which also presented increased gene expression in EAE scRNA-seq data (Falcão et al., 2019) (Figure S2E). Similar correlations could be observed at the major histocompatibility complex (MHC)-I locus (*Psmb9*/*Tap1* and *Psmb8*; Figures 1F, S2D, and S2E). *Ciita*, a master regulator of the MHC-II pathway, had EAE-OPC-specific CA at one intronic promoter (promoter IV, which is IFN-γ-responsive and active in non-professional antigen presenting cells [Muhlethaler-Mottet et al., 1997; Nikcevich et al., 1999]), whereas MiGl had CA at several promoter sites (Figure S2D). Thus, CA at promoters provides relevant information to predict cell types and possibly cell states.

CellPress
OPEN ACCESS

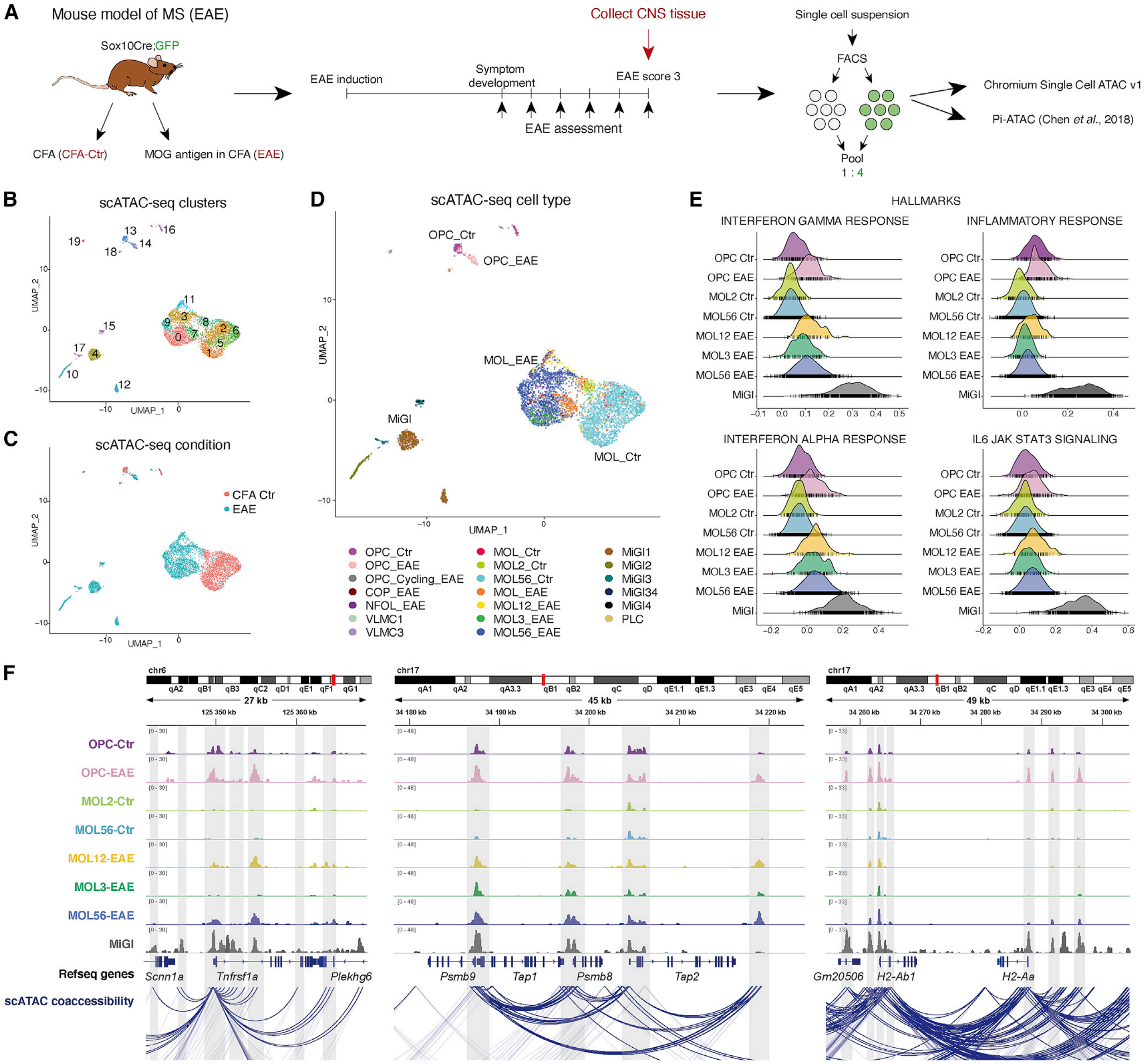

Figure 1. scATAC-seq reveals primed and increased CA at immune gene loci in OLG from the EAE mouse model of MS

(A) Experimental setup for scATAC-seq of OLG from the EAE mouse model of MS.

(B) Uniform manifold approximation and projection (UMAP) clustering based on CA of 10x Genomics chromium scATAC-seq.

(C) Origin of individual cells, projected on top of UMAP clustering.

(D) Label transfer from matched scRNA-seq data (Falcão et al., 2019) projected on top of CA UMAP.

(E) GSEA of the nearest genes to enriched CA peaks for immune Hallmarks categories.

(F) Integrative genomics viewer (IGV)-merged normalized tracks of CA with 100 randomly selected cells for each cluster, with MiGl clusters grouped together. scATAC co-accessibility connections are shown. Highlighted with gray boxes are regions with differential CA in specific clusters or promoter priming and connections between regulatory regions. Genomic coordinates are shown. OPC, oligodendrocyte precursor cell; VLMC, vascular leptomeningeal cell; PLC, pericyte-like cell; MiGl, microglia; NFOL, newly formed oligodendrocytes; MOL, mature oligodendrocyte.

Furthermore, 33.65% and 49.02% of differential CA peaks between OLG from EAE and CFA-Ctr mice were at intergenic and intronic regions, respectively (Figure S1B). Promoter-enhancer contacts can be estimated from scATAC-seq data by assessing chromatin co-accessibility. We calculated peak to gene interactions by correlating peak CA and gene expression data with ArchR (Granja et al., 2020; Pliner et al., 2018), allowing prediction of the overall promoter-enhancer interactome of OLG in EAE and CFA-Ctr. We observed CA at putative enhancers distal to immune genes in different EAE populations. Putative enhancers

at the *Tnfrsf1a* locus that linked to the *Tnfrsf1a* promoter had CA in MiGl only, in MiGl and EAE-OPCs, or in all EAE populations (Figures 1F and S2D). Although the promoter of MHC-I pathway gene *Tap2* did not have differential CA, it connected with a putative enhancer downstream that did present increased CA in EAE-OLG and linked to promoters of other MHC-I pathway genes in the same locus (Figures 1F and S2D). Similarly, MHC-II gene *H2-ab1*, which did not present increased CA at the transcription start site (TSS), linked to putative enhancers upstream and downstream with CA in MiGl and EAE-OPCs or in all EAE populations (Figures 1F and S2D). Thus, EAE-derived OLG have increased CA both at the promoters and enhancers of genes related to immune pathways.

Primed CA of immune genes in single OPCs and MOLs

We observed that several immune genes, such as *Psmb9*, *Tap1*, *Tap2*, and *H2-ab1*, with increased expression in EAE-OLG, were accessible in Ctr-OLG at their promoters (Figures 1F and S2D), suggesting chromatin priming. OPCs had more CA at immune genes than MOL in Ctr conditions (e.g., at the *Tap1* and *Tnfrsf1a* loci; Figures 1F and S2D), which suggests that OPCs might be more amenable to transition to immune states than MOLs. We then assessed the correlation of CA over 500-bp promoter regions (gene activity score) with gene expression from the scRNA-seq populations. Genes with increased expression in EAE and increased CA (Type1) could be divided into (1) genes with no or very low CA in Ctr, and (2) genes already presenting CA in Ctr. Top GO terms in OPCs (EAE versus Ctr) for Type1a genes were "response to IFN-β" and "negative regulation of viral genome replication," whereas Type1b genes were involved in "antigen processing and presentation of peptide antigen" (Figure 2A; Table S2).

Genes with increased expression in EAE-OPCs, but no change in CA (Type2), could also be divided in (1) genes that had high CA in Ctr and in EAE (Type2a; GO terms related to "mitotic cell cycle" and "DNA metabolic process" [Table S2]) and (2) genes with low CA in both Ctr and EAE (Type2b). Likewise, genes with higher expression in MOL1/2-EAE and MOL5/6-EAE, and increased (Type1) or similar (Type2) CA, are associated with immune processes (Figures 2A and S2F; Table S2). Type2a genes present a higher level of expression than Type1 genes in both OPCs and MOLs, which might be consistent with already open chromatin at promoters. Genes that lose expression in EAE-OPCs/MOLs, with GO terms related to the regulation of cilium assembly and metabolic processes, do not lose CA (Type3; Figures 2A and S2F).

We then assessed the distribution of immune genes found in GO terms as "immune response" (GO:0002250) and "immune system process" (GO:0002376) within the different RNA/CA types. Only a minority of these immune genes are expressed or present CA in OLG (Figure S2G; Table S2), consistent with the regulation of a small subset of immune genes in OLG when compared with MiGl (Figure S2B). Although up to a fourth of these genes were in the Type1a category, nearly half of the genes in OPCs, MOL1/2, and MOL5/6 in immune GO terms were found in the intermediate primed category (Type1b) and the primed category (Type2a), confirming that most of these immune genes are already primed at the chromatin level in OLG (Figure S2G; Table S2). We also analyzed the normalized aggregated scATAC coverage over the putative ArchR-predicted enhancers for Type1–Type4 genes (Figure 2A) and found that the primed state of the chromatin also applies to enhancers (Figure S2H).

We also performed simultaneous scATAC and RNA-seq (10x Genomics multi-ome) with *Sox10*-GFP cells sorted from the spinal cord of Ctr (2) and EAE mice (2, at the peak of disease) (Figure S3A). We identified the major OLG cell types, MiGl, but also astrocytes (Astro), (Figure 2B) and observed a clear segregation of Ctr versus EAE in the multi-ome RNA-seq dataset, which was not as pronounced in the multi-ome ATAC-seq dataset (Figures 2B and S3A). GSEA on the gene loci with accessible chromatin showed an enrichment within the hallmarks IFN-γ and IFN-α response for all the EAE-OLG populations, like the enrichment in MiGl (Figure S3B). Immune-related genes, such as *Ifit2*, *Nlrc5* (Figure 2C), *H2-Ab1*, *B2m*, and *Tap2* (Figure S3C), already exhibited CA in Ctr-OPCs and Ctr-MOLs, but with no or low expression in Ctr and induced expression in EAE. For other MHC-I related genes, such as *Psmb9*, *Tap1*, and *Psmb8*, an increase in CA and an induction of expression occurred simultaneously in EAE-MOLs (Figure S3C), which confirms our previous findings with unimodal scATAC-seq and scRNA-seq. Thus, our analysis indicates that Ctr-OLG are already in a primed immune chromatin state, with the observed increased expression of primed immune genes in EAE most likely controlled by mechanisms other than CA.

IFN-γ modulates CA only in a subset of immune genes in OPCs

IFN-γ induces expression of immune genes in OPCs, like the phenotype observed in EAE (Falcão et al., 2019; Kirby et al., 2019). IFN-γ response is one of the hallmarks induced in EAE-OLG at the CA level. To investigate whether modulation of CA is involved in IFN-γ-mediated activation of immune transcriptional programs, we treated primary mouse OPCs with IFN-γ for 48 h and performed bulk ATAC-seq and RNA-seq. Interferon response genes *Stat1*, *Stat2*, and *Irf1*, and MHC-I and MHC-II genes *H2-k1*, *H2-q7*, *H2-ab1*, and *H2-aa*, among other genes involved in immune regulation, presented increased expression (Figures 3A and S4A–S4C).

There were fewer CA sites altered upon IFN-γ treatment (47 with $\log_2$ fold change ≥ 1.5), when compared with the number of genes with altered expression (867) (Figure 3A; Table S3). Nevertheless, we found similar GO terms when analyzing peaks within proximal (promoter) regions of genes, including "response to IFN-β" and "defense response to protozoan" (Figure S4D; Table S3). We also found more changes in CA at annotated enhancers (463) (Figure S4E; Table S3). Fewer genes were downregulated upon IFN-γ treatment (171 versus 696) (Figure 3A), with GO terms related to system and CNS development (Figure S4C; Table S3). Among these genes were *Sox8*, *Myrf*, and *Plp1* (Figure S4F), consistent with the negative impact of IFN-γ on OPC differentiation (Chew et al., 2005; Kirby et al., 2019). We did not find any loss of CA at their promoters or annotated enhancers (Figure S4F).

A larger subset of the upregulated immune response genes already had CA at their promoter in control OPCs (272 Type2), and only a few genes altered their CA upon IFN-γ exposure (78

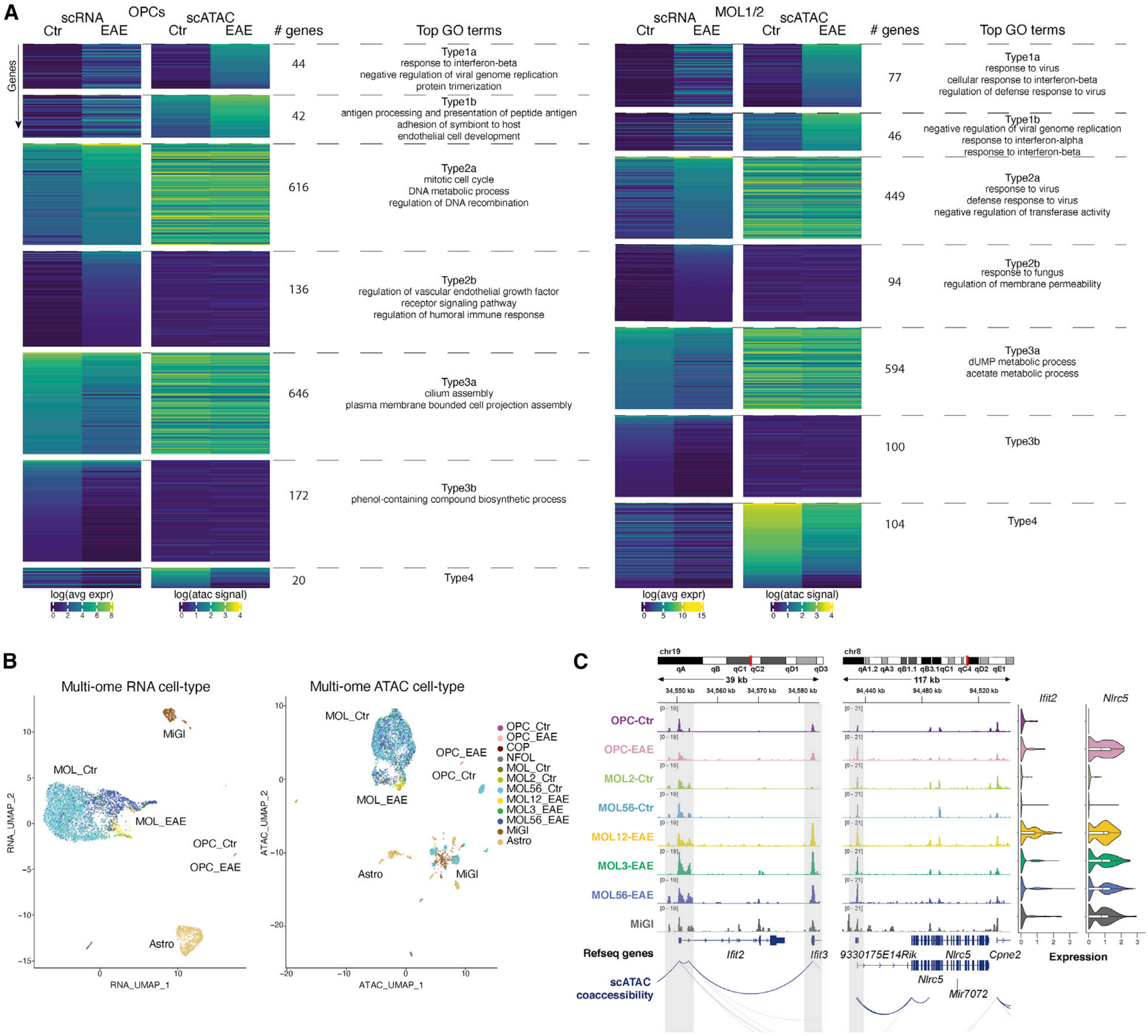

Figure 2. Primed CA of immune genes in single OPCs and MOLs

(A) Genes in OPCs (left) or MOL1/2 (right) are clustered based on gene expression differences between EAE versus Ctr and correlation with CA activity score (CA over 500-bp promoter region). Top GO terms for Type1 genes (with increased expression and CA in EAE ; [Type1a] low and [Type1b] high CA in Ctr-OPCs), Type2 (with increased expression in EAE, but no change in CA, [Type2a] high and [Type2b] low CA in Ctr-OPCs), and Type3 (with reduced expression in EAE, but no change in CA). Type4 (with reduced expression and CA in EAE) had no GO terms.

(B) UMAP based on CA (right) and RNA-seq (left) of 10x Genomics multi-ome (simultaneous scATAC and RNA-seq) of *Sox10*-GFP cells sorted from the spinal cord of Ctr (2) and EAE mice (2, at disease peak). Label transfer from matched scRNA-seq data (Falcão et al., 2019).

(C) (Left) IGV tracks of CA for each selected cluster, with MiGl clusters grouped together. scATAC co-accessibility connections are shown. Highlighted with gray boxes are regions with differential CA in specific clusters or promoter priming and connections between regulatory regions. Genomic coordinates are shown. (right) Violin plots depicting the expression of *Ifit2* and *Nlrc5* in each cluster. Cell acronyms as in Figure 1.

Type1) (Figure 3B). We could again divide the Type1 and Type2 in two subgroups. The top GO terms for those type of genes that were already accessible in Ctr but had both increased CA and expression (Type1b, 56 genes) were “antigen processing and presentation of peptide antigen,” “defense response to virus,” and “response to IFN-β” (Figure 3B; Table S3). Type2a genes, which had high CA in Ctr that did not change upon IFN-γ treatment while their expression was induced, had top GO terms as “positive regulation of proteolysis,” “IκB kinase/NFκB signaling,” and “positive regulation of apoptotic signaling pathway,” and Type2b genes, which had low CA, had top GO terms as “cellular response to IFN-β” and “negative regulation of innate immune response” (Figure 3B; Table S3). Some of the Type2 genes, such as *Irf1*, *Stat3*, and *Ifit2*, had enhanced

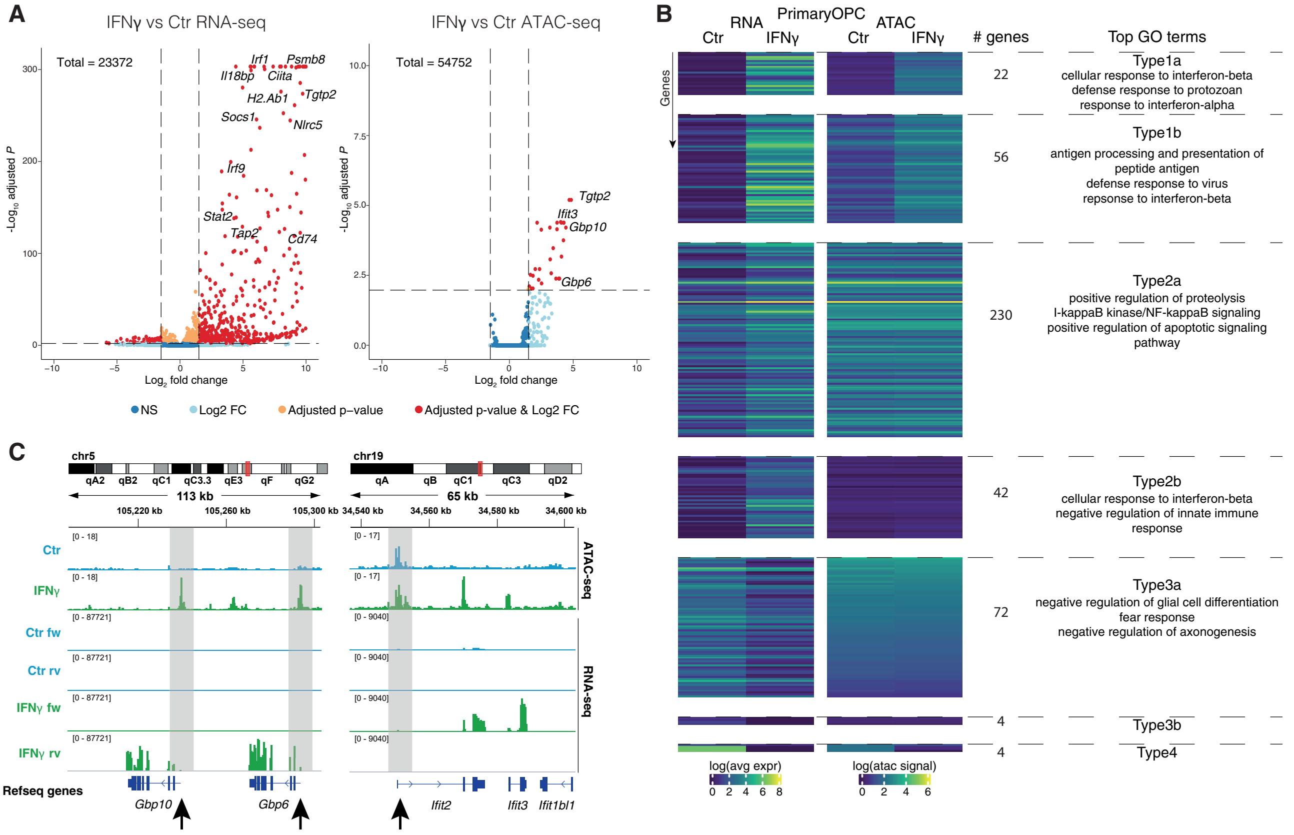

Figure 3. Primed CA at immune gene loci in primary mouse OPCs

(A) Volcano plots showing differential gene expression in RNA-seq (left) and CA at promoter regions in ATAC-seq (right) between Ctr-OPCs and OPCs treated with 100 ng/mL IFN-γ for 48 h. Genes with adj. p value < 0.05 and $\log_2$ fold change >1.5 are shown in red.

(B) Genes in IFN-γ-treated OPCs and Ctr-OPCs are clustered based on their chromatin activity score (CA over 500-bp promoter region) and gene expression correlation. Top GO terms are shown for Type1–Type4 as defined in Figure 2A.

(C) IGV tracks are shown for ATAC-seq and RNA-seq in IFN-γ-treated OPCs and Ctr-OPCs for selected genes. Highlighted with gray boxes and arrows are regions with differential CA in IFN-γ-treated OPCs or promoter priming; black arrows, promoter regions. Merged tracks of 3 biological replicates are shown for ATAC-seq and 4 biological replicates for RNA-seq.

CA within their gene body or in the regions upstream of the TSS, pointing toward promoter priming and specific enhancer regulation of expression. Other genes, such as the *Gbp* gene family, presented increases in both CA and gene expression upon IFN-γ treatment (Figure 3C).

Although there were communalities between the immune regulation observed in OPCs *in vivo* in EAE and the effects of IFN-γ in OPCs *ex vivo*, there were also differences within the different Types (Figures 2A and 3B; Tables S2 and S3). Intersection between OPCs *in vivo* in EAE and OPCs upon IFN-γ treatment shows low overlap between genes associated to the different Types, where Type2a genes showed the highest intersection with 22 genes shared between EAE-OPCs and IFN-γ-treated OPCs (Figure S4G). The environment surrounding OPCs in EAE is complex, due to exposure to additional cytokines other than IFN-γ, which might contribute to the observed differences. Nevertheless, our data indicate that OPCs already exhibit primed CA in a large subset of immune genes, prior to inflammatory insults both *ex vivo* and *in vivo*.

Transcription factors involved in immune regulation have increased motif accessibility during the transition to immune OLG

Since transcription factors (TFs) are key modulators of transcription, we applied chromVAR (Schep et al., 2017) to the 10x scATAC-seq dataset to determine TF motif variability in EAE. As expected, we observed clusters of TFs of the Ets and AP-1/bZIP families with enriched accessible motifs in MiGl, whereas members of the SRY-related HMG-box (SOX) and basic helix-loop-helix (bHLH) families had increased motif accessibility (MA) in Ctr-OPCs and Ctr-MOLs (Figure 4A; Table S4). We found differential TF activity for 147 (OPCs), 105 (MOL1/2), and 94 (MOL5/6) non-redundant motifs between EAE and Ctr, respectively (fold change ≥1; adjusted p value < 0.05), including FOS, SMARCC1, and TFs with known immunoregulatory functions, such as the TF BTB and CNC homology (BACH)1, BACH2, and the basic leucine zipper ATF-like TF BATF (Figures 4A, 4B, S5A, and S5B; Table S4).

We analyzed which TF motifs were overrepresented in EAE compared with CFA-Ctr in OPCs, MOL1/2, and MOL5/6

CellPress
OPEN ACCESS

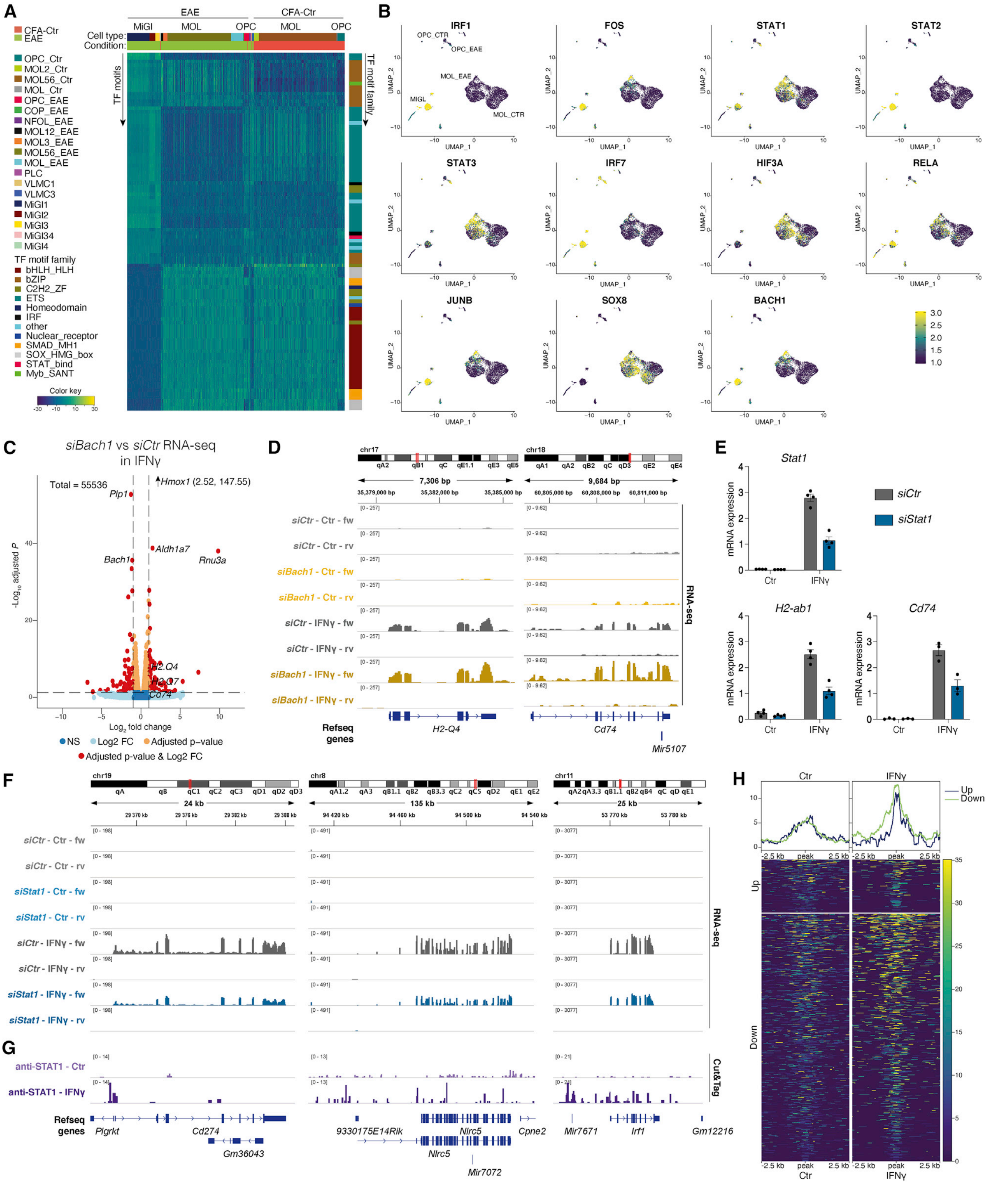

Figure 4. STAT1 and BACH1 have increased motif accessibility (MA) in OLG from EAE and are involved in IFN-γ-mediated regulation of immune genes in OPCs

(A) ChromVAR clustering of TF motif variability from scATAC-seq. Each row presents a TF motif, whereas each column represents a single cell. Scale, blue (low TF MA) to yellow (high).

(legend continued on next page)

specifically. IRF1, STAT2, and KLF4 had enriched motifs in EAE in all three OLG populations and had predicted binding sites in EAE in promoter and enhancer regions of the *Psmb9*-*Tap2* locus (Figures S5B and S5C). STAT1, STAT3, HIF3a, RELA, SMARCC1, and NFIX presented differential MA in most of OLG populations in EAE, whereas other TFs were particularly enriched in specific EAE-OLG populations, such as IRF1 and STAT2 in EAE-OPC, IRF7, FOS, JUNB, KLF13, KLF4, BACH1, and BACH2 in MOL1/2-EAE and SOX8 in all EAE-MOL populations (Figures 4B and S5A).

A subset of TFs in OLG showed low expression or were not expressed at all in Ctr and EAE (67 in OPCs, 136 in MOL1/2, and 210 in MOL5/6 TFs were expressed in less than 50% of cells in both Ctr and EAE), such as *Bach2* in OPCs, *Bach1* in MOL1/2, and *Klf4* in MOL5/6 (Figure S5B), indicating that they will not be driving the MA changes. However, some of the identified TFs did exhibit increased expression in OLG from EAE mice (22 TFs in OPCs, 26 TFs in MOL1/2, and 21 TFs in MOL5/6 with delta EAE-Ctr expression >0.1; Figure S5B). In addition, a subset of these TFs had similar expression in Ctr- and EAE-OLG (40 TFs in OPCs, 77 TFs in MOL1/2, and 187 TFs in MOL5/6 with delta EAE-Ctr expression <0.1), such as *Sox8* (in MOL1/2), *Smarcc1* (in OPCs and MOL1/2, but not MOL5/6), and *Nfix* (Figure S5B). Thus, TFs as SOX8, implicated in OLG development (Stolt et al., 2004) and MS susceptibility (International Multiple Sclerosis Genetics Consortium, 2019), might work in concert with TFs other than the ones they usually cooperate with during development and homeostasis, in order to regulate immune gene transcription in OLG in EAE/MS.

STAT1 and BACH1 are involved in IFN-γ-mediated regulation of immune genes in OPCs

BACH1, which regulates inflammatory macrophage differentiation (Igarashi et al., 2017), is expressed in both Ctr- and EAE-OPCs, presenting increased MA in the latter and, despite very low expression, also in MOL1/2 (Figures 4B and S5B). We transfected primary OPCs with siRNAs targeting *Bach1* before treating with IFN-γ for 6 h (Figure S6A, n = 4). qRT-PCR and bulk RNA-seq of IFN-γ-treated OPCs upon *Bach1* knockdown indicated downregulation of the target gene and upregulation of *Hmox1* (Figures 4C and S6B–S6D; Table S5), consistent with the role of BACH1 as a transcriptional repressor of *Hmox1* (Igarashi et al., 2017). We also observed an increased expression of MHC-I genes, such as *H2-Q4*, *H2-Q7*, and MHC-II pathway gene *Cd74*, upon IFN-γ treatment (Figures 4C, 4D, and S6D; Table S5). Thus, our data suggest that BACH1 negatively regulates IFN-γ-mediated induction of a subset of immune genes in OPCs.

STAT1 is a major transducer of IFN-γ signaling in immune cells (Hu and Ivashkiv, 2009; Wang et al., 2010). Since we observed that STAT1 expression and MA are increased in EAE-OPCs (Figures 4B and S5B), we transfected primary OPCs with siRNAs targeting *Stat1*, followed by IFN-γ treatment for 6 h (Figure S6A, n = 4). qRT-PCR analysis indicated a decreased expression of *Stat1* (Figure 4E). In contrast to the *Bach1* knockdown, we observed a reduction of the MHC-II-related genes *H2-ab1* and *Cd74* mRNA levels by qRT-PCR upon *Stat1* knockdown (Figure 4E). Bulk RNA-seq of *Stat1* knockdown in IFN-γ-treated OPCs led to a reduced expression of immune-related genes as *Il12rb1*, encoding interleukin-12 receptor, beta 1, *Irf8*, *Cd274*, encoding for PD-L1, *Irf1*, *Nlrc5*, *Tap1*, and *Psmb8* (Figures 4F and S6E–S6H; Table S5). These data suggest that STAT1 is involved in the IFN-γ-mediated upregulation of immune genes in OPCs.

We analyzed the binding of STAT1 in OPCs by performing CUT&Tag (Kaya-Okur et al., 2019). STAT1 binding at regulatory regions of the immune-related genes identified by bulk RNA-seq was increased in OPCs upon IFN-γ treatment (Figures 4G and S6I). Moreover, genes that were up- or downregulated by STAT1 knockdown had increased binding of STAT1 in OPCs treated with IFN-γ compared with Ctr-OPCs (Figures 4G, 4H, and S6I). Thus, our data indicate that TFs as BACH1 and STAT1 participate in the regulation of IFN-γ-induced immune gene expression in OPCs.

Enhancer-promoter contacts at immune genes in OPCs are altered upon IFN-γ treatment

Mechanisms other than increased CA must be required for the transcriptional activation of primed immune genes in the context of EAE/IFN-γ treatment. We first profiled H3K27ac with Cut&Run (Skene and Henikoff, 2017) to identify active enhancers. An increase of H3K27ac was observed at enhancers and promoters of immune genes, such as *Tgtp1/2*, *Zbp1*, *Gbp9*, *Cd74*, *Nlrc5*, *H2-aa*, and *H2-eb1* (Figure 5A; Table S6). CCCTC-binding factor (CTCF) is required for acute inflammatory responses in macrophages (Stik et al., 2020), and an increase in CTCF-mediated promoter-enhancer interactions at the MHC-I and MHC-II loci occurs in B cells (Majumder and Boss, 2010; Majumder et al., 2014). We observed increased binding of CTCF in enhancers and at the promoters of many immune genes (Figure 5A; Table S6).

To evaluate whether promoter-enhancer interactions were modulated by IFN-γ treatment, we performed HiChIP (Mumbach et al., 2016) targeting H3K27ac in OPCs treated with IFN-γ. We used the activity-by-contact (ABC) model (Fulco et al., 2019) to predict promoter-enhancer interactions in both Ctr- and IFN-γ-OPCs based on CA and H3K27ac-HiChIP. We found that

(B) TF motif variability projected on top of UMAP of scATAC-seq.
(C) Volcano plots showing differential expressed genes in RNA-seq upon transfection of primary OPCs with siRNAs targeting *Bach1* before treating with IFN-γ for 6 h. 4 biological replicates. Genes with adj. p value < 0.05 and $\log_2$ fold change >1.5 are shown in red. *Hmox1* coordinate values are shown (since beyond y-axis range).
(D) IGV tracks for RNA-seq in IFN-γ-treated OPCs and Ctr-OPCs after transfection with siRNAs targeting *Bach1* for selected genes.
(E) qRT-PCR targeting selected genes upon transfection of primary OPCs with siRNAs targeting *Stat1* before treating with IFN-γ for 6 h. 3–4 biological replicates. Error bars, mean ± SEM.
(F) IGV tracks for RNA-seq in IFN-γ-treated OPCs and Ctr-OPCs after transfection with siRNAs targeting *Stat1* for selected genes.
(G) IGV tracks showing STAT1 binding in OPCs upon IFN-γ treatment and in Ctr-OPCs, assessed with CUT&Tag. 3 biological replicates.
(H) Heatmaps depicting STAT1 binding in OPCs treated with IFN-γ at genes that were up- or downregulated by STAT1 knockdown (as assessed by adjusted p value from bulk RNA-seq), centered at peaks.

CellPress
OPEN ACCESS

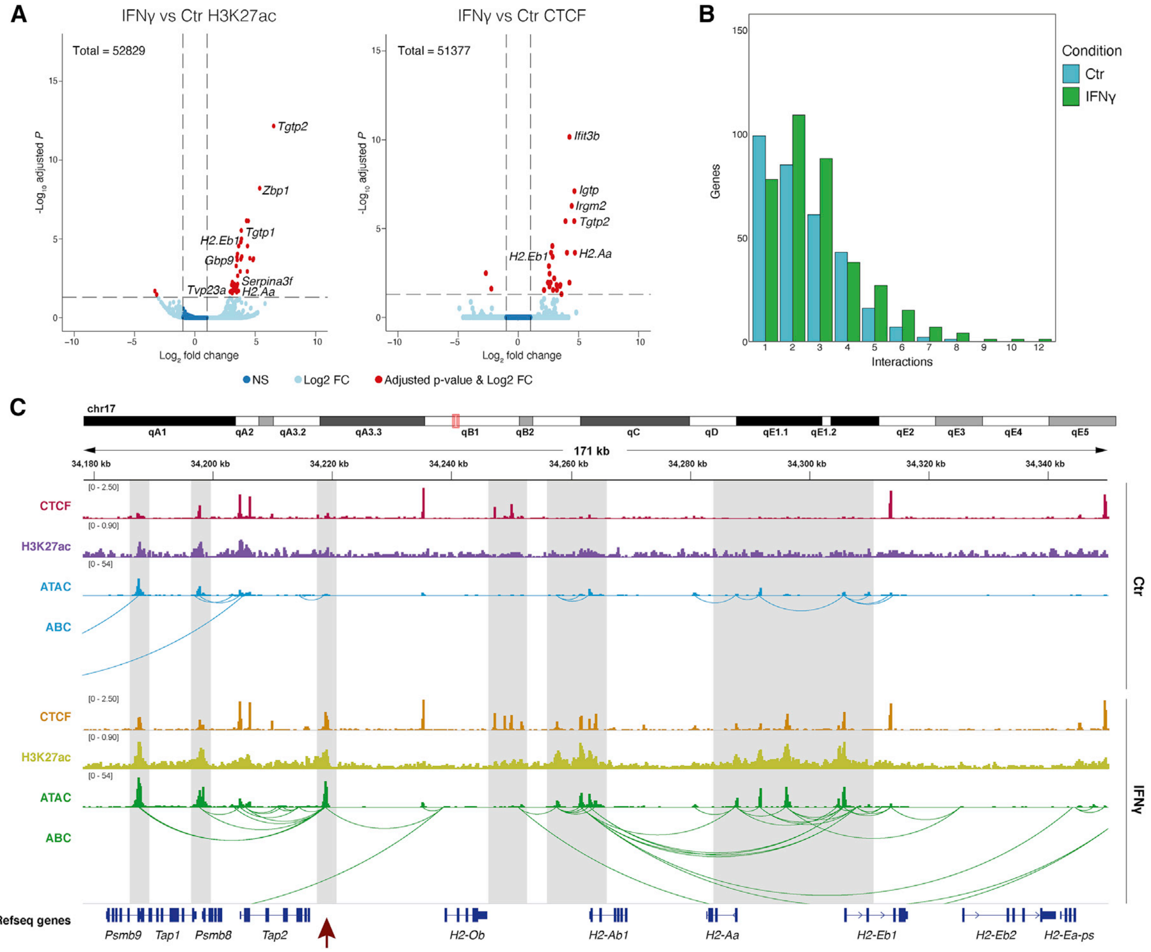

Figure 5. H3K27ac, CTCF binding, and enhancer-promoter contacts at immune genes in mouse OPCs are altered upon IFN-γ treatment

(A) Volcano plots showing differential H3K27ac (left) and CTCF binding (right) between IFN-γ-treated OPCs and Ctr-OPCs, assessed with Cut&Run. 3 biological replicates. Genes with adj. p value < 0.05 and log_2 fold change >1.5 are shown in red.

(B) Number of genes (y axis) in Ctr-OPCs (blue) and IFN-γ-treated OPCs (green) with n predicted interactions (x axis).

(C) IGV tracks showing CTCF binding and H3K27ac occupancy, assessed with Cut&Run, ATAC-seq in IFN-γ-treated OPCs, and Ctr-OPCs for MHC-I and MHC-II loci. Predicted enhancer/promoter contacts computed by the activity-by-contact (ABC) model (Fulco et al., 2019) based on CA and H3K27ac-HiChIP. Highlighted with gray boxes are regions with increased H3K27ac, CTCF binding, CA, and/or predicted interactions in IFN-γ-treated OPCs. Highlighted with a red arrow is an enhancer region interacting with multiple genes in the MHC-I and MHC-II loci. Merged tracks for 3 biological replicates per condition.

46.26% (322 genes, 223 upregulated and 99 downregulated) of differentially expressed genes upon IFN-γ treatment exhibited reconfiguration of the enhancer-promoters contacts (Figure 5B; Table S6). For instance, we observed an increase in predicted interactions at the *Psmb9-Tap2* locus (Figure 5C). An enhancer downstream of *Tap2* harbors a STAT1/STAT2 motif (Figure S5C) with STAT1 binding in IFN-γ-OPCs (Figure S6I), which also has increased CA and H3K27ac upon IFN-γ treatment and might be the major enhancer connecting with all the genes in the locus (Figure 5C). The CA (Figure 1F) and STAT2 MA (Figure S5C) at this enhancer were also increased in EAE-OLG. Interactions between numerous enhancers and promoters in the neighboring *H2-ab1–H2-eb1* loci were also increased in IFN-γ-OPCs (Figure 5C).

We analyzed the normalized ATAC coverage over the putative enhancers predicted by ABC for Type1–Type4 genes (Figure 3B). There was an increase in CA upon IFN-γ treatment versus Ctr in enhancers at Type1 genes, but we also observed an increase at Type2 genes (Figure S7A), suggesting that CA at enhancers might play a role in transcriptional activation. These results suggest that altered CA, together with increased CTCF binding, H3K27ac deposition, and promoter-enhancer interactions, might contribute to the activation of an immune gene transcriptional program in primed OPCs.

H3K27me3 modulation is involved in IFN-γ-mediated immune gene activation in OPCs

H3K4me3/H3K27me3 bivalency mediates poising of cell-type-specific transcription during development (Bernstein et al., 2006) and immune gene transcription in cancer (Burr et al., 2019). Hence, H3K27me3-mediated repression of poised genes could be involved in immune gene regulation in OPCs. We used

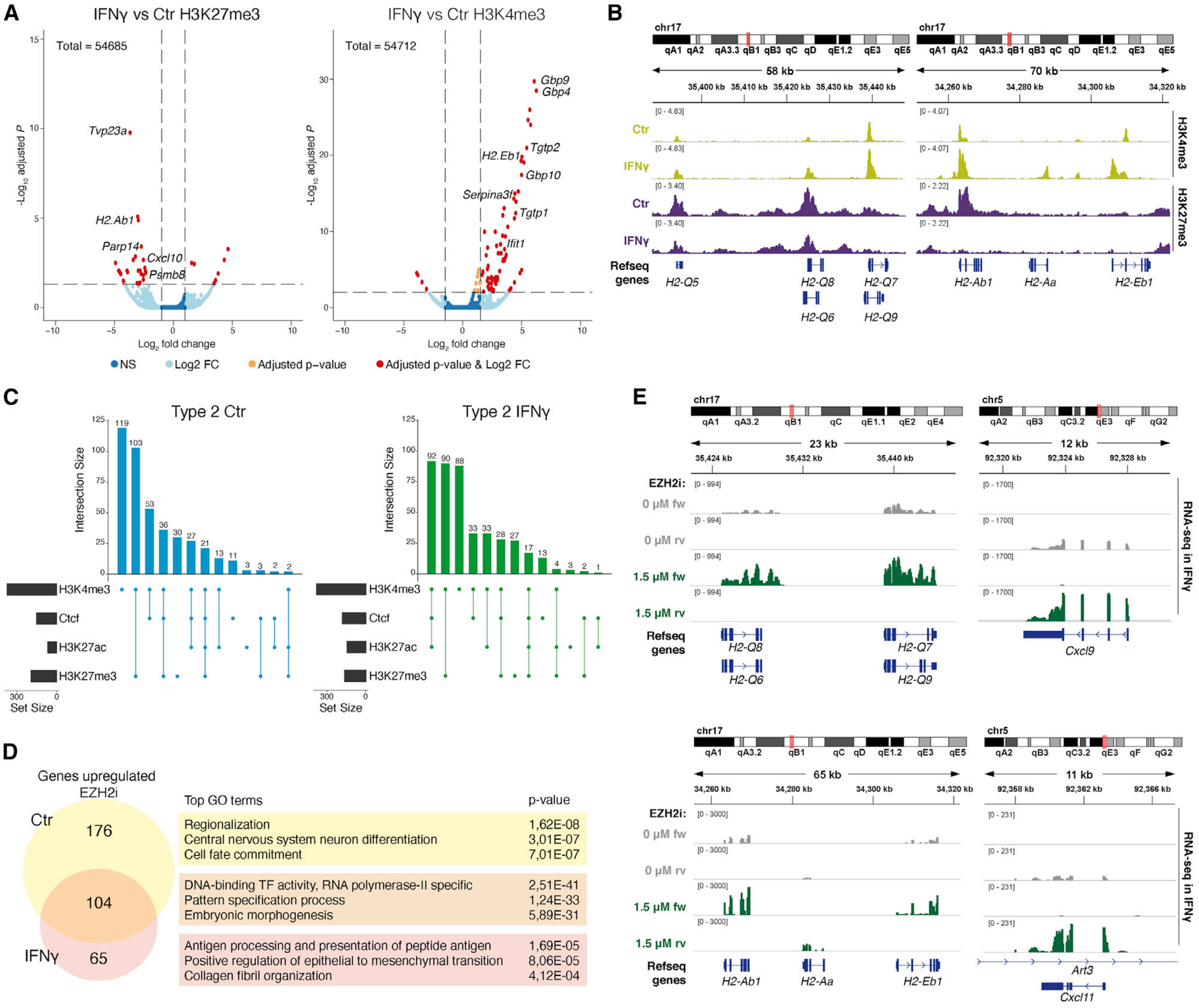

Figure 6. H3K27me3 modulation is involved in IFN-γ-mediated immune gene activation in OPCs

(A) Volcano plots for H3K27me3 and H3K4me3 in IFN-γ-treated OPCs versus Ctr-OPCs assessed with Cut&Run. Two replicates. Genes with adj. p value < 0.05 and $\log_2$ fold change >1.5 are shown in red.

(B) Cut&Run IGV tracks for selected MHC-I and MHC-II genes with increased H3K4me3 or decreased H3K27me3 in OPCs upon IFN-γ treatment. Merged tracks of two replicates.

(C) Upset plots of Cut&Run Ctr-OPCs and IFN-γ-treated peak intersection in Type2 genes. Top barplot shows the number of intersecting peaks per combination, left barplot shows the size of each peak dataset, and the matrix shows the Cut&Run peak sets (dots) and shared (connecting line) in each combination.

(D) Venn diagram showing the number of genes enriched in OPCs upon treatment with 1.5 μM EZH2 inhibitor EPZ011989 (EZH2i) for 4 days, with and without subsequent co-treatment with 100 ng/mL IFN-γ for last 6 h (and the genes enriched in both) and the top gene ontology biological terms for the genes in each category.

(E) RNA-seq IGV tracks for MHC-I, MHC-II, and cytokine genes with increased expression upon EZH2i in IFN-γ-spiked OPCs. Merged tracks of three biological replicates.

Cut&Run to profile H3K27me3 and H3K4me3 in primary OPCs. In Ctr-OPCs, H3K27me3 and H3K4me3 bivalency were indeed observed at the promoters of MHC-I and -II genes, including *Tap1*, *H2-q5*, *H2-q6*, *H2-q7*, *H2-k1*, and *H2-ab1* (Table S6). Strikingly, upon 48 h of IFN-γ treatment, H3K27me3 was reduced at immune gene loci, including *H2-ab1*, *Cxcl10*, and *Psmb8* (Figures 6A and 6B; Table S6). An increase of H3K4me3 is observed at immune genes such as *Gbp4*, *Gbp9*, *Tgtp1*, *Tgtp2*, *Serpina3f*, *H2-ab1*, *H2-aa*, and *H2-eb1* (Figures 6A and 6B; Table S6). Type1 genes are mostly marked by H3K4me3 in Ctr and by a combination of H3K4me3, CTCF, and H3K27ac in IFN-γ, with limited occupancy of H3K27me3 (Figure S7B). Type2 genes are mostly marked with H3K4me3 or a combination of H3K4me3 and H3K27me3 in Ctr and a combination of H3K4me3, CTCF, and H3K27ac, or H3K4me3 and H3K27me3 or H3K4me3 alone in IFN-γ (Figure 6C). Thus,

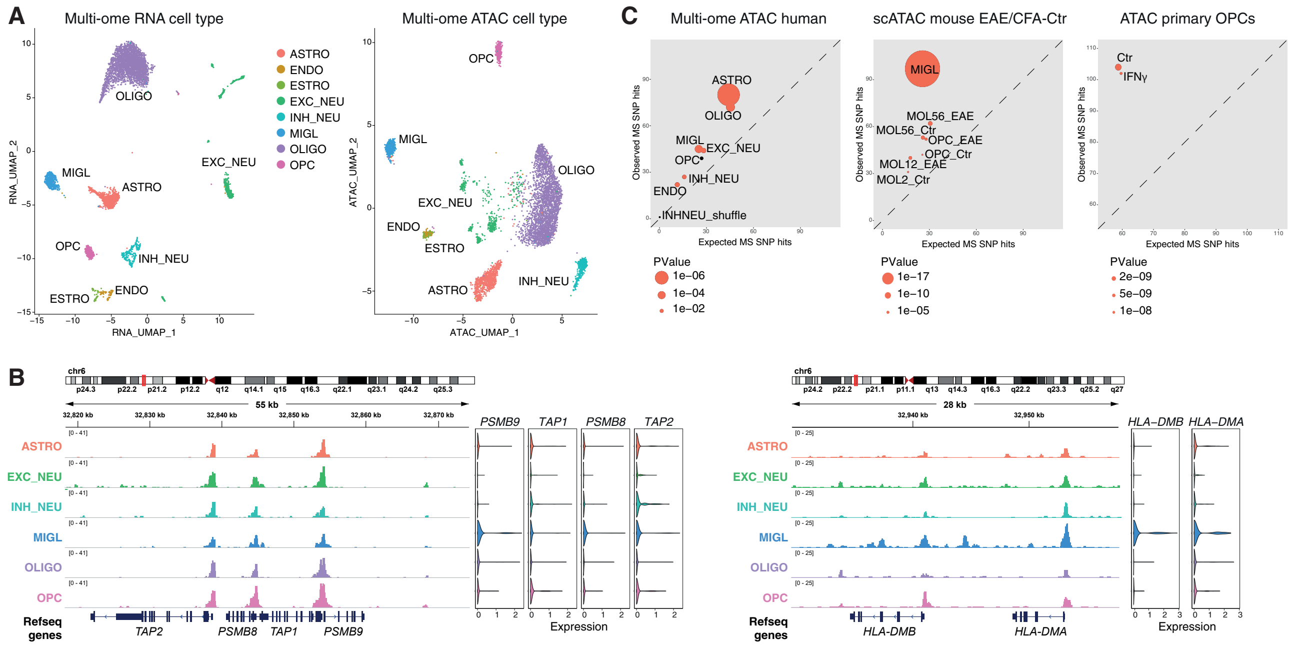

Figure 7. Primed CA at immune gene loci in human neural cells

(A) UMAP based on CA (right) and RNA-seq (left) of 10x Genomics multi-ome of brain gray matter from two healthy individuals.

(B) IGV tracks of CA for each selected cluster (hg38). Violin plots depicting the expression of selected genes in each individual cluster.

(C) Overlap with MS-associated GWAS variants. For each peak set, expected (x axis) versus observed (y axis) number of SNP hits overlapping the human healthy individuals hg19 multi-ome scATAC-seq peaks, scATAC-seq cell-type-specific peaks from Ctr and EAE mice, and Ctr-OPC and IFN-γ-treated primary OPC ATAC-seq peaks. Dot size scaled to adjusted p value and adjusted p values < 0.01 in red. ENDO, endothelial cells; ASTRO, astrocytes; EXCNEU, excitatory neurons; INHNEU, inhibitory neurons; MIGL, microglia; OLIGO or MOL, mature oligodendrocyte; OPC, oligodendrocyte precursor cell; VLMC, vascular leptomeningeal cell; PLC, pericyte-like cell.

resolving bivalency might contribute to the activation of transcription of Type2 immune genes in OPCs upon IFN-γ treatment, combined with changes at the enhancer-promoter level.

Inhibition of EZH2, the enzyme responsible for the deposition of H3K27me3 (Figure S7C), with EPZ011989 (Burr et al., 2019) (EZH2i) led to a reduction of H3K27me3 overall (Figure S7D) and at specific genomic loci (Figures S7E and S7F) and a derepression of TFs involved in specification and morphogenesis (Figures 6D and S7G; Table S7). Importantly, EZH2i led to an upregulation of the expression of a subset of immune genes, such as *H2-d1* and *Tap1*, whereas others remained repressed. Spiking EZH2-inhibited cells with IFN-γ for 6 h led to an increased upregulation of MHC-I and MHC-II genes *H2-q6*, *H2-q7*, *H2-ab1*, *H2-aa*, and *H2-eb1* and genes involved in cytokine signaling including *Cxcl9* and *Cxcl11* (Figures 6E, S7G, and S7H; Table S7). These results suggest that H3K27me3 removal synergizes with IFN-γ to promote immune gene transcription in OPCs.

SNPs and outside variants conferring susceptibility in MS are located at accessible regulatory regions in OLG in the mouse and human CNS

To investigate the CA of immune genes in the human brain, we performed simultaneous scATAC and RNA-seq (10x Genomics multi-ome) in brain gray matter from two healthy individuals, identifying the major cell types present in the CNS in both modalities (Figures 7A and S8A). Strikingly, the promoters of MHC-I pathway genes *TAP2*, *PSMB8*, *TAP1*, *PSMB9*, and *HLA-B* were accessible not only in MIGL, but also in OPCs, MOLs, ASTRO, inhibitory neurons (INHNEU), and excitatory neurons (EXCNEU) (Figures 7B and 8B). Nevertheless, *PSMB9* and *PSMB8* were only expressed in MIGL although at very low levels, whereas *TAP2* was expressed in most cell types except MOLs and EXCNEU (Figure 7B). There was more specificity for MHC-II-related genes. For *HLA-DMB*, MIGL, OPCs, and EXCNEU had open chromatin, whereas ASTRO, MOLs, and INHNEU had lower levels of CA, with the gene expressed only in MIGL (Figure 7B). For *HLA-DBQ1* and *HLA-DRB1*, MIGL and neurons had CA at promoter regions, with expression restricted to MIGL (Figure 8B).

Analyzing Type1 genes from mouse OLG (Figure 2A) in the human multi-ome dataset, we find that most genes are expressed and accessible in human MIGL and to a lesser extent also in ASTRO, but not in OLG (except a small subset in OPCs), in concordance with their low expression in mouse Ctr-OPC and Ctr-MOL (Figure S8C). Type2 genes show the same expression pattern in human but are accessible in all cell types (Figure S8C). We also analyzed snATAC-seq from the adult human brain from 10 healthy individuals (Corces et al., 2020). As in the multi-ome data, we observed CA at the regulatory regions of MHC-I-related genes in OLG, but not in MHC-II genes (Figure S8B). Thus, our data suggest that chromatin priming at a subset of immune genes occurs in human OLG and in other neural cell types.

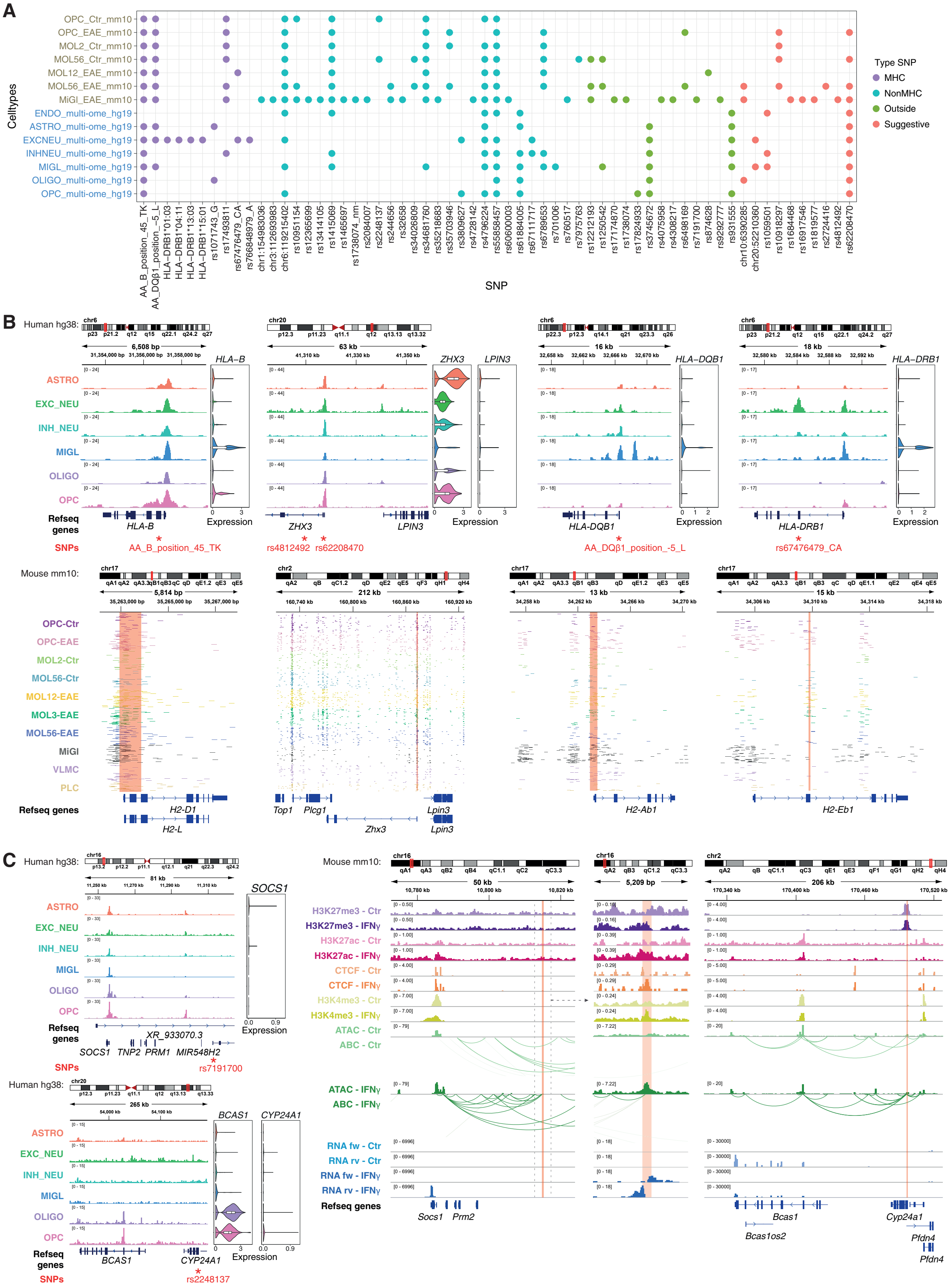

(legend on next page)

CellPress
OPEN ACCESS

Given that some of these immune loci bear SNPs associated with MS susceptibility, we assessed whether we could identify putative MS susceptibility loci associated with CA in OLG. We cross-referenced the coordinates of mouse OLG and MiGl peaks from the EAE scATAC experiments with the location of MS susceptibility SNPs (International Multiple Sclerosis Genetics Consortium, 2019) and outside variants (Factor et al., 2020). Linkage disequilibrium score regression (LDSC) (Finucane et al., 2018), multi-marker analysis of genomic annotation (MAGMA) (de Leeuw et al., 2015), and genomic regulatory elements and GWAS overlap algorithm (GREGOR) (Schmidt et al., 2015) indicated an enrichment at chromatin accessible regions and associated genes mainly in MiGl, consistent with association of MS susceptibility with gene expression in MIGL (Falcão et al., 2019; International Multiple Sclerosis Genetics Consortium, 2019) (Figures 7C and S8D; Table S8). We also found enrichment for OLG, although with not the same significance level as for MiGl (LDSC: MiGl versus All: coefficient p value 1.05E−6; MOL5/6 [Ctr versus EAE], coefficient p value 0.008834828; OPC [Ctr versus EAE] coefficient p value 0.048301018). Similar results were obtained for MAGMA, although only for the mouse scATAC-seq EAE dataset (Table S8). GREGOR analysis on the human multiome regulatory regions suggested an overrepresentation of MS-associated SNPs in ASTRO and OLIGO (Figure 7C), whereas the same analysis on the human scATAC data suggested enrichment in MiGl regulatory regions, but also in neural cells (Figure S8D; Table S8). This analysis also indicated an enrichment to a lesser extent in OLG from the mouse EAE and Ctr spinal cord (Figure 7C; Table S8). Moreover, mouse primary OPCs, regardless of treatment with IFN-γ, also presented enrichment of CA with SNPs associated with MS susceptibility (Figure 7C).

Analysis of multi-ome data and CA (scATAC-seq) data from adult human brain derived from healthy individuals revealed that these MS susceptibility SNPs overlapped with CA in diverse CNS cell types in a non-disease context (Figure 8A). We found that AA_DQβ1_position_-5_L (*HLA-DQB1/H2-Ab1*) (Figure 8B), rs10918297 (*UCK2/Uck2*), and rs35703946 (*IRF8/Irf8*) SNPs overlap in OLG-specific ATAC peak regions, in human and/or mouse (EAE), and are classified as Type2a genes for MOL1/2 (*H2-Ab1*, *Uck2*) and OPC (*Irf8*) (Tables S2 and S8). *Irf8* is an intermediate primed gene (Type1b) in MOL1/2 and MOL5/6 (Table S2). In addition, SNPs located at the human MHC locus *HLA-B* (SNP ID AA_B_position_45_TK) and the non-MHC locus *ZHX3* (SNP ID rs62208470) coincided with CA in human MiGl and in OLG and at the corresponding mouse loci (*H2-d1/H2-l and Zhx3*) in both EAE and CFA-Ctr (Figures 8A, 8B, and S8B; Table S8).

MHC SNPs such as SNP ID AA_DQβ1_position_-5_L at *HLA-DQB1* exhibited CA in human MIGL, but not in human OLG (Figures 8A, 8B, and S8B). However, the corresponding mouse loci at the promoter of *H2-ab1* presented open chromatin in EAE-OLG and MiGl (Figures 8A and 8B; Table S8). In addition, a putative enhancer region of *H2-eb1* also presented CA in EAE-OLG, but neither in human OLG nor in MIGL (SNP ID rs67476479_CA at *HLA-DRB1*). The outside variant SNP rs6498169 (Factor et al., 2020), located in between the loci of the master MHC-II regulator *Ciita* and *Socs1*, a major regulator of inflammation, also presented CA specifically in EAE-OPC and EAE-MOL, but not in Ctr-OLG or MiGl (Figure S8E; Table S8). *Socs1* is defined as Type1b in MOL5/6 (Table S2). The non-MHC SNP rs7975763 within the *Pitpnm2* locus was overlapping mainly with CA in OPC-EAE (Figure S8E; Table S8). This suggests that the CA of these SNPs might increase only in MS patients and not in healthy individuals.

Our data suggest that these SNPs might be involved in the modulation of regulatory regions in OLG, leading to altered transcriptional output and ultimately to altered function of OLG in the context of MS. We investigated potential TF-binding sites at specific SNPs, by intersecting our results with predictions of SNPs with potential effect on TF binding changes (SNP2TFBS) (Kumar et al., 2017). We found that the binding of TFs as EGR1, SP1, ETS1 and SOX10 might be affected in OLG in EAE or in OPCs treated with IFN-γ (Table S8). Moreover, scanning the region around MS-associated SNPs, 500 bp upstream and downstream, and filtering for expression (scRNA-seq) led to the identification of several other TFs whose binding might be potentially affected by OLG cell state transitions in the context of EAE (Table S8).

Genes associated with MS susceptibility SNPs can be modulated in OPCs by IFN-γ

We investigated whether IFN-γ treatment would affect the chromatin landscape and transcription of genes associated with MS SNPs and outside variants. The SNP located at *H2-Ab1* (SNP AADQb1-position_-5_L at *HLA-DBQ1*) overlaps with high levels of H3K27me3 in OPCs (Figure S8F; Table S8). Upon IFN-γ treatment, H3K27me3 is removed at the SNP locus, together with an increase in H3K27ac, H3K4me3, CTCF binding, and interactions with a *H2-ab1* enhancer (Figure S8F). IFN-γ also led to altered promoter-enhancer interactions in OPCs (Table S6).

Many SNPs are in intergenic regions; therefore, we investigated whether any SNP not assigned to a gene interacts with genes with altered expression upon IFN-γ treatment. SNP rs7191700 downstream of the *SOCS1* locus did not overlap

Figure 8. Overlap between CA peaks and MS susceptibility SNPs in OLG and activation of associated genes in mouse OPCs by IFN-γ

(A) Dot plot depicting overlap of CA peaks in specific cell types from human healthy individuals hg19 single-cell multi-ome and scATAC-seq cell types from Ctr and EAE mice, with individual MS susceptibility MHC, non-MHC, and suggestive SNPs (International Multiple Sclerosis Genetics Consortium, 2019) and outside variants (Factor et al., 2020).

(B and C) SNP coordinates for six MS SNPs in the hg38 human genome reference are shown with corresponding CA regions derived from merged scATAC-seq populations from the adult brain single-cell multi-ome. (B) Corresponding locations are shown in the mouse mm10 genome reference with IGV tracks of CA in 50 randomly selected individual cells from scATAC-seq from EAE and CFA-Ctr mice. Red boxes show scATAC-seq peaks from mouse overlapping with SNP location. (C) Corresponding locations are shown in the mouse mm10 genome reference with IGV tracks for bulk ATAC-seq, RNA-seq, ABC model, and Cut&Run with antibodies against H3K27me3, H3K27ac, CTCF, H3K4me3 in IFN-γ-treated and Ctr-OPCs. Red boxes show CA peaks from mouse overlapping with SNP location and their ABC connections. Cell acronyms as in Figure 7.

with CA in Ctr-OPCs or human OLG and MIGL (Figures 8A, 8C, and S8B). However, upon IFN-γ treatment, connections from the SNP locus were formed to the *Socs1* promoter together with increased CA and expression (Figure 8C; Table S8).

The SNP rs2248359-C, neighboring the *CYP24A1* gene encoding a protein involved in vitamin D3 degradation, has been suggested to link MS risk and vitamin D metabolism in the brain (Ramasamy et al., 2014). We found that a regulatory region overlapping with another SNP (rs2248137) at the *Cyp24a1* promoter presented increased interactions with the *Bcas1* promoter upon IFN-γ treatment (Figure 8C; Table S8). *Bcas1* is important for early myelination (Fard et al., 2017; Ishimoto et al., 2017), and its expression is decreased upon IFN-γ treatment (Figure 8C). The interacting SNP region overlaps with high levels of H3K27me3, which might explain decreased expression of *Bcas1* upon IFN-γ treatment (Figure 8C). Thus, our data suggest that SNPs at the *CYP24A1* locus might not only be involved in vitamin D metabolism in MS, but also in OL differentiation and possibly myelination. Moreover, regulatory regions overlapping with MS susceptibility SNPs control the expression of neighboring genes and are amenable to modulation by IFN-γ in OPCs, most likely affecting both the transition of OPCs to immune and differentiated states.

DISCUSSION

In this study, we find that OLG are primed at the chromatin level to be able to activate immune gene programs in the context of disease. This primed immune chromatin state is a cellular state in which immune genes have CA in homeostatic conditions at their promoter regulatory regions but have low or no expression. This state might also constitute epigenetic memory from past biological processes the cell has endured.

Type1b genes involved in antigen presentation are already accessible in OLG (Figures 2A, 3B, and S2F) but have a further increase in CA when the cells are exposed to an inflammatory environment. Type2a genes involved in immune processes in MOL1/2 and primary OPCs have high CA that does not change when the cell is exposed to an inflammatory environment. The observed differences between scATAC-seq and scRNA-seq indicate that OLG present open chromatin at (1) genes regulating biological processes that are already transcriptionally active and (2) genes that might be transcriptionally activated upon a given stimulus. Exposure to an inflammatory environment such as EAE and IFN-γ treatment might not significantly change the CA status but lead to gene expression by other chromatin-related mechanisms such as resolution of the bivalency for H3K4me3 and H3K27me3, rearrangement of CTCF binding, and remodeling of promoter-enhancer interactions. While our data support the role of distal enhancers in establishing cell identities (Heintzman et al., 2009; Thurman et al., 2012), they also highlight the importance of promoters not only in this process, but more importantly, in the transition between functional and disease-specific cell states within the same cell identity.

The expression of immune genes in OPCs and MOLs in the context of MS adds new roles to the functional portfolio of OLG, which might also occur in Alzheimer's disease (AD) (Zhou et al., 2020) and in aging (de la Fuente et al., 2020; Dulken et al., 2019; Spitzer et al., 2019; Ximerakis et al., 2019). Primed immune epigenomic programs might also be present in other cell types that unexpectedly activate immune transcriptional programs, such as structural cells (Krausgruber et al., 2020) and intestinal stem cells (Biton et al., 2018), suggesting a second line of immunological responses mediated by non-specialized immune cells. Our data indicate that most cell types in the CNS present immune chromatin priming, including neurons, consistent with the induction of MHC-I expression in neurons expressing ApoE in the context of AD (Zalocusky et al., 2021).

SNPs in MS are in most cases located nearby genes involved in immune regulation (International Multiple Sclerosis Genetics Consortium, 2019). Thus, MS susceptibility has been linked to immune cells within the CNS or in the periphery. Our data indicate that a subset of SNPs present in MS patients is in regulatory regions with CA in OLG in homeostatic conditions in mouse and in healthy individuals in human, or exhibit increased CA in OLG in the EAE mouse model of MS. Some of the genes that are associated with these regulatory regions have altered expression in EAE (Falcão et al., 2019). Recent findings suggest that SNPs located in regulatory regions of genes involved in transcriptional elongation might be involved in dysregulation of OL differentiation in the context of MS (Factor et al., 2020). Thus, susceptibility for MS might lead to disease onset, progression, or remission by the activation of abnormal immune and non-immune transcriptional programs not only in immune cells but also in OLG, which therefore constitute novel targets for immunological-based therapies for MS.

STAR★METHODS

Detailed methods are provided in the online version of this paper and include the following:

- KEY RESOURCES TABLE
- RESOURCE AVAILABILITY
  - Lead contact
  - Materials availability
  - Data and code availability
- EXPERIMENTAL MODEL AND SUBJECT DETAILS
  - Animals
  - Experimental autoimmune encephalomyelitis (EAE)
- METHOD DETAILS
  - Tissue dissociation for single-cell ATAC-seq experiment
  - Single-cell ATAC-seq (10x Genomics)
  - Plate-based single-cell ATAC-seq (Pi-ATAC)
  - Single-cell multi-omics (10x Genomics)–mouse
  - Single-cell multi-omics (10x Genomics)–human
  - Tissue dissociation for primary OPC cultures
  - Primary OPC culture
  - EZH2 inhibition
  - Transcription factor knockdowns
  - Bulk ATAC-seq
  - RNA extraction, cDNA synthesis and qRT-PCR
  - RNA-seq
  - Cut&Run
  - CUT&Tag for STAT1

- ○ H3K27ac-HiChIP
- ○ Western Blot
- ○ scATAC-seq 10X Genomics preprocessing
- ○ scATAC-seq Pi-ATAC preprocessing
- ○ Normalization and clustering of scATAC-seq
- ○ Gene activity scores and integration with scRNA-seq data
- ○ Differentially accessible peaks
- ○ Gene ontology analysis
- ○ scATAC peaks annotations
- ○ MS-associated SNPs
- ○ MS GWAS loci overlapping
- ○ TF motifs differential accessibility
- ○ Bulk ATAC-seq alignment and peak calling
- ○ Bulk RNA-seq alignment and differential gene expression
- ○ CUT&RUN alignment and processing
- ○ HiChIP
- ○ ABC model
- ○ Correlation between expression and chromatin accessibility
- ○ Gene set types definition for single-cell and bulk samples
- ○ Enhancer to promoter interactions from scATAC-seq
- ○ CUT&RUN peaks intersection
- ○ Number of predicted interactions in Ctr-OPCs and IFNγ treated OPCs
- ○ Single-cell multi-omics (mouse) data processing
- ○ Single-cell multiomics (human) data processing
- ○ Bulk CUT&TAG processing and peak calling
- ○ Bulk RNA-seq KD alignment and processing
- ○ GSEA in single-cell ATAC-seq
- ○ Motifs analysis around MS-associated SNP locations

SUPPLEMENTAL INFORMATION

Supplemental information can be found online at https://doi.org/10.1016/j.neuron.2021.12.034.

ACKNOWLEDGMENTS

We would like to thank Tony Jimenez-Beristain, Jaromir Mikes and the Biomedicum FACS facility, the Single Cell Genomics Facility, Karolinska Institute Protein Science facility, Elina Parvisto, Katarina Ericsson, Kristoffer Tenebro Berglund, and Johanna Hornstrand at the Comparative Medicine Biomedicum facility (KI) and its management for assistance in experiments; Matthew L. Speir at the University of California Santa Cruz Genomics Institute for setting up the UCSC Cell Browser and UCSC Genome Browser resources, Steve Henikoff for protein A-Tn5 and protein A-MNAse, and Sten Linnarsson's lab for additional support. The graphical abstract was generated using BioRender. The authors acknowledge support from the National Genomics Infrastructure in Stockholm funded by Science for Life Laboratory, the Knut and Alice Wallenberg Foundation, and the Swedish Research Council for assistance with massively parallel sequencing and access to the UPPMAX computational infrastructure. The computations/data handling were enabled by resources provided by the Swedish National Infrastructure for Computing (SNIC) at the Uppsala Multidisciplinary Center for Advanced Computational Science, partially funded by the Swedish Research Council through grant agreement no. 2018-05973. Tissue samples and associated clinical and neuropathological data were supplied by the Multiple Sclerosis Society Tissue Bank, funded by the Multiple Sclerosis Society of Great Britain and Northern Ireland, registered charity 207495. Grant support came from the following sources: M.R.J., National Institute for Health Research-Medical Research Council (MR/S02638X/1) and National Institute for Health Research Imperial Biomedical Research Centre. H.Y.C., NIH RM1-HG007735 and Howard Hughes Medical Institute; X.C., Swedish Research Council (VR-2017-02074); M.R.C., National Institute of Aging (K99AG059918); T.J.M., NIH P50 NS062684; M.R.C. and T.J.M., National Institute of Aging (U01AG072573); A.M.F., European Committee for Treatment and Research in Multiple Sclerosis (ECTRIMS), "La Caixa" Foundation (ID 100010434) with the reference LCF/BQ/PI19/11690005, and Swedish Research Council (grant no. 2019-02030); E.A., European Union, Horizon 2020, Marie-Sklodowska Curie Actions, and grant SOLO, number 794689; C.Z., China Scholarship Council (CSC) scholarship 201906170058; M.B., Vinnova Seal of Excellence Marie-Sklodowska Curie Actions grant RNA-centric view on Oligodendrocyte lineage development (RODent); L.K., ECTRIMS; G.C.-B.'s research group-Swedish Research Council (grant 2015-03558; 2019-01360); the European Union (FP7/Marie Curie Integration EPIOPC and Horizon, 2020 Research and Innovation Program/European Research Council Consolidator Grant EPIScOPE, 681893), Swedish Brain Foundation (FO2017-0075; FO2018-0162), Ming Wai Lau Centre for Reparative Medicine, Swedish Cancer Society (Cancerfonden; CAN2016/555: 190394 Pj), Knut and Alice Wallenberg Foundation (grants 2019-0107; 2019-0089), Swedish Society for Medical Research (SSMF, grant JUB2019), Olav Thon Foundation (grant 20-4543), F. Hoffmann-La Roche, and Karolinska Institutet.

AUTHOR CONTRIBUTIONS

M.M., E.A., and G.C.-B. conceived and designed the project, interpreted data, and wrote the manuscript. M.M., M.K., C.A.v.T., A.H., C.Z., L.K., D.C., M.B., and A.M.F. performed experiments. E.A. performed computational analyses, M.K. performed ABC analysis. M.R.J. and D.M. provided human tissues for the multi-ome experiments. M.R.C., T.J.M., and H.Y.C. performed and provided human scATAC-seq data. X.C. and H.Y.C. provided valuable advice and input for Pi-ATAC experiments. G.C.-B. supervised the project. All the authors critically reviewed the manuscript and approved the final version.

DECLARATION OF INTERESTS

H.Y.C. is a co-founder of Accent Therapeutics and Boundless Bio and an advisor to 10x Genomics, Arsenal Biosciences, and Spring Discovery. D.M. and D.C. are employees at Roche Pharma Research and Early Development. The other authors declare no competing interests.

Received: January 14, 2021
Revised: November 5, 2021
Accepted: December 27, 2021
Published: January 28, 2022

REFERENCES

Absinta, M., Maric, D., Gharagozloo, M., Garton, T., Smith, M.D., Jin, J., Fitzgerald, K.C., Song, A., Liu, P., Lin, J.P., et al. (2021). A lymphocyte-microglia-astrocyte axis in chronic active multiple sclerosis. Nature *597*, 709–714.

Althammer, S., González-Vallinas, J., Ballaré, C., Beato, M., and Eyras, E. (2011). Pyicos: a versatile toolkit for the analysis of high-throughput sequencing data. Bioinformatics *27*, 3333–3340.

Bernstein, B.E., Mikkelsen, T.S., Xie, X., Kamal, M., Huebert, D.J., Cuff, J., Fry, B., Meissner, A., Wernig, M., Plath, K., et al. (2006). A bivalent chromatin structure marks key developmental genes in embryonic stem cells. Cell *125*, 315–326.

Bindea, G., Mlecnik, B., Hackl, H., Charoentong, P., Tosolini, M., Kirilovsky, A., Fridman, W.-H., Pagès, F., Trajanoski, Z., and Galon, J. (2009). ClueGO: a cytoscape plug-in to decipher functionally grouped gene ontology and pathway annotation networks. Bioinformatics *25*, 1091–1093.

Biton, M., Haber, A.L., Rogel, N., Burgin, G., Beyaz, S., Schnell, A., Ashenberg, O., Su, C.W., Smillie, C., Shekhar, K., et al. (2018). T helper cell cytokines

modulate intestinal stem cell renewal and differentiation. Cell *175*, 1307–1320.e22.

Borcherding, N., Vishwakarma, A., Voigt, A.P., Bellizzi, A., Kaplan, J., Nepple, K., Salem, A.K., Jenkins, R.W., Zakharia, Y., and Zhang, W. (2021). Mapping the immune environment in clear cell renal carcinoma by single-cell genomics. Commun. Biol. *4*, 122.

Buenrostro, J.D., Giresi, P.G., Zaba, L.C., Chang, H.Y., and Greenleaf, W.J. (2013). Transposition of native chromatin for fast and sensitive epigenomic profiling of open chromatin, DNA-binding proteins and nucleosome position. Nat. Methods *10*, 1213–1218.

Burr, M.L., Sparbier, C.E., Chan, K.L., Chan, Y.C., Kersbergen, A., Lam, E.Y.N., Azidis-Yates, E., Vassiliadis, D., Bell, C.C., Gilan, O., et al. (2019). An evolutionarily conserved function of Polycomb silences the MHC class I antigen presentation pathway and enables immune evasion in cancer. Cancer Cell *36*, 385–401.e8.

Butler, A., Hoffman, P., Smibert, P., Papalexi, E., and Satija, R. (2018). Integrating single-cell transcriptomic data across different conditions, technologies, and species. Nat. Biotechnol. *36*, 411–420.

Chen, X., Litzenburger, U.M., Wei, Y., Schep, A.N., LaGory, E.L., Choudhry, H., Giaccia, A.J., Greenleaf, W.J., and Chang, H.Y. (2018). Joint single-cell DNA accessibility and protein epitope profiling reveals environmental regulation of epigenomic heterogeneity. Nat. Commun. *9*, 4590.

Chew, L.J., King, W.C., Kennedy, A., and Gallo, V. (2005). Interferon-gamma inhibits cell cycle exit in differentiating oligodendrocyte progenitor cells. Glia *52*, 127–143.

Conway, J.R., Lex, A., and Gehlenborg, N. (2017). UpSetR: an R package for the visualization of intersecting sets and their properties. Bioinformatics *33*, 2938–2940.

Corces, M.R., Shcherbina, A., Kundu, S., Gloudemans, M.J., Frésard, L., Granja, J.M., Louie, B.H., Eulalio, T., Shams, S., Bagdatli, S.T., et al. (2020). Single-cell epigenomic analyses implicate candidate causal variants at inherited risk loci for Alzheimer's and Parkinson's diseases. Nat. Genet. *52*, 1158–1168.

de Boer, C.G., and Regev, A. (2018). BROCKMAN: deciphering variance in epigenomic regulators by k-mer factorization. BMC Bioinformatics *19*, 253.

De Jager, P.L., Jia, X., Wang, J., de Bakker, P.I., Ottoboni, L., Aggarwal, N.T., Piccio, L., Raychaudhuri, S., Tran, D., Aubin, C., et al. (2009). Meta-analysis of genome scans and replication identify CD6, IRF8 and TNFRSF1A as new multiple sclerosis susceptibility loci. Nat. Genet. *41*, 776–782.

de la Fuente, A.G., Queiroz, R.M.L., Ghosh, T., McMurran, C.E., Cubillos, J.F., Bergles, D.E., Fitzgerald, D.C., Jones, C.A., Lilley, K.S., Glover, C.P., et al. (2020). Changes in the oligodendrocyte progenitor cell proteome with ageing. Mol. Cell. Proteomics *19*, 1281–1302.

de Leeuw, C.A., Mooij, J.M., Heskes, T., and Posthuma, D. (2015). MAGMA: generalized gene-set analysis of GWAS data. PLoS Comput. Biol. *11*, e1004219.

Dobin, A., Davis, C.A., Schlesinger, F., Drenkow, J., Zaleski, C., Jha, S., Batut, P., Chaisson, M., and Gingeras, T.R. (2013). STAR: ultrafast universal RNA-seq aligner. Bioinformatics *29*, 15–21.

Dulken, B.W., Buckley, M.T., Navarro Negredo, P., Saligrama, N., Cayrol, R., Leeman, D.S., George, B.M., Boutet, S.C., Hebestreit, K., Pluvinage, J.V., et al. (2019). Single-cell analysis reveals T cell infiltration in old neurogenic niches. Nature *571*, 205–210.

Factor, D.C., Barbeau, A.M., Allan, K.C., Hu, L.R., Madhavan, M., Hoang, A.T., Hazel, K.E.A., Hall, P.A., Nisraiyya, S., Najm, F.J., et al. (2020). Cell type-specific intralocus interactions reveal oligodendrocyte mechanisms in MS. Cell *181*, 382–395.e21.

Falcão, A.M., van Bruggen, D., Marques, S., Meijer, M., Jäkel, S., Agirre, E., Samudyata, F., M., E., Vanichkina, D.P., Ffrench-Constant, C., et al. (2019). Disease-specific oligodendrocyte lineage cells arise in multiple sclerosis. Nat. Med. *24*, 1837–1844.

Fard, M.K., van der Meer, F., Sánchez, P., Cantuti-Castelvetri, L., Mandad, S., Jäkel, S., Fornasiero, E.F., Schmitt, S., Ehrlich, M., Starost, L., et al. (2017). BCAS1 expression defines a population of early myelinating oligodendrocytes in multiple sclerosis lesions. Sci. Transl. Med. *9*, eaam7816.

Fernández-Castañeda, A., Chappell, M.S., Rosen, D.A., Seki, S.M., Beiter, R.M., Johanson, D.M., Liskey, D., Farber, E., Onengut-Gumuscu, S., Overall, C.C., et al. (2020). The active contribution of OPCs to neuroinflammation is mediated by LRP1. Acta Neuropathol. *139*, 365–382.

Finucane, H.K., Reshef, Y.A., Anttila, V., Slowikowski, K., Gusev, A., Byrnes, A., Gazal, S., Loh, P.R., Lareau, C., Shoresh, N., et al. (2018). Heritability enrichment of specifically expressed genes identifies disease-relevant tissues and cell types. Nat. Genet. *50*, 621–629.

Franklin, R.J.M., and Ffrench-Constant, C. (2017). Regenerating CNS myelin—from mechanisms to experimental medicines. Nat. Rev. Neurosci. *18*, 753–769.

Fulco, C.P., Nasser, J., Jones, T.R., Munson, G., Bergman, D.T., Subramanian, V., Grossman, S.R., Anyoha, R., Doughty, B.R., Patwardhan, T.A., et al. (2019). Activity-by-contact model of enhancer-promoter regulation from thousands of CRISPR perturbations. Nat. Genet. *51*, 1664–1669.

Granja, J.M., Corces, M.R., Pierce, S.E., Bagdatli, S.T., Choudhry, H., Chang, H., and Greenleaf, W. (2020). ArchR: an integrative and scalable software package for single-cell chromatin accessibility analysis. bioRxiv, bioRxiv: 2020.04.28.066498.

Grant, Charles, E, Bailey, Timothy, L, Noble, William, S, et al. (2011). FIMO: scanning for occurrences of a given motif. Bioinformatics *27*, 1017–1018. https://doi.org/10.1093/bioinformatics/btr064.

Gu, Z., Eils, R., and Schlesner, M. (2016). Complex heatmaps reveal patterns and correlations in multidimensional genomic data. Bioinformatics *32*, 2847–2849.

Heintzman, N.D., Hon, G.C., Hawkins, R.D., Kheradpour, P., Stark, A., Harp, L.F., Ye, Z., Lee, L.K., Stuart, R.K., Ching, C.W., et al. (2009). Histone modifications at human enhancers reflect global cell-type-specific gene expression. Nature *459*, 108–112.

Heinz, S., Benner, C., Spann, N., Bertolino, E., Lin, Y.C., Laslo, P., Cheng, J.X., Murre, C., Singh, H., and Glass, C.K. (2010). Simple combinations of lineage-determining transcription factors prime *cis*-regulatory elements required for macrophage and B cell identities. Mol. Cell *38*, 576–589.

Hu, X., and Ivashkiv, L.B. (2009). Cross-regulation of signaling pathways by interferon-gamma: implications for immune responses and autoimmune diseases. Immunity *31*, 539–550.

Igarashi, K., Kurosaki, T., and Roychoudhuri, R. (2017). BACH transcription factors in innate and adaptive immunity. Nat. Rev. Immunol. *17*, 437–450.

International Multiple Sclerosis Genetics Consortium (2019). Multiple sclerosis genomic map implicates peripheral immune cells and microglia in susceptibility. Science *365*, eaav7188.

Ishimoto, T., Ninomiya, K., Inoue, R., Koike, M., Uchiyama, Y., and Mori, H. (2017). Mice lacking BCAS1, a novel myelin-associated protein, display hypomyelination, schizophrenia-like abnormal behaviors, and upregulation of inflammatory genes in the brain. Glia *65*, 727–739.

Jäkel, S., Agirre, E., Mendanha Falcão, A.M., van Bruggen, D., Lee, K.W., Knuesel, I., Malhotra, D., ffrench-Constant, C., Williams, A., and Castelo-Branco, G. (2019). Altered human oligodendrocyte heterogeneity in multiple sclerosis. Nature *566*, 543–547.

Kaya-Okur, H.S., Wu, S.J., Codomo, C.A., Pledger, E.S., Bryson, T.D., Henikoff, J.G., Ahmad, K., and Henikoff, S. (2019). CUT&Tag for efficient epigenomic profiling of small samples and single cells. Nat. Commun. *10*, 1930.

Kirby, L., Jin, J., Cardona, J.G., Smith, M.D., Martin, K.A., Wang, J., Strasburger, H., Herbst, L., Alexis, M., Karnell, J., et al. (2019). Oligodendrocyte precursor cells present antigen and are cytotoxic targets in inflammatory demyelination. Nat. Commun. *10*, 3887.

Korsunsky, I., Millard, N., Fan, J., Slowikowski, K., Zhang, F., Wei, K., Baglaenko, Y., Brenner, M., Loh, P., and Raychaudhuri, S. (2019). Fast, sensitive and accurate integration of single-cell data with Harmony. Nature methods, 1289–1296. https://doi.org/10.1038/s41592-019-0619-0.

CellPress
OPEN ACCESS

Krausgruber, T., Fortelny, N., Fife-Gernedl, V., Senekowitsch, M., Schuster, L.C., Lercher, A., Nemc, A., Schmidl, C., Rendeiro, A.F., Bergthaler, A., et al. (2020). Structural cells are key regulators of organ-specific immune responses. Nature *583*, 296–302.

Kumar, S., Ambrosini, G., and Bucher, P. (2017). SNP2TFBS—a database of regulatory SNPs affecting predicted transcription factor binding site affinity. Nucleic Acids Res. *45*, D139–D144.

Langmead, B., and Salzberg, S.L. (2012). Fast gapped-read alignment with Bowtie 2. Nat. Methods *9*, 357–359.

Li, H., Handsaker, B., Wysoker, A., Fennell, T., Ruan, J., Homer, N., Marth, G., Abecasis, G., and Durbin, R.; 1000 Genome Project Data Processing Subgroup (2009). The sequence alignment/map format and SAMtools. Bioinformatics *25*, 2078–2079.

Love, M.I., Huber, W., and Anders, S. (2014). Moderated estimation of fold change and dispersion for RNA-seq data with DESeq2. Genome Biol. *15*, 550.

Majumder, P., and Boss, J.M. (2010). CTCF controls expression and chromatin architecture of the human major histocompatibility complex class II locus. Mol. Cell. Biol. *30*, 4211–4223.

Majumder, P., Scharer, C.D., Choi, N.M., and Boss, J.M. (2014). B cell differentiation is associated with reprogramming the CCCTC binding factor-dependent chromatin architecture of the murine MHC class II locus. J. Immunol. *192*, 3925–3935.

Matsuoka, T., Ahlberg, P.E., Kessaris, N., Iannarelli, P., Dennehy, U., Richardson, W.D., McMahon, A.P., and Koentges, G. (2005). Neural crest origins of the neck and shoulder. Nature *436*, 347–355.

McCarthy, D.J., Chen, Y., and Smyth, G.K. (2012). Differential expression analysis of multifactor RNA-seq experiments with respect to biological variation. Nucleic Acids Res. *40*, 4288–4297.

McLean, C.Y., Bristor, D., Hiller, M., Clarke, S.L., Schaar, B.T., Lowe, C.B., Wenger, A.M., and Bejerano, G. (2010). GREAT improves functional interpretation of *cis*-regulatory regions. Nat. Biotechnol. *28*, 495–501.

Morgan, Martin, Falcon, Seth, and Gentleman, Robert (2021). GSEABase: Gene set enrichment data structures and methods. Bioconductor *R package version 1.56.0.*. https://doi.org/10.18129/B9.bioc.GSEABase.

Muhlethaler-Mottet, A., Otten, L.A., Steimle, V., and Mach, B. (1997). Expression of MHC class II molecules in different cellular and functional compartments is controlled by differential usage of multiple promoters of the transactivator CIITA. EMBO J. *16*, 2851–2860.

Mumbach, M.R., Rubin, A.J., Flynn, R.A., Dai, C., Khavari, P.A., Greenleaf, W.J., and Chang, H.Y. (2016). HiChIP: efficient and sensitive analysis of protein-directed genome architecture. Nat. Methods *13*, 919–922.

Nikcevich, K.M., Piskurich, J.F., Hellendall, R.P., Wang, Y., and Ting, J.P. (1999). Differential selectivity of CIITA promoter activation by IFN-gamma and IRF-1 in astrocytes and macrophages: CIITA promoter activation is not affected by TNF-alpha. J. Neuroimmunol. *99*, 195–204.

Patro, R., Duggal, G., Love, M.I., Irizarry, R.A., and Kingsford, C. (2017). Salmon provides fast and bias-aware quantification of transcript expression. Nat. Methods *14*, 417–419.

Picelli, S., Björklund, A.K., Reinius, B., Sagasser, S., Winberg, G., and Sandberg, R. (2014). Tn5 transposase and tagmentation procedures for massively scaled sequencing projects. Genome Res. *24*, 2033–2040.

Pliner, H.A., Packer, J.S., McFaline-Figueroa, J.L., Cusanovich, D.A., Daza, R.M., Aghamirzaie, D., Srivatsan, S., Qiu, X., Jackson, D., Minkina, A., et al. (2018). Cicero predicts cis-regulatory DNA interactions from single-cell chromatin accessibility data. Mol. Cell *71*, 858–871.e8.

Quinlan, A.R., and Hall, I.M. (2010). BEDTools: a flexible suite of utilities for comparing genomic features. Bioinformatics *26*, 841–842.

Ramasamy, A., Trabzuni, D., Forabosco, P., Smith, C., Walker, R., Dillman, A., Sveinbjornsdottir, S.; North American Brain Expression Consortium (NABEC); UK Brain Expression Consortium (UKBEC), and Hardy, J., et al. (2014). Genetic evidence for a pathogenic role for the vitamin D3 metabolizing enzyme CYP24A1 in multiple sclerosis. Mult. Scler. Relat. Disord. *3*, 211–219.

Ramírez, F., Dündar, F., Diehl, S., Grüning, B.A., and Manke, T. (2014). deepTools: a flexible platform for exploring deep-sequencing data. Nucleic Acids Res. *42*, W187–W191.

Ramírez, F., Ryan, D.P., Grüning, B., Bhardwaj, V., Kilpert, F., Richter, A.S., Heyne, S., Dündar, F., and Manke, T. (2016). deepTools2: a next generation web server for deep-sequencing data analysis. Nucleic Acids Res. *44*, W160–W165.

Robinson, M.D., McCarthy, D.J., and Smyth, G.K. (2010). edgeR: a Bioconductor package for differential expression analysis of digital gene expression data. Bioinformatics *26*, 139–140.

Robinson, James T., Turner, Douglass, Durand, Neva C., Thorvaldsdóttir, Helga, Mesirov, Jill P., and Aiden, Erez Lieberman (2018). Juicebox.js provides a cloud-based visualization system for Hi-C data. Cell Syst. *6* (2). https://doi.org/10.1016/j.cels.2018.01.001.

Schep, A.N., Wu, B., Buenrostro, J.D., and Greenleaf, W.J. (2017). chromVAR: inferring transcription-factor-associated accessibility from single-cell epigenomic data. Nat. Methods *14*, 975–978.

Schmid, M., Durussel, T., and Laemmli, U.K. (2004). ChIC and ChEC; genomic mapping of chromatin proteins. Mol. Cell *16*, 147–157.

Schmidt, E.M., Zhang, J., Zhou, W., Chen, J., Mohlke, K.L., Chen, Y.E., and Willer, C.J. (2015). GREGOR: evaluating global enrichment of trait-associated variants in epigenomic features using a systematic, data-driven approach. Bioinformatics *31*, 2601–2606.

Sen, M.K., Almuslehi, M.S.M., Shortland, P.J., Coorssen, J.R., and Mahns, D.A. (2020). Revisiting the pathoetiology of multiple sclerosis: has the tail been wagging the mouse? Front. Immunol. *11*, 572186.

Skene, P.J., and Henikoff, S. (2017). An efficient targeted nuclease strategy for high-resolution mapping of DNA binding sites. Elife *6*, e21856.

Smoot, M.E., Ono, K., Ruscheinski, J., Wang, P.L., and Ideker, T. (2011). Cytoscape 2.8: new features for data integration and network visualization. Bioinformatics *27*, 431–432.

Sousa, V.H., Miyoshi, G., Hjerling-Leffler, J., Karayannis, T., and Fishell, G. (2009). Characterization of Nkx6-2-derived neocortical interneuron lineages. Cereb. Cortex *19* (*Suppl. 1*), i1–i10.

Spitzer, S.O., Sitnikov, S., Kamen, Y., Evans, K.A., Kronenberg-Versteeg, D., Dietmann, S., de Faria, O., Jr., Agathou, S., and Káradóttir, R.T. (2019). Oligodendrocyte progenitor cells become regionally diverse and heterogeneous with age. Neuron *101*, 459–471.e5.

Stik, G., Vidal, E., Barrero, M., Cuartero, S., Vila-Casadesús, M., Mendieta-Esteban, J., Tian, T.V., Choi, J., Berenguer, C., Abad, A., et al. (2020). CTCF is dispensable for immune cell transdifferentiation but facilitates an acute inflammatory response. Nat. Genet. *52*, 655–661.

Stolt, C.C., Lommes, P., Friedrich, R.P., and Wegner, M. (2004). Transcription factors Sox8 and Sox10 perform non-equivalent roles during oligodendrocyte development despite functional redundancy. Development *131*, 2349–2358.

Stuart, T., Srivastava, A., Lareau, C., and Satija, R. (2020). Multimodal single-cell chromatin analysis with Signac. bioRxiv, bioRxiv:2020.11.09.373613.

Thurman, R.E., Rynes, E., Humbert, R., Vierstra, J., Maurano, M.T., Haugen, E., Sheffield, N.C., Stergachis, A.B., Wang, H., Vernot, B., et al. (2012). The accessible chromatin landscape of the human genome. Nature *489*, 75–82.

Tintore, M., Vidal-Jordana, A., and Sastre-Garriga, J. (2019). Treatment of multiple sclerosis - success from bench to bedside. Nat. Rev. Neurol. *15*, 53–58.

Wang, Q., Gu, L., Adey, A., Radlwimmer, B., Wang, W., Hovestadt, V., Bähr, M., Wolf, S., Shendure, J., Eils, R., et al. (2013). Tagmentation-based whole-genome bisulfite sequencing. Nat. Protoc. *8*, 2022–2032.

Wang, Y., Ren, Z., Tao, D., Tilwalli, S., Goswami, R., and Balabanov, R. (2010). STAT1/IRF-1 signaling pathway mediates the injurious effect of interferon-gamma on oligodendrocyte progenitor cells. Glia *58*, 195–208.

Weirauch, M.T., Yang, A., Albu, M., Cote, A.G., Montenegro-Montero, A., Drewe, P., Najafabadi, H.S., Lambert, S.A., Mann, I., Cook, K., et al. (2014).

Determination and inference of eukaryotic transcription factor sequence specificity. Cell *158*, 1431–1443.

Ximerakis, M., Lipnick, S.L., Innes, B.T., Simmons, S.K., Adiconis, X., Dionne, D., Mayweather, B.A., Nguyen, L., Niziolek, Z., Ozek, C., et al. (2019). Single-cell transcriptomic profiling of the aging mouse brain. Nat. Neurosci. *22*, 1696–1708.

Yeung, M.S.Y., Djelloul, M., Steiner, E., Bernard, S., Salehpour, M., Possnert, G., Brundin, L., and Frisén, J. (2019). Dynamics of oligodendrocyte generation in multiple sclerosis. Nature *566*, 538–542.

Zalocusky, K.A., Najm, R., Taubes, A.L., Hao, Y., Yoon, S.Y., Koutsodendris, N., Nelson, M.R., Rao, A., Bennett, D.A., Bant, J., et al. (2021). Neuronal ApoE upregulates MHC-I expression to drive selective neurodegeneration in Alzheimer's disease. Nat. Neurosci. *24*, 786–798.

Zeisel, A., Yitzhaky, A., Bossel Ben-Moshe, N., and Domany, E. (2013). An accessible database for mouse and human whole transcriptome qPCR primers. Bioinformatics *29*, 1355–1356.

Zhang, Y., Liu, T., Meyer, C.A., Eeckhoute, J., Johnson, D.S., Bernstein, B.E., Nusbaum, C., Myers, R.M., Brown, M., Li, W., et al. (2008). Model-based analysis of ChIP-seq (MACS). Genome Biol. *9*, R137.

Zhou, Y., Song, W.M., Andhey, P.S., Swain, A., Levy, T., Miller, K.R., Poliani, P.L., Cominelli, M., Grover, S., Gilfillan, S., et al. (2020). Human and mouse single-nucleus transcriptomics reveal TREM2-dependent and TREM2-independent cellular responses in Alzheimer's disease. Nat. Med. *26*, 131–142.

Zhu, Q., Liu, N., Orkin, S.H., and Yuan, G.C. (2019). CUT&RUNTools: a flexible pipeline for CUT&RUN processing and footprint analysis. Genome Biol. *20*, 192.

CellPress
OPEN ACCESS

STAR★METHODS

KEY RESOURCES TABLE

REAGENT or RESOURCE	SOURCE	IDENTIFIER
**Antibodies**		
H3K27me3	Cell Signaling 9733S; rabbit	RRID:AB_2616029
H3K4me3	Diagenode C15410003-50; rabbit	RRID:AB_2616052
H3K27ac	Abcam ab177178; rabbit	RRID:AB_2828007
CTCF	Cell Signaling 3418S; rabbit	RRID:AB_2086791
GAPDH	rabbit monoclonal, 5174S, Cell Signaling	RRID:AB_10622025
STAT1	Cell Signaling 14994S; rabbit, monoclonal	RRID:AB_2737027
secondary antibody anti-rabbit	A6667, Sigma	RRID:AB_258307
GFP	FITC conjugated, Ab6662, Abcam	RRID:AB_305635
**Chemicals, peptides, and recombinant proteins**		
EZH2 inhibitor EPZ011989	Selleckchem	S7805
Protein A-MNase	Schmid et al., 2004	N/A
**Critical commercial assays**		
emulsion of MOG35-55 peptide in complete Freud's adjuvant	Hooke laboratories	EK-2110
adult brain dissociation kit	Miltenyi	130-107-677
neural tissue dissociation kit	Miltenyi	130-092-628
Cd140a microbead kit	Miltenyi	130-101-547
Chromium Single Cell ATAC v1 Chemistry	10x Genomics	N/A
Chromium Next GEM Single Cell Multiome ATAC + Gene Expression v1	10x Genomics	N/A
**Deposited data**		
Raw sequencing data (scATAC-Seq, CUT&RUN, HiChIP, RNA-Seq, single cell multiome)	This paper	GEO: GSE166179, EGA: EGAS00001005911
scRNA-Seq EAE mouse model	Falcão et al., 2019	GEO: GSE113973
Human brain snATAC-Seq	Corces et al., 2020	GEO: GSE147672
**Experimental models: Organisms/strains**		
Mouse: RCE: loxP	Jens Herjling-Lefler, Karolinska Institutet, Gord Fishell, Harvard Medical School	Gt(ROSA)26Sor$^{tm1.1(CAG-EGFP)Fsh}$ in CD1 background; RRID:MMRRC_032037-JAX
Mouse Sox10:Cre	The Jackson Laboratory	025807
**Oligonucleotides**		
Ubc qRT-PCR primers<br>5'-agg tca aac agg aag aca gac gta-3'<br>5'-tca cac cca aga aca agc aca-3'	This paper	N/A
b-Act qRT-PCR primers<br>5'-ctc tgg ctc cta gca cca tga aga-3'<br>5'-gta aaa cgc agc tca gta aca gtc cg-3'	This paper	N/A
Nlrc5 qRT-PCR primers<br>5'-ccg tgg tac tca cat ttg cc-3'<br>5'-cct tcg aga tct ctg gga ca-3'	This paper	N/A
Psmb8 qRT-PCR primers<br>5'-cct tac ctg ctt ggc acc at-3'<br>5'-atg ctg cag aca cgg aga tg-3'	This paper	N/A
H2-q7 qRT-PCR primers<br>5'-ata cct gca gct cgg gaa g-3'<br>5'-agc acc ata aga cct ggg gt-3'	This paper	N/A

(*Continued on next page*)

Continued

REAGENT or RESOURCE	SOURCE	IDENTIFIER
Ciita qRT-PCR primers<br>5'-ctg gca cag gtc tct cca gt-3'<br>5'-tac tga ggc tgc ttg aag gg-3'	This paper	N/A
H2-aa qRT-PCR primers<br>5'-gct cag cct ctg tgg agg t-3'<br>5'-tgt act ggc caa tgt ctc ca-3'	This paper	N/A
H2-ab1 qRT-PCR primers<br>5'-gca gac aca act acg agg gg-3'<br>5'-agt gtt gtg gtg gtt gag gg-3'	This paper	N/A
Cd74 qRT-PCR primers<br>5'-ctg gat gaa gca gtg gct ct-3'<br>5'-ccc agg cca gaa gat agg tc-3'	This paper	N/A
Stat1 qRT-PCR primers<br>5'-aac atg ctg gtg aca gag cc-3'<br>5'-tgc caa ctc aac acc tct ga-3'	This paper	N/A
Bach1 qRT-PCR primers<br>5'-tcg aga atc gta ggc cag ac-3'<br>5'-tgg cag tgt agg caa act ga-3'	This paper	N/A
Hmox1 qRT-PCR primers<br>5'-gcc gag aat gct gag ttc at-3'<br>5'-tcc agg gcc gtg tag ata tg-3'	This paper	N/A
SiGENOME SMARTpool siRNA *Ctr*<br>5'-uag cga cua aac aca uca a-3'<br>5'-uaa ggc uau gaa gag aua c-3'<br>5'-aug uau ugg ccu gua uua g-3'<br>5'-aug aac gug aau ugc uca a-3'	Dharmacon	D-001206-13-05
SiGENOME SMARTpool siRNA *Stat1*<br>5'-gga cgu ucc ugc uua gau u-3'<br>5'-aca cug uga ugu uag aua a-3'<br>5'-gca uag agc agg aaa uca a-3'<br>5'-cca ucg agc uca cuc aga a-3'	Dharmacon	M-058881-02-0005
SiGENOME SMARTpool siRNA *Bach1*<br>5'-gca gga gcc uug ccc gua u-3'<br>5'- gag ugu ccc ugg uug ggu a-3'<br>5'- aaa cua cga uua ugu cuc g-3'<br>5'- cga cug ccc gcu uuc cuu u-3'	Dharmacon	M-042956-01-0005
**Software and algorithms**		
Cell Ranger ATAC (version 1.2.0)	10x Genomics	https://support.10xgenomics.com/single-cell-atac/software/pipelines/1.2/what-is-cell-ranger-atac
Cell Ranger ARC v.2.0	10x Genomics	https://support.10xgenomics.com/single-cell-multiome-atac-gex/software/pipelines/latest/installation
DEseq2	Love et al., 2014	https://bioconductor.org/packages/release/bioc/html/DESeq2.html
EdgeR	Robinson et al., 2010	https://bioconductor.org/packages/release/bioc/html/edgeR.html
DeeptTools	Ramírez et al., 2016	https://deeptools.readthedocs.io/en/develop/content/installation.html
ComplexHeatmap	Gu et al., 2016	https://github.com/jokergoo/ComplexHeatmap
EnhancedVolcano	Blighe et al., 2018. "EnhancedVolcano: Publication-ready volcano plots with enhanced colouring and labeling."	https://github.com/kevinblighe/EnhancedVolcano
Bedtools	Quinlan and Hall, 2010	https://bedtools.readthedocs.io/en/latest/

(*Continued on next page*)

Continued

REAGENT or RESOURCE	SOURCE	IDENTIFIER
Pycios	Althammer et al., 2011	http://regulatorygenomics.upf.edu/Software/Pycioteo/pyicos.html
UpsetR	Conway et al., 2017	https://github.com/hms-dbmi/UpSetR
Signac	Stuart et al., 2020	https://satijalab.org/signac/.
De Bore pipeline	de Boer and Regev, 2018	https://carldeboer.github.io/brockman.html
Salmon	Patro et al., 2017	https://salmon.readthedocs.io/en/latest/index.html
Bowtie2	(Langmead and Salzberg, 2012)	http://bowtie-bio.sourceforge.net/bowtie2/index.shtml
MACS2	(Zhang et al., 2008)	https://github.com/macs3-project/MACS
chromVAR	(Schep et al., 2017)	https://greenleaflab.github.io/chromVAR/articles/Introduction.html
ClueGO (version 2.5.5)	(Bindea et al., 2009)	https://apps.cytoscape.org/apps/cluego
Cytoscape (version 3.7.2)	(Smoot et al., 2011)	https://cytoscape.org/
HOMER v4.11	(Heinz et al., 2010)	http://homer.ucsd.edu/homer/index.html
Cell type specific (CTS) LD score regression	(Finucane et al., 2018)	https://github.com/bulik/ldsc/wiki/Cell-type-specific-analyses
MAGMA v1.06	(de Leeuw et al., 2015)	https://ctg.cncr.nl/software/magma
GREGOR	(Schmidt et al., 2015)	http://csg.sph.umich.edu/GREGOR/
Seurat	(Butler et al., 2018)	https://github.com/satijalab/seurat
SAMtools	(Li et al., 2009)	http://www.htslib.org/
MarkDuplicates	Picard - latest version 1.126	http://broadinstitute.github.io/picard/
STAR 2.7	(Dobin et al., 2013)	https://github.com/alexdobin/STAR
Juicebox	(Robinson et al., 2018)	https://www.aidenlab.org/juicebox/
ABC model	(Fulco et al., 2019)	https://github.com/broadinstitute/ABC-Enhancer-Gene-Prediction
ArchR	(Granja et al., 2020)	https://www.archrproject.com
CTS LD score regression	(Finucane et al., 2018)	https://github.com/bulik/ldsc
CUT&RUNTools	(Zhu et al., 2019)	https://bitbucket.org/qzhudfci/cutruntools/
Other		
scATAC-Seq data webresources (also available at https://ki.se/en/mbb/oligointernode)	This paper	https://castelobranco.shinyapps.io/SCATAC10X_2020/. http://cells.ucsc.edu/?ds=olg-eae-ms
Sc-multiome data webresources (also available at https://ki.se/en/mbb/oligointernode)	This paper	https://castelobranco.shinyapps.io/eae_multi_act https://castelobranco.shinyapps.io/eae_multi_rna https://castelobranco.shinyapps.io/hs_ctr_multi_act https://castelobranco.shinyapps.io/hs_ctr_multi_rna
Genome tracks for all datasets	This paper	GEO: GSE166179
Code availability	This paper	DOI 10.5281/zenodo.5781403

RESOURCE AVAILABILITY

Lead contact

Further information and requests for resources and reagents should be directed to and will be fulfilled by the lead contact, Gonçalo Castelo-Branco (goncalo.castelo-branco@ki.se).

Materials availability

The study did not generate new unique reagents.

Data and code availability

IGV sessions links for hg19, hg39 and mm10 are available at https://github.com/Castelo-Branco-lab/Meijer_Agirre_scATACEAE_2020., alongside code, which is also available at https://doi.org/10.5281/zenodo.5781403. The scATAC-seq dataset can be explored at the web resources listed at the key resources table, and available at https://ki.se/en/mbb/oligointernode and http://cells.ucsc.edu/?ds=olg-eae-ms. All the raw and processed data generated in this work are available through accession SuperSeries GEO: GSE166179. In the case of human samples generated for this study raw data can be accessed through EGA: EGAS00001005911 accession and processed data files at GEO: GSE166179 . Human scATAC-seq processed control samples were retrieved from GEO: GSE147672 accession.

EXPERIMENTAL MODEL AND SUBJECT DETAILS

Animals

The mouse line used in this study was generated by crossing Sox10:Cre animals (Matsuoka et al., 2005) (The Jackson Laboratory mouse strain 025807) on a C57BL/6j genetic background with RCE:loxP (EGFP) animals (Sousa et al., 2009) (The Jackson Laboratory mouse strain 32037-JAX) on a C57BL/6xCD1 mixed genetic background. Females with a hemizygous Cre allele were mated with males lacking the Cre allele, whereas the reporter allele was kept in hemizygosity or homozygosity in both females and males. In the resulting Sox10:Cre-RCE:LoxP (EGFP) animals the entire OL lineage was labeled with EGFP. Breeding males containing a hemizygous Cre allele, in combination with the reporter allele, with non-Cre carrier females resulted in offspring where all cells were labeled with EGFP and was therefore avoided. For primary cell culture, animals of both sexes were sacrificed at P4-P6. For EAE experiments, males between the ages of 9 and 13 weeks were used for immunization and sacrificed 10–17 days after, at the peak of the disease.

All animals were free from the most common mouse viral pathogens, ectoparasites, endoparasites and mouse bacterial pathogens harbored in research animals. The battery of screened infective agents met the standard health profile established in Karolinska Institutet animal housing facilities. Mice were kept with the following light/dark cycle: dawn 6:00–7:00, daylight 7:00–18:00, dusk 18:00–19:00, and night 19:00–6:00; they were housed to a maximum number of five per cage in individually ventilated cages (IVC Sealsafe GM500, Tecniplast). Cages contained hardwood bedding (TAPVEI), nesting material, shredded paper, gnawing sticks and a cardboard box shelter (Scanbur). The mice received regular chew diet (either R70 diet or R34, Lantmännen Lantbruk, or CRM-P, 801722, Special Diet Services). General housing parameters such as relative humidity, temperature and ventilation followed the European Convention for the Protection of Vertebrate Animals used for Experimental and other Scientific Purposes treaty (ETS No. 123). Briefly, consistent relative air humidity of 50% and 22°C were maintained, and the air quality was controlled with the use of stand-alone air handling units, supplemented with high efficiency particulate air-filtered air. Husbandry parameters were monitored using ScanClime (Scanbur) units. Water was provided in a water bottle, which was changed weekly. Cages were changed every other week. All cage changes were done in a laminar airflow cabinet. Facility personnel wore dedicated scrubs, socks and shoes. Respiratory masks were used when working outside of the laminar airflow cabinet. All experimental procedures on animals were performed following the European Directive 2010/63/EU, local Swedish directive L150/SJVFS/2019:9, Saknr L150, and Karolinska Institutet complementary guidelines for procurement and use of laboratory animals, Dnr. 1937/03-640. The procedures described were approved by the local committee for ethical experiments on laboratory animals in Sweden (Stockholms Norra Djurförsöksetiska nämnd), lic. nr. 131/15, 144/16, 1995/2019 and 7029/2020.

Experimental autoimmune encephalomyelitis (EAE)

For the induction of EAE, the mouse model of MS, animals were injected subcutaneously with an emulsion of MOG35-55 peptide in complete Freud's adjuvant (CFA; EK-2110 kit from Hooke Laboratories), followed by intraperitoneal injection with pertussis toxin in PBS (200 ng per animal) on the day of immunization and with 24 h delay (according to manufacturer's instructions). Control animals underwent the same treatment, but CFA without MOG35-55 peptide (CK-2110 kit from Hooke Laboratories) was used instead. Spinal cord and brains were collected at the peak of the disease when clinical score 3 (representing limp tail and complete paralysis of hind legs) has been reached. Animals that did not reach this clinical score were not analyzed in this study.

METHOD DETAILS

Tissue dissociation for single-cell ATAC-seq experiment

Mice were sacrificed with a ketaminol/xylazine intraperitoneal injection followed by intracardiac perfusion with PBS. Brain and spinal cords were collected and dissociated using the adult brain dissociation kit (130-107-677, Miltenyi), following the manufacturer's instructions, which included myelin debris removal but not the red blood cell removal step.

Single-cell ATAC-seq (10x Genomics)

Immediately after dissociation, cells were stained with DAPI (0.5 μg/ml, D9542, Sigma) and sorted on a FACS Aria III cell sorter (BD Biosciences). Sox10-GFP+/DAPI- cells were collected in PBS + 0.5% BSA and pooled with Sox10-GFP-/DAPI- cells with a 4:1 ratio. The pool of cells was then lysed and washed according to the Demonstrated Protocol: Nuclei Isolation for Single cell ATAC

Sequencing (10x Genomics) as follows: the cells were centrifuged for 10 min at 300 × g and 4°C, resuspended in ATAC lysis buffer (containing 0.1% IGEPAL (CA-630), 0.1% Tween-20, 0.01% Digitonin, 1% BSA, 10 mM Tris-HCl pH 7.4, 10 mM NaCl, 3 mM MgCl2) and incubated on ice for 3 min. After the incubation, wash buffer (containing 0.1% Tween-20, 1% BSA, 10 mM Tris-HCl pH 7.4, 10 mM NaCl, 3 mM MgCl2) was added on top without mixing and the nuclei were centrifuged for 5 min at 500 × g and 4°C. Nuclei were washed once in Diluted Nuclei buffer (10x Genomics) containing 1% BSA and incubated for 60 min at 37°C in tagmentation mix (10x Genomics). The Chromium Single Cell ATAC v1 Chemistry was used to create single-cell ATAC libraries. Two EAE and four CFA-Ctr animals were used for independent replicates. Libraries were sequenced on an Illumina Novaseq 6000 with a 50-8-16-49 read setup and a minimum of 25,000 read pairs per cell.

Plate-based single-cell ATAC-seq (Pi-ATAC)

Immediately after dissociation, cells were fixed in 1% formaldehyde (28906, Thermo Fisher Scientific) for 10 min and quenched with glycine (125 mM) for 5 min at room temperature and then washed and stored in 0.5% BSA in PBS with 0.1% sodium azide at 4°C until further processing. The cells were counted and aliquots of 500.000 cells were centrifuged for 10 min at 1,000 × g and room temperature. Cells were resuspended in lysis buffer (containing 0.05% IGEPAL (CA-630), 10 mM Tris-HCl pH 7.4, 10 mM NaCl, 3 mM MgCl2) and incubated for 5 min at room temperature. After a 20-min centrifugation at 1,000 × g at room temperature, the cells were incubated with anti-GFP antibody (FITC conjugated, 1:100, Ab6662, Abcam) and DAPI (0.5 μg/ml) in PBS containing 5% BSA for 20 min at room temperature. The cells were centrifuged for 10 min at 1,000 × g and resuspended in tagmentation mix (dH2O, 2x TD buffer [Wang et al., 2013] and Tn5 enzyme [Picelli et al., 2014]) and incubated for 30 min at 37°C. For both lysis and tagmentation buffers the volume was scaled up to match the number of cells (50 μl per 50.000 cells). Tagmentation reaction was stopped by addition of 40 mM EDTA. Cells were then centrifuged for 10 min at 1,000 × g and room temperature and resuspended in PBS + 0.5% BSA. Sox10 GFP+/DAPI+ cells were sorted on an Influx (BD Biosciences) in reverse crosslinking buffer (Chen et al., 2018) with single cells in each well of 96-well plates. Also, Sox10 GFP-/DAPI+ cells were sorted as a negative control for the OL lineage. For reverse crosslinking, the plates were incubated overnight at 65°C ending with 10 min at 80°C the next day. PCR master mixes (NEBNext High fidelity, M0541S, NEB) containing unique barcoding primers per well were dispensed on top of the reverse crosslinking buffer and DNA was amplified with the following cycling conditions: 72°C for 5 min, 98°C for 30s; 20 cycles at 98°C for 10s, 63°C for 30s, and 72°C for 1 min. PCR products were purified with the MinElute purification kit (Qiagen) and then PAGE purified to remove adapter dimers. Three EAE and two CFA-Ctr animals were used for independent replicates. Libraries were sequenced on an Illumina Hiseq 2500 with a 50-8-8-50 read setup and a minimum of 25,000 read pairs per cell.

Single-cell multi-omics (10x Genomics)–mouse

Instead of myelin debris removal (Miltenyi), Percoll (Cytiva 17-0891-01) mixed with HBSS was used to generate a percoll gradient of 38% to remove myelin debris. Immediately after debris removal, cells were stained with DAPI (0.5 μg/ml, D9542, Sigma) and sorted on a FACS Aria III cell sorter (BD Biosciences). Sox10-GFP+/DAPI- cells were collected in PBS + 0.5% BSA. The pool of cells was then lysed and washed according to the Demonstrated Protocol: Nuclei Isolation for Single cell ATAC Sequencing (10x Genomics) with modifications from the Demonstrated Protocol: Nuclei Isolation for Single Cell Multiome ATAC + Gene Expression Sequencing (10x Genomics) as follows: the cells were centrifuged for 10 min at 300 × g and 4°C, resuspended in ATAC lysis buffer (containing 0.01% IGEPAL (CA-630), 0.01% Tween-20, 0.001% Digitonin, 1% BSA, 1 mM DTT, 1 U/ul RNase inhibitor, 10 mM Tris-HCl pH 7.4, 10 mM NaCl, 3 mM MgCl2) and incubated on ice for 3 min. After the incubation, wash buffer (containing 0.1% Tween-20, 1% BSA, 1 mM DTT, 1 U/ul RNase inhibitor, 10 mM Tris-HCl pH 7.4, 10 mM NaCl, 3 mM MgCl2) was added on top without mixing and the nuclei were centrifuged for 5 min at 500 × g and 4°C. Nuclei were washed once in Diluted Nuclei buffer (10x Genomics) containing 1% BSA, 1 mM DTT and 1 U/ul RNase inhibitor, and incubated for 60 min at 37°C in tagmentation mix (10x Genomics). The Chromium Next GEM Single Cell Multiome ATAC + Gene Expression v1 Chemistry was used to create single nuclei ATAC and RNA libraries from the same cell. Two EAE and two CFA-Ctr animals were used for independent replicates. Libraries were sequenced on an Illumina Novaseq 6000 with a 50-8-24-49 read setup for ATAC (minimum 25,000 read pairs per cell) and a 28-10-10-90 read setup for RNA (minimum 20,000 read pairs per cell).

Single-cell multi-omics (10x Genomics)–human

Tissue samples and associated clinical and neuropathological data were supplied by the Multiple Sclerosis Society Tissue Bank, funded by the Multiple Sclerosis Society of Great Britain and Northern Ireland, registered charity 207495. All procedures used by the Multiple Sclerosis and Parkinson's Tissue Bank at Imperial College London in the procurement, storage and distribution of tissue have been approved by the relevant Multicentre Research Ethics Committee (18/WA/0238). Samples were collected under the IRAS Project ID: 246227. Nuclei were isolated from two 10mg fresh-frozen human gray matter brain samples (PD003 - Male, age 58, Post Mortem Interval (PMI) 9 hours, RNA Integraty Number (RIN) 8.9; PD004 - Male, age 90, PMI 12 hours, RIN 7.8), using Nuclei Pure Prep Nuclei Isolation Kit (Sigma Aldrich) with the following modifications. The tissue was lysed in Nuclei Pure Lysis Solution with 0.1% Triton X, 1mM DTT and 0.4U/ul SUPERase-In™ RNase Inhibitor (ThermoFisher Scientific) freshly added before use, and homogenized with the help first of a 23G and then of a 29G syringe. Cold 1.8M Sucrose Cushion Solution, prepared immediately before use with the addition of1mM DTTand 0.4U/ul RNase Inhibitor, was added to the suspensions before they were filtered through a 30μm strainer. The lysates were then carefully and slowly layered on top of 1.8M Sucrose Cushion Solution previously added in

new Eppendorf tubes. Samples were centrifuged for 45 min at 16,000 × g at 4°C. Pellets were re-suspended in Nuclei Storage Buffer with RNase Inhibitor, transferred in new Eppendorf tubes and centrifuged twice for 5 min at 500 × g at 4°C. Finally purified nuclei were re-suspended in Nuclei Storage Buffer with RNase Inhibitor, stained with trypan blue and counted using Countess II (Life technology). After count, nuclei permeabilization was carried out following the demonstrated protocol for Single Cell Multiome ATAC + Gene Expression Sequencing from 10x Genomics. A total of 12,000 estimated nuclei from each sample were used for the transposition step and then loaded on the Chromium Next GEM Single Cell Chip J. ATAC library and gene expression library construction was performed using the Chromium Next GEM Single Cell Multiome ATAC + Gene Expression kit, according to the manufacturer's instructions. Libraries were sequenced using Illumina NovaSeq 6000 System and NovaSeq 6000 S2 Reagent Kit v1.5 (100 cycles), aiming at a minimum sequencing depth of 30K reads/nucleus.

Tissue dissociation for primary OPC cultures

Brains from P4-P6 mouse pups were collected and dissociated with the neural tissue dissociation kit (P; 130-092-628, Miltenyi), according to the manufacturer's protocol. OPCs were obtained by either FACS with Sox10-GFP+ selection or by MACS with CD140a microbeads (Cd140a microbead kit, 130-101-547, Miltenyi). For each experiment, multiple brains were pooled to obtain a sufficient number of cells.

Primary OPC culture

Cells were seeded on poly-L-lysine (P4707, Sigma) coated plates (150,000 cells in 3.8cm^2 -12 well) and grown with OPC proliferation media consisting of DMEM/F12, GlutaMAX (10565018, Thermo Fisher Scientific), N2 supplement (17502001, Thermo Fisher Scientific) NeuroBrew 21 (130-097-263, Miltenyi), penicillin-streptomycin (50 Units/ml penicillin, 50 μg/ml streptomycin, 15140122, Thermo Fisher Scientific), PDGFaa (20 ng/ml, 315-17, PeproTech) and bFGF (40 ng/ml, 100-18B, PeproTech). Cells were treated with IFNγ (100 ng/ml, 485-MI-100, R&D) for 48 h.

EZH2 inhibition

OPC primary cells were treated with EZH2 inhibitor (EPZ011989, 1.5 μM, S7805, Selleckchem) for 4 days, renewing the inhibitor every 48 h. After 4 days, IFNγ (100 ng/ml) was added for 6 h together with 1.5 μM EZH2 inhibitor.

Transcription factor knockdowns

For the transcription factor knockdowns siGENOME SMARTpool siRNA targeting non-target Ctr (D-001206-13-05, Dharmacon), *Stat1* (M-058881-02-0005, Dharmacon), or *Bach1* (M-042956-01-0005, Dharmacon) was used, which contains a pool of 4 siRNA's. 1 μg of siRNA was diluted in OPTIMEM (31985062, Gibco), mixed with lipofectamine2000 (11668027, Invitrogen) and allowed to form complexes for 20 min at room temperature. OPC primary cells were then incubated with the siRNA complexes in DMEM/F12, GlutaMAX (10565018, Thermo Fisher Scientific) media without any supplements for 4 h after which the medium was replaced with normal OPC proliferation media. After 48 h, IFNγ (100 ng/ml) was added for 6 h.

Bulk ATAC-seq

ATAC-seq was performed as previously described (Buenrostro et al., 2013) with minor adaptations. Primary OPCs were incubated with TrypLE (Gibco #12605010) at 37°C for 5 min and collected in cell culture media. 60,000 cells per condition were washed with PBS and lysed with lysis buffer (containing 0.1% IGEPAL (CA-630), 10 mM Tris-HCl pH 7.4, 10 mM NaCl, 3 mM MgCl2) and centrifuged for 20 min at 500 × g and 4°C. Cells were then resuspended in tagmentation mix (dH2O, 2x TD buffer [Wang et al., 2013] and Tn5 enzyme [Picelli et al., 2014]) for 30 min at 37°C. The DNA was purified pre- and post-PCR with the MinElute purification kit (Qiagen) and then PAGE purified to remove adapter dimers. Three replicates per condition were performed with primary OPCs obtained from different litters. Libraries were sequenced on an Illumina Novaseq 6000 with a 50-8-8-50 read setup.

RNA extraction, cDNA synthesis and qRT-PCR

Cells were collected with qiazol (Qiagen) and stored at -80°C until further processing. RNA was extracted with the miRNeasy mini kit (Qiagen), according to the manufacturer's instructions. Contaminating DNA was degraded by treatment of the samples with RNase-free DNase (Qiagen) in column. 350 ng RNA was used to synthesize cDNA with the High-Capacity cDNA Reverse Transcription kit (Applied Biosystems) including RNase inhibitor (Applied Biosystems), with annealing for 10 min at 25°C and extending for 2 h at 37°C and inactivation for 5 min at 85°C. The cDNA was diluted 1:5 in H_2O and 2,5 μl was used in the qRT-PCR reactions with 5 μl of Fast SYBR® Green Master Mix (Applied Biosystems) and 5 pmol of each primer in a final volume of 10 μl. The reactions were run on a StepOnePlus™ System (Applied Biosystems) in duplicate and with reverse transcriptase negative reactions to control for genomic DNA. The running conditions were 20 s at 95°C , followed by 40 cycles of 3 s of 95°C and 30 s of 60°C , then 15 s at 95°C , 1 minute at 60°C , and 15 s at 95°C . Melt curves were generated to control for primer dimers and gene-specific peaks. Relative standard curves for each gene were generated to obtain relative expression values. *Ubc* and *b-Act* were run as housekeeping genes. Expression levels were then calculated by dividing the relative expression value by the value of the geometric mean of the housekeeping genes. Samples were normalized per experiment. Three or four replicates per experiment were performed with primary OPCs obtained from different litters. Primers were designed with the qPCR primer database (Zeisel et al., 2013), sequences used are as follows: *Ubc*-fw

CellPress
OPEN ACCESS

5′-agg tca aac agg aag aca gac gta-3′, *Ubc*-rv 5′-tca cac cca aga aca agc aca-3′, *b-Act*-fw 5′-ctc tgg ctc cta gca cca tga aga-3′, *b-Act*-rv 5′-gta aaa cgc agc tca gta aca gtc cg-3′, *Nlrc5*-fw 5′-ccg tgg tac tca cat ttg cc-3′, *Nlrc5*-rv 5′-cct tcg aga tct ctg gga ca-3′, *Psmb8*-fw 5′-cct tac ctg ctt ggc acc at-3′, Psmb8-rv 5′-atg ctg cag aca cgg aga tg-3′, *H2-q7*-fw 5′-ata cct gca gct cgg gaa g-3′, *H2-q7*-rv 5′-agc acc ata aga cct ggg gt-3′, *Ciita*-fw 5′-ctg gca cag gtc tct cca gt-3′, *Ciita*-rv 5′-tac tga ggc tgc ttg aag gg-3′, *H2-aa*-fw 5′-gct cag cct ctg tgg agg t-3′, *H2-aa*-rv 5′-tgt act ggc caa tgt ctc ca-3′, *H2-ab1*-fw 5′-gca gac aca act acg agg gg-3′, *H2-ab1*-rv 5′-agt gtt gtg gtg gtt gag gg-3′, *Cd74*-fw 5′-ctg gat gaa gca gtg gct ct-3′, *Cd74*-rv 5′-ccc agg cca gaa gat agg tc-3′, *Stat1*-fw 5′-aac atg ctg gtg aca gag cc-3′, *Stat1*-rv 5′-tgc caa ctc aac acc tct ga-3′, *Bach1*-fw 5′-tcg aga atc gta ggc cag ac-3′, *Bach1*-rv 5′-tgg cag tgt agg caa act ga-3′, *Hmox1*-fw 5′-gcc gag aat gct gag ttc at-3′, *Hmox1*-rv 5′-tcc agg gcc gtg tag ata tg-3′.

RNA-seq

0.1–1 μg RNA was used to make RNA-seq libraries with the TruSeq Stranded Total RNA Library Prep Gold kit (Illumina), according to the manufacturer's instructions. Four replicates per condition for the IFNγ experiment, three replicates for the EZH2i experiment, and four replicates for each of the transcription factor knockdowns were performed with primary OPCs obtained from different litters. Libraries were sequenced on an Illumina Novaseq 6000 with a 150-8-8-150 read setup.

Cut&Run

Cut&Run was performed as previously described (Skene and Henikoff, 2017) with minor adaptations. Primary OPCs were incubated with TrypLE (Gibco #12605010) at 37°C for 5 min and collected in cell culture media. Cells were centrifuged for 5 min at 300 × g and room temperature and then resuspended in wash buffer (20 mM HEPES pH 7.5, 150 mM NaCl, 0.5 mM Spermidine, 0.01 % BSA, 1x Roche Complete Protease Inhibitor tablet). 250,000 cells per condition were centrifuged for 3 mint 600 × g and room temperature and resuspended in wash buffer. Activated Concanavalin-A beads (Bangs Laboratories BP531) in binding buffer (20 mM HEPES pH 7.5, 10 mM KCl, 1 mM CaCl2, 1 mM MnCl2) was added to each condition and incubated for 10 min on a rotator at room temperature. Beads with nuclei were now kept on a magnetic stand and washed with Dig-wash buffer (0.05% digitonin in wash buffer). After discarding the liquid, beads were resuspended with primary antibody in antibody buffer (2 mM EDTA in Dig-wash buffer) and incubated overnight at 4°C on a nutator. Then beads were washed with freshly prepared Dig-wash buffer and incubated with 2 μg/ml Protein A-MNase (Schmid et al., 2004) in Dig-wash buffer for 1 h at 4°C on a nutator. After two washes with Dig-wash buffer and one wash with low-salt rinse buffer (20 mM HEPES pH 7.5, 0.5 mM spermidine, 0.05% digitonin) ice-cold incubation buffer (3.5 mM HEPES pH 7.5, 10 mM CaCl2, 0.05% digitonin) was added to the beads which were subsequently placed in a metal block in an ice-water bath maintained at 0°C for 5 min. The beads were then placed on a magnet stand and liquid discarded. Stop buffer (170 mM NaCl, 20 mM EGTA, 0.05% digitonin, 50 μg/mL RNase A, 25 μg/mL glycogen, 2 pg/ml Yeast spike-in DNA) was added to the beads and incubated for 30 min at 37°C. Beads were placed on a magnet stand and supernatant was collected. 2 μL of 10% SDS and 2.5 μL of 20 mg/ml Proteinase K was added to the supernatant and incubated for 1 h at 50°C. DNA from the samples was purified using the MinElute PCR purification kit (Qiagen), according to the manufacturer's instructions. Antibodies were used against H3K27me3 (Cell Signaling 9733S; rabbit; 1 μg), H3K4me3 (Diagenode C15410003-50; rabbit; 1 μg), H3K27ac (Abcam ab177178; rabbit; 1 μg) and CTCF (Cell Signaling 3418S; rabbit; 1:100).

Sequencing libraries were prepared using KAPA HyperPrep kit (Roche #07962363001) and KAPA Unique Dual-Indexed Adapters (Roche #08861919702), according to the manufacturer's instructions, but with the following adjustments: two post-adapter ligation clean-ups were performed using 0.7 × and 1.1 × AMPure XP beads, respectively. PAGE purification was performed on the post-amplification libraries to remove remaining adapter dimers. Two or three replicates per condition for the IFNγ experiment and three replicates for the EZH2i experiment were performed with primary OPCs obtained from different litters. Libraries were sequenced on the Illumina Novaseq 6000 with a 50-8-8-50 read setup.

CUT&Tag for STAT1

CUT&Tag was performed as previously described (Kaya-Okur et al., 2019) with minor adaptations. Primary OPCs were washed from the plate with PBS at room temperature. Cells were centrifuged for 5 min at 300 × g and room temperature and then resuspended in wash buffer (20 mM HEPES pH 7.5, 150 mM NaCl, 0.5 mM Spermidine, 1 × Roche Complete Protease Inhibitor tablet). 100,000 cells per condition were centrifuged for 5 min at 300 × g and room temperature and resuspended in wash buffer. Activated Concanavalin-A beads (Bangs Laboratories BP531) in binding buffer (20 mM HEPES pH 7.5, 10 mM KCl, 1 mM CaCl2, 1 mM MnCl2) was added to each condition and incubated for 10 min on a rotator at room temperature. Beads with nuclei were now kept on a magnetic stand and washed twice with Antibody buffer (2mM EDTA, 0.1% BSA and 0.05% digitonin in wash buffer). After discarding the liquid, beads were resuspended with primary antibody (STAT1, Cell Signaling 14994S; rabbit, monoclonal, 1:100) in antibody buffer and incubated overnight at 4°C on a rotator. Then beads were washed with freshly prepared Dig-wash buffer (0.05% digitonin in wash buffer) and incubated with secondary antibody (guinea pig anti-rabbit, NBP1-72763, Novus biologicals, 1:100) in Dig-wash buffer on a rotator for 30–60 min. After three washes with Dig-wash buffer, the beads were incubated with pA-Tn5 (Kaya-Okur et al., 2019) adapter complex (1:250) in Dig-300-wash buffer (300mM NaCl and 0.05% digitonin in wash buffer) and incubated for 1 h at room temperature on a rotator. After two washes with Dig-300-wash buffer, the beads were resuspended in tagmentation buffer (10 mM MgCl2, 300mM NaCl and 0.05% digitonin in wash buffer) and incubated at 37°C for 1 h in a PCR cycler with heated lid. To stop the tagmentation reaction and to solubilize DNA fragments, 2.5 μL of 0.5M EDTA, 5 μL 10% SDS, and 2 μL of 20 mg/ml Proteinase K were added

to the beads, followed by an incubation of 1 h at 55°C. DNA from the samples was purified using the Zymo DNA Clean & Concentrator kit (D4014, Zymo), according to the manufacturer's instructions, then amplified according to the number of cycles analyzed with qRT-PCR. SPRI bead (B23317, Beckman Coulter) purification was performed on the post-amplification libraries to remove remaining adapter dimers. Three replicates per condition were performed with primary OPCs obtained from different litters. Libraries were sequenced on the Illumina Nextseq 500/550 with 37-8-8-37 bp setup.

H3K27ac-HiChIP

HiChIP was performed as previously described (Mumbach et al., 2016) with minor adaptations. Primary OPCs were incubated with TrypLE (Gibco #12605010) at 37°C for 5 min and collected in cell culture media. 1-3 million cells were washed once with PBS and crosslinked using freshly prepared 1% formaldehyde (Methanol-free, Pierce, 28906) diluted in PBS for 10 min at room temperature with gentle rotation. Formaldehyde was quenched by addition of glycine (125 mM) and incubated for 5 min at room temperature with gentle rotation. Fixed cells were then washed once with ice-cold PBS, flash frozen, and stored at − 80°C until further processing. Chromatin was sonicated using the covaries ME220 with settings 75 PIP, 5% duty cycle, and 200 cycles/burst for 2 min (for 1–3 milion cells). The immunoprecipitation was performed using 2 μg H3K27ac antibody (Abcam, Ab177178) and 20 μl protein A dynabeads (Thermo Fisher, 007613560), with 0.75 μl in-house produced Tn5 for tagmentation, and 15–16 cycles of final PCR amplification (NEBNext High Fidelity 2x PCR mastermix, M0541L). Barcoded libraries were gel-purified, quantified using bioanalyzer, and mixed in equimolar ratio. Three replicates per condition were performed with primary OPCs obtained from different litters. Libraries were sequenced on an Illumina Novaseq 6000 with a 50-8-8-50 read setup.

Western Blot

Cells were collected in 2x Laemmli buffer (120 mM Tris-HCl pH 6.8, 4% SDS, 20% glycerol) and sonicated for 5 min at high power with 30s on/off cycles at 4°C. Protein concentrations were measured with nanodrop and equalized with 2 × Laemmli buffer. Bromophenol blue (0.1%) and B-Mercaptoethanol (10%) were added to the protein prior to a 5-min incubation at 95°C to denature the protein. Equal volumes were loaded on 4%–20% Mini-Protean TGX precast protein gels (4561094, Bio-Rad) and transferred to a PVDF membrane (GE Healthcare) activated in methanol. Membranes were then blocked in blocking buffer (containing TBS, 0.1% Tween-20 and 5% BSA) for 1 h at room temperature and incubated overnight with primary antibody (diluted in blocking buffer) at 4°C. The membranes were then washed 3 times 10 min in TBS-t (TBS, 0.1% Tween-20) and incubated with a horseradish peroxidase-conjugated secondary antibody for 2 h at room temperature. Proteins were exposed with ECL prime (GE Healthcare) at a ChemiDox XRS imaging system (Bio-Rad). Primary antibodies were used against H3K27me3 (rabbit monoclonal, 9733S, Cell Signaling, 1:1000) or GAPDH (rabbit monoclonal, 5174S, Cell Signaling, 1:1000) and as secondary antibody anti-rabbit (A6667, Sigma, 1:5000)

scATAC-seq 10X Genomics preprocessing

scATAC-seq (10X Genomics) data were processed with default parameters with cellranger-atac (version 1.2.0) *count* function. Reads were aligned to mm10 reference genome. As part of cellranger-atac pipeline, peaks were called individually for each of the samples and then merged. Normalized single peak-barcode matrix combining all the samples was calculated with the parameter cellranger-atac *aggr –normalize=depth*, by subsampling all the fragments to the same effective depth to avoid batch effects introduced by sequencing depth, which resulted in a median fragments per cell of 21836. The number of fragments in peaks, the fraction of fragments in peaks, and the ratio of reads in ENCODE blacklist sites computed by cellranger-atac were used as QC metrics in downstream processing with the package Signac v0.25 https://satijalab.org/signac/. scATAC 10X Genomics cells with the following metrics were selected: peak_region_fragments > 1000 & peak_region_fragments < 20000 & pct_reads_in_peaks > 15 & blacklist_ratio < 0.05 & nucleosome_signal < 10 & TSS_enrichment_score > 2, which resulted in 4895 cells.

scATAC-seq Pi-ATAC preprocessing

scATAC-seq (Pi-ATAC) data were processed following the https://carldeboer.github.io/brockman.html pipeline (de Boer and Regev, 2018). Reads were trimmed and aligned to mm10 reference genome using Bowtie2 (Langmead and Salzberg, 2012). Reads with alignment quality less than Q30, incorrectly paired, and mapped to mitochondria were discarded. Duplicates were removed using Picard tools. Peaks were called individually for each sample using MACS2 (Zhang et al., 2008) (https://github.com/macs3-project/MACS) with the following parameters *-q 0.05 –nomodel -molambda –shift -100 –extsize 200 -call-summits*. Called peaks were merged using bedtools *mergebed* and the peaks overlapping ENCODE blacklisted regions were removed. The summit peaks were resized and extended to the same size and used as input in chromVAR (Schep et al., 2017) to get the fragment counts in peaks, using as input all the individual cell bam files. Fraction of fragments per peak was calculated and filtered using chromVAR resulting in 1029 cells. The output fragments matrix and peak annotations were used as input for Seurat (Butler et al., 2018) to perform non-linear dimension reduction, normalization and clustering.

Normalization and clustering of scATAC-seq

Normalization and linear dimensional reduction were performed with Signac (Butler et al., 2018). Signac v1.1.0 (Stuart et al., 2020) first performs a frequency-inverse document frequency normalization (TF-IDF), which normalizes across cells and peaks. Then, a feature selection was performed using all the peaks as input. The dimensional reduction was performed on the TF-IDF normalized

CellPress
OPEN ACCESS

matrix with the selected peaks using a singular value decomposition (SVD). RunUMAP, FindNeighbors and Findclusters functions from Seurat v3.2.1 (Butler et al., 2018) were used for clustering and visualization with 30 dimensions.

Gene activity scores and integration with scRNA-seq data

We assumed correlation between promoter accessibility and gene expression. First, different promoter lengths were tested (2 Kb, 1 Kb, 500 bp and including the region around the TSS). We extracted the gene coordinates and extended them to include the different promoter lengths. Then, the number of reads from the pooled scATAC-seq samples intersecting the coordinates were counted to calculate a pseudobulk accessibility score. Using scRNA-seq from Falcão et al. (2019), we generated pseudo-bulk scRNA-seq signal for each of the annotated OLG and MiGl cell types and calculated normalized gene expression for the pooled cells. We directly correlated the pooled scATAC accessibility score over the tested promoter regions and pooled gene expressions. The promoter length with highest Spearman correlation coefficient between pseudobulk scATAC and scRNA samples was selected, 500 bp. However, 500-bp promoters only showed a slight improvement in the correlation value, 500 bp Spearman correlation rho for 500bp ~0.62, 1Kb ~0.59 and 2Kb ~0.56.

Final gene activities were computed over the 500-bp region upstream of the TSS of annotations with the ENSEMBL79 biotype protein_coding using Signac. scATAC-seq cells were annotated based on scRNA-seq data from Falcão et al. (2019). The shared correlation patterns between gene activity and the scRNA-seq annotated expression matrix from Falcão et al. (2019) were calculated in Signac/Seurat (Butler et al., 2018) with FindTransferAnchors function, using as reference precomputed scRNA-seq and query scATAC activity scores. Using the classification score of minimum 0.4 the scATAC-seq cells were annotated. Manual checking of the classification scores identified mismatches from incorrect classified cells, where some cells from EAE or Ctr were incorrectly classified, we manually curated the final annotations and discarded cells that showed ambiguity. A small subset of cells from EAE mice were classified as Ctr-MOL populations and vice versa, revealing a certain degree of ambiguity in the promoter-based scATAC-seq classification score (Figure S1D). These cells were manually corrected as MOL-EAE and MOL-Ctr (Figure 1D) and not included in further analysis directly comparing the two conditions. Single-cell tracks were obtained with samtools 1.10 using the CB tag from cellranger-atac aligned bam files and the cluster cell type annotations.

Differentially accessible peaks

Differential accessibility was calculated between cell type clusters and within cell types between EAE and Ctr conditions with Signac with parameters *min.pct = 0.2, test.use = LR , latent.vars = peak_region_fragments*. For each of the peaks the closest gene was found using closestFeature function combined with EnsDb.Musmusculus.v79 annotations. Identified genes were used in downstream analyses such as, for instance, GO. Unique peaks and gene lists per cluster cell type were obtained by comparing unique lists of candidates with adjusted p value less than 0.05.

Gene ontology analysis

GO analysis was performed with ClueGO (version 2.5.5) (Bindea et al., 2009) plug-in Cytoscape (version 3.7.2) (Smoot et al., 2011) with the following settings: GO Biological process, minimum GO level: 3, Max GO level: 8, minimum number of genes: 3, minimum percentage 4.0, correction methods: bonferroni step down, p value cutoff: 0.05. GREAT v4.0.4 (McLean et al., 2010) was performed on EAE-enriched peaks regions for MOL1/2, MOL5/6 and OPCs Mouse: GRCm38 (UCSC mm10, Dec 2011) with whole-genome background regions and basal plus extension gene regulatory domain definition (with proximal 5 kb upstream, 1 kb downstream, plus distal up to 1,000 kb on GO biological process categories and mouse genotype-phenotype associations mapped to human genes. Combined statistical tests include the binomial test over genomic regions and the hypergeometric test over genes. The significance threshold used for FDR corrected q-values was 0.05.

scATAC peaks annotations

Peaks were annotated using HOMER v4.11 (Heinz et al., 2010) with annotatePeaks.pl and gencode.vM20 annotations for all the peaks used in the analysis and for the set of peaks that showed differential accessibility between EAE- and Ctr-OLG. For the donut plots the basic annotations are shown.

MS-associated SNPs

To enable comparison between mouse open chromatin regions and human MS-associated SNPs, liftOver was used with parameters *minMatch=0.5* to convert mm10 coordinates to hg19 genomic coordinates. Then, as a double check, we reciprocally lifted back the coordinates to mm10 and retrieved only the peaks that mapped to the original position. To define the set of peaks used in the analysis, the properly aligned reads from annotated OLG cell types and MiGl were combined to generate pseudobulk ATAC-seq bam files specific for each cell type. Then, peak calling was performed for each of the annotated cell types, MACS2 with parameters *-q 0.05 –nomodel -molambda –shift -100 –extsize 200*. Peaks were sorted and merged to non-overlapping meta peaks. Using bedtools *intersect -wo* the set of SNPs overlapping open chromatin regions were retrieved. In Figure 8, the signal of the mouse scATAC-seq is shown in mm10 reference, signal of the human scATAC-seq is shown in hg38 and the SNPs coordenates have

been liftOver to the correspondent reference. MOL, MiGl, and OPC scATAC-seq peaks from healthy donors in hg38 (Corces et al., 2020) were liftOver to hg19. Using bedtools *intersect –wo* with the selected SNPs with evidence from mouse scATAC-seq liftOver peaks the intersecting hg19 peaks were retrieved.

Cell type specific (CTS) LD score regression (https://github.com/bulik/ldsc/wiki/Cell-type-specific-analyses) (Finucane et al., 2018) was used to estimate the enrichment coefficient of each OLG and MiGl cluster with different sets of Ctr peaks and a merged set of all significant peaks in the study as background. Gene set enrichments were calculated with MAGMA (de Leeuw et al., 2015) v1.06 with the following parameters: –annotate_window = 10,1.5 –gene_loc = NCBI37.gene.loc with specifically formatted summary statistics. Summary statistics and SNPs locations were obtained from International Multiple Sclerosis Genetics Consortium (2019) and Factor et al. (2020).

MS GWAS loci overlapping

The GREGOR suite (Schmidt et al., 2015) was used for calculating the intersection of MS variants GWAS loci in peaks of interest: (1) specific for MiGl, OPC, MOL2, and MOL56 in EAE and controls from the scATAC-seq, (2) significant peaks from bulk ATAC-seq IFNγ induced and Ctr, (3) scATAC-seq peaks specific for OLIGO, MiGl , InhNeu (Inhibitory neurons), ExcNeu (Excitatory neurons), NigNeu (Nigral neurons), ASTRO (Astrocytes), and OPCs from human control samples, and (4) scATAC-seq peaks specific for OLIGO, MiGL, OPCs, ASTRO (Astrocytes), EXCTNEU (Excitatory neurons), INHNEU (Inhibitory neurons), ENDO (endothelial) from human brain gray matter single-cell multi-omics samples, as shown in Table S8. As a random control each peak set was shuffled using bedtools. LiftOver to hg19 was used for mouse datasets as explained above.

TF motifs differential accessibility

Motif activity between single cells was calculated using chromVAR (Schep et al., 2017). Motif variability was calculated for all cells on the selected peaks from Seurat/Signac (Butler et al., 2018) analysis with chromVARmotifs library, mouse_pwms_v2, motifs. For visualization purposes the top 100 most variable motifs were selected to build a matrix of the normalized deviation scores, *Z* scores, as is shown in the heatmap in Figure 4A. Deviation *Z* scores from chromVAR are shown on the UMAP coordinates in Figure 4B.

Signac was used to identify the overrepresented TF motifs in the sets of differentially accessible peaks between cell types and between EAE versus Ctr in OLG. Signac performs a hypergeometric test to get the probability of observing a specific motif at a given frequency by chance. The motif enrichment was performed with the chromVARmotifs PWM mouse_pwms_v2 library. Then, we cross-checked expression levels of the significantly enriched motifs in the scRNA-seq data from (Falcão et al., 2019) to select a set of TFs for further analysis, Table S4.

The distribution of the binding sites was annotated using HOMER (Heinz et al., 2010) annotatepeaks.pl with the basic annotations. The closest genes were assigned to the predicted binding sites to count the number of genes per cell type cluster for each specific TF.

Bulk ATAC-seq alignment and peak calling

ATAC-seq samples were processed separately following the standard ENCODE pipeline for ATAC-seq samples, https://www.encodeproject.org/pipelines/ENCPL792NWO/. Adapters were detected and trimmed, and reads were aligned to mouse genome (GRCm38/mm10) using Bowtie2, with default parameters. After filtering mitochondrial DNA, reads properly paired were retained and multimapped reads, with MAPQ< 30, were removed using SAMtools (Li et al., 2009). PCR duplicates were removed using Mark-Duplicates (Picard - latest version 1.126), http://broadinstitute.github.io/picard/. ATAC-seq peaks were called using MACS2 https://github.com/taoliu/MACS, with parameters -g mm -q 0.05–nomodel–shift −100–extsize 200 -B –broad. For visualization and further analyses, the replicates were merged using SAMtools (Love et al., 2014) and tracks were normalized using Deeptools bamcoverage.

Bulk RNA-seq alignment and differential gene expression

The bulk RNA-seq samples were preprocessed for adapter/quality trimming and then aligned to the transcriptome using STAR (Dobin et al., 2013) version 2.7 –quantMode –sjdbOverhang 99 with EnsEMBLv75 gtf annotations. Only uniquely mapped reads were retained for downstream analysis using SAMtools. Aligned samples were converted to bedgraph files using Deeptools bamcoverage for each strand and normalized to total of reads. Filtered fastq files were used in Salmon 0.8.2 to recover the raw reads counts and transcript per million (TPM) values per transcript and gene. The differential gene expression analysis was performed with Deseq2 (Love et al., 2014). Results from differential expression were plot using Enhancedvolcano package with $\log_2$ fold change and adjusted p value from Deseq2.

CUT&RUN alignment and processing

Cut&Run samples were processed with the pipeline CUT&RUNTools that includes reads trimming, alignment (Bowtie2 mm10 reference genome) and peak calling with MACS2 https://bitbucket.org/qzhudfci/cutruntools/src/master/. For TF Cut&Run samples the narrow peaks with <120bp fragments were used and for histone modifications broad peaks from all the fragments were used in downstream analysis (Zhu et al., 2019).

Differential CUT&RUN enrichments were calculated using pyicos over the previously defined 500-bp promoter regions. Using bedtools intersect bam, all the reads intersecting called peaks were counted and used to calculate enrichment fold change with pyicos

(Althammer et al., 2011) pyicoenrich -counts –pseudocount parameters. *Z* score-associated p value and Benjamini Hochberg corrected p value were computed in R 2*(pnorm(-abs(zscore)) and p.adjust method=BH.

HiChIP

Paired-end sequencing reads from HiChIP experiments were aligned to mm10 genome and filtered for duplicates using the HiC-Pro pipeline. The pipeline's hicpro2juicebox.sh script was used to generate .hic files, which were loaded into Juicebox for viewing contact maps.

ABC model

The ABC model (Fulco et al., 2019) was computed following https://github.com/broadinstitute/ABC-Enhancer-Gene-Prediction. Processed bam files, as explained above, from bulk ATAC-seq and H3K27ac CUT&RUN from Ctr and IFNγ-treated OPCs were provided as input for the model. H3K27ac-HiChIP data from cultured primary OPCs were used to estimate contact frequency. Default parameters were used for generating the candidate enhancer list and for quantifying enhancer activity. Regulatory loops with an ABC score $>$ 0.05 were used for downstream analyses.

Correlation between expression and chromatin accessibility

The pseudobulk scATAC reads per annotated cell type were intersected with Bedtools bedcoverage with 500-bp promoter regions mm10 reference genome EnsEmbl v75 and normalized by total number of reads and region length to get a normalized activity score comparable to normalized expression values. Pseudobulk gene expression and pseudobulk activities at promoters were combined and clustered based on enrichment in EAE compared to Ctr.

For bulk RNA-seq and ATAC-seq Ctr-OPCs and IFNγ-treated OPCs, accessibility was calculated as the bulk ATAC-seq coverage signal on 500-bp promoter regions. Normalized read coverage at 500 bp-promoter regions was calculated as RPKM.

Gene set types definition for single-cell and bulk samples

4 types of genes (with additional subtypes) were defined based on the expression and activity values; the non-redundant list of genes for each type was used to do GO analysis. Type1 (genes with increased expression in EAE and chromatin accessibility), Type2 (genes with increased expression in EAE, but no change in chromatin accessibility), Type3 (genes with reduced expression in EAE, but no change in chromatin accessibility), and Type4 (genes with reduced expression and chromatin accessibility in EAE) gene sets, were defined based on the results from differential gene expression and differential accessibility. Each Type was further divided in subtypes based on ranked expression and chromatin accessibility within the Type. For Ctr-OPCs and IFNγ-treated OPCs, we defined the types (and additional subtypes) as explained above for the single-cell samples. For plotting, the resulting Types ComplexHeatmap (Gu et al., 2016) was used.

Enhancer to promoter interactions from scATAC-seq

Predictions of enhancer to promoter interactions were performed using the R package ArchR (Granja et al., 2020) Peak2gene function with cutoff 0.5 and resolution 1, which integrates scRNA-seq and scATAC-seq information to correlate expression and accessibility.

Predicted putative enhancers coordenates from peak2gene output were used to calculate the aggregated normalized scATAC-seq signal for different gene Types in different celltypes. scATAC-seq signal was counted using; *bedtools multicov -bams* and normalizing thw signal by putative enhancer length and total number of reads.

CUT&RUN peaks intersection

Peaks were annotated with HOMER AnnotatePeaks.pl to basic annotations. All the peaks annotated to genes from Type1 (genes with increased expression in EAE and chromatin accessibility) and Type2 (genes with increased expression in EAE, but no change in chromatin accessibility) were intersect with bedtools intersect –c to Ctr-OPCs and IFNγ-treated OPCs peaks to build the intersection matrix. Upset plots were built with R package Upset.

Number of predicted interactions in Ctr-OPCs and IFNγ treated OPCs

For each of the differentially upregulated genes in IFNγ-treated OPC genes the number of interactions predicted with H3K27ac ABC model was counted in ctr and IFNγ-treated conditions.

Single-cell multi-omics (mouse) data processing

Single-cell multi-ome mouse (10X Genomics) data were processed with default parameters with cellranger-arc (v2.0.0) *count* function. Reads were aligned to mm10 (ata-cellranger-arc-mm10-2020-A-2.0.0) reference genome. Normalized single feature-barcode matrix combining all the samples from both modalities was calculated with the parameter cellranger-arc aggr with default parameters, samples were normalized to depth for both ATAC and gene expression modalities, which resulted in a median fragments per cell of 12,928, median UMI counts per cell of 4,240, median genes per cell of 1,849 and 5,769 cells.

We used Cellranger-arc called peaks for preQC measurements. After QC we run macs2 whithin Seurat/Signac to call the peaks, to recover consistent peak set within the whole dataset. For downstrean analyses, we combined Signac v1.4.0 and Seurat v4.0.0 joint RNA and ATAC analysis. We used as QC metrics nCount_ATAC < 100000 & nCount_RNA < 25000 & nCount_ATAC > 500 & nCount_RNA > 500 & nucleosome_signal < 2 & TSS.enrichment > 1, which resulted in 5,264 cells.

Gene activity was calculated over the 500-bp promoter regions of annotated protein_coding genes from mm10 EnsEmbl79. Pooled replicates cells were identified based on CHRX and CHRY module score calculated from BiomaRt. Data processing was performed in RNA and ATAC modalities separately with Seurat/Signac. RNA modality was integrated with Harmony (Korsunsky et al., 2019) based on sample variable, normalized and dimension reduction PCA, dims=1:50. For the ATAC modality, we performed latent semantic indexing (LSI) on the Harmony integrated space for dims=2:50. Using the weighted nearest neighbor (WNN) from Seurat v4, we computed the joint neighbor graph of RNA and ATAC modalities. We run FindClusters in the ATAC graph, RNA graph, and joint WNN graph. Cells were annotated using *transfer_anchors* on the RNA modality to Falcão et al. (2019) scRNA-seq data and manually curated based on known gene markers, which allowed us to identify Astrocyte cells. By annotating the data to the Falcão et al. (2019) reference, we can compare the cell clusters indentified in the scATAC-seq data to the single-cell multi ome cells clusters. We then called again the peaks using MACS2 on the identified cell clusters.

Single-cell multiomics (human) data processing

Single-cell multiome human (10X Genomics) data samples were processed with default parameters with cellranger-arc (v2.0.0) *count* function. Reads were aligned to GRCh38 (refdata-cellranger-arc-GRCh38-2020-A-2.0.0) reference genome , which resulted for each sample PD003 and PD004 of a median fragments per cell of 21422/22379, median UMI counts per cell of 7407/8189, median genes per cell of 2972/3268 and 5272/4513 cells.

Normalized single feature-barcode matrix combining the two individuals from both modalities was calculated with the parameter cellranger-arc *aggr* with default parameters; samples were normalized to depth for both ATAC and gene expression modalities. We used Cellranger-arc called peaks for preQC measurements. After QC we ran macs2 within Seurat/Signac to call the peaks, to recover consistent peak set within the whole dataset. For downstrean analyses, we combined Signac v1.4.0 and Seurat v4.0.0 joint RNA and ATAC analysis. We used as QC metrics nCount_ATAC < 100000 & nCount_RNA < 100000 & nCount_ATAC > 1000 & nCount_RNA > 1000 & nucleosome_signal < 2 & TSS.enrichment > 1, which resulted in a total of 4,194 and 3,670 cells from each individual sample.

Dimension reduction and Findclusters were performed the same as for single-cell multi-ome mouse data. Samples were integrated using Harmony (Korsunsky et al., 2019) on the patient variable. Cells were annotated using a panel of canonical markers for main brain cell types; 'PLP1','MAG','MOG','OPALIN' for Oligocendrocytes. 'PDGFRA','SOX6','BCAN' for OPCs. 'FGFR3','GFAP','SLC14A1','AQP4' for Astrocytes. 'P2RY12','SPP1','CSF1R','IRF8' for Microglia. 'SLC17A7','FEZF2','RORB' for Excitatory Neurons. 'GAD1','ADARB2','LHX6' for Inhibitory Neurons. 'CLDN5','FLT1','EPAS1', 'EPS8','LAMA2' for Endothelial and Stromal cells. In order to get clear celltypes on the ATAC called peaks, we discarded combinations of canonical markers from different main cell types from the RNA expression. We then called peaks using MACS2 grouped by defined cell type.

Bulk CUT&TAG processing and peak calling

CUT&TAG data processing was following https://yezhengstat.github.io/CUTTag_tutorial/ combined with parameters from bulk ATAC-seq processing. Alignment was performed with Bowtie2, reads were aligned to mouse genome (GRCm38/mm10) with parameters –end-to-end –very-sensitive –no-mixed –no-discordant –phred33 -I 10 -X 700 -p 3 –x . Peaks were called with MACS2 with parameters,_-q 0.1 –keep-dup all. For visualization, we selected upregulated and downregulated genes from STAT1 knockdown, $\log_2$ foldchange 1 and adjusted p value < 0.05, and plot CUT&TAG STAT1 signal around TSS of the selected genes usind Deeptools (Ramirez et al., 2014).

Bulk RNA-seq KD alignment and processing

The bulk RNA-seq samples were preprocessed for adapter/quality trimming and then aligned to the transcriptome using STAR (Dobin et al., 2013) version 2.7 –quantMode –sjdbOverhang 99 with EnsEMBLv75 gtf annotations. Only uniquely mapped reads were retained for downstream analysis using SAMtools. Aligned samples were converted to bedgraph files using Deeptools bamcoverage for each strand and normalized to total of reads. Filtered fastq files were used in Salmon 0.8.2 to recover the raw reads counts and transcript per million (TPM) values per transcript and gene. The differential gene expression analysis was performed with EdgeR (McCarthy et al., 2012; Robinson et al., 2010) for paired samples, CalcNornFactors = TMM and design ~day+condition. Results from differential expression were plot using Enhancedvolcano package with $\log_2$ fold change and FDR from EdgeR.

GSEA in single-cell ATAC-seq

Gene set enrichment analysis was performed using escape (Borcherding et al., 2021) R package with GSEABase (Morgan et al., 2021). *GSEABase: Gene set enrichment data structures and methods*. R package version 1.54.0.) Mus musculus Hallmarks. Escape was run on the ACTIVITY assay; gene activities were calculated as the average ATAC signal per cell on the 500 bp-promoters of protein_coding annotated genes from EnsEmbl79.

Motifs analysis around MS-associated SNP locations

SNPs were extended 500 bp upstream and downstream of the SNP location in hg19.

We used MEME v4.12.0 and run Fimo (Grant et al., 2011) with hg19.5th_order_background_model for CIS-BP (Weirauch et al., 2014) Homo_sapiens.meme PWM on SNP extended regions. For each TF motif we retrieved pseudobulk RNA expression and gene activity from single-cell ATAC-seq and single-cell RNA-seq. MS-associated SNP with predicted changes on TF binding site were extracted from SNP2Tfbs ftp repository *snp2tfbs_JASPAR_CORE_2014_vert.bed* (Kumar et al., 2017).

Neuron, Volume *110*

Supplemental information

Epigenomic priming of immune genes implicates oligodendroglia in multiple sclerosis susceptibility

Mandy Meijer, Eneritz Agirre, Mukund Kabbe, Cassandra A. van Tuijn, Abeer Heskol, Chao Zheng, Ana Mendanha Falcão, Marek Bartosovic, Leslie Kirby, Daniela Calini, Michael R. Johnson, M. Ryan Corces, Thomas J. Montine, Xingqi Chen, Howard Y. Chang, Dheeraj Malhotra, and Gonçalo Castelo-Branco

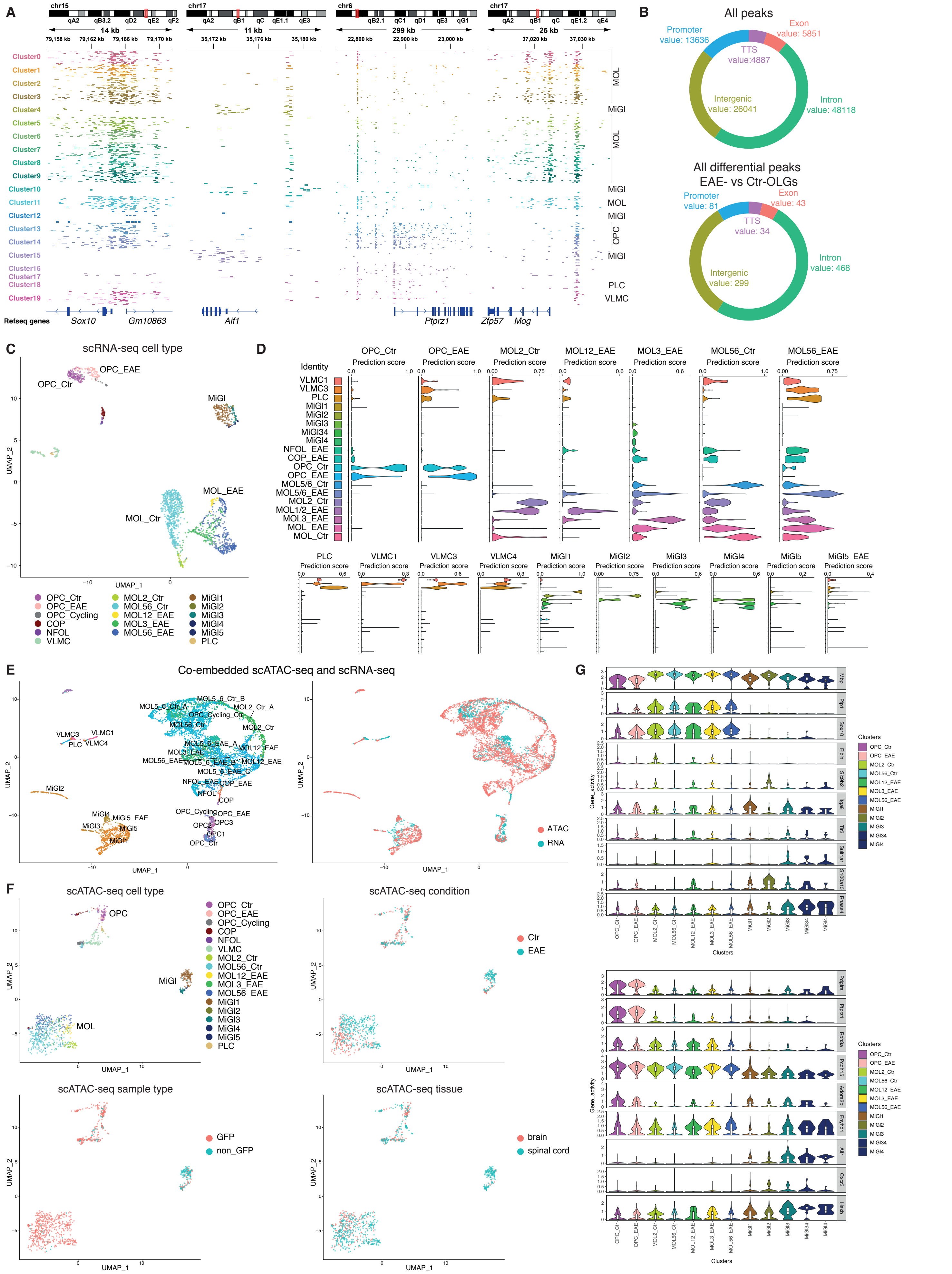

Figure S1. Single-cell ATAC-Seq in the EAE mouse model of MS; integration with single-cell RNA-Seq, related to Figure 1

A, IGV tracks of chromatin accessibility in 50 randomly selected individual cells for each of 20 cell clusters identified with unsupervised clustering. Colors represent the cluster as in Fig. 1b. Marker genes are shown for OLG (*Sox10*), MiGl (*Aif1*), OPCs (*Ptprz1*) and MOLs (*Mog*). Genomic coordinates are shown. **B,** Distribution of accessibility of all peaks in all cells (All peaks) or differential accessibility peaks between EAE- and Ctr-OLG. TTS = Transcription termination site. **C,** UMAP clustering of scRNA-seq data (Falcao *et al.*, 2019), showing the major clusters, colors represent the clusters as in Fig. 1d. **D,** Prediction scores for each population to show the performance of the scRNAseq integration with scATAC-seq data to predict cell-types. **E,** Co-embedding of scATAC-seq data with scRNA-seq data (Falcao *et al.*, 2019). Colors represent the clusters (left) or method (right). **F,** Plate-based scATAC-seq (Pl-ATAC) on GFP+ and GFP- cells (Sox10:Cre-RCE:LoxP(EGFP)) collected from brains and spinal cords from EAE and Ctr mice. Clustering performed with UMAP based on differential accessibility. Shown are cell-types (label transfer from scRNA-seq (Falcao *et al.*, 2019)), condition (EAE vs. Ctr), sample type (GFP vs. non-GFP) and tissue (brain vs. spinal cord).**G,** Violin plots depicting enriched chromatin accessibility at the promoter regions of marker genes identified in scRNA-seq data (Falcao *et al.*, 2019) in the identified scATAC-seq populations.

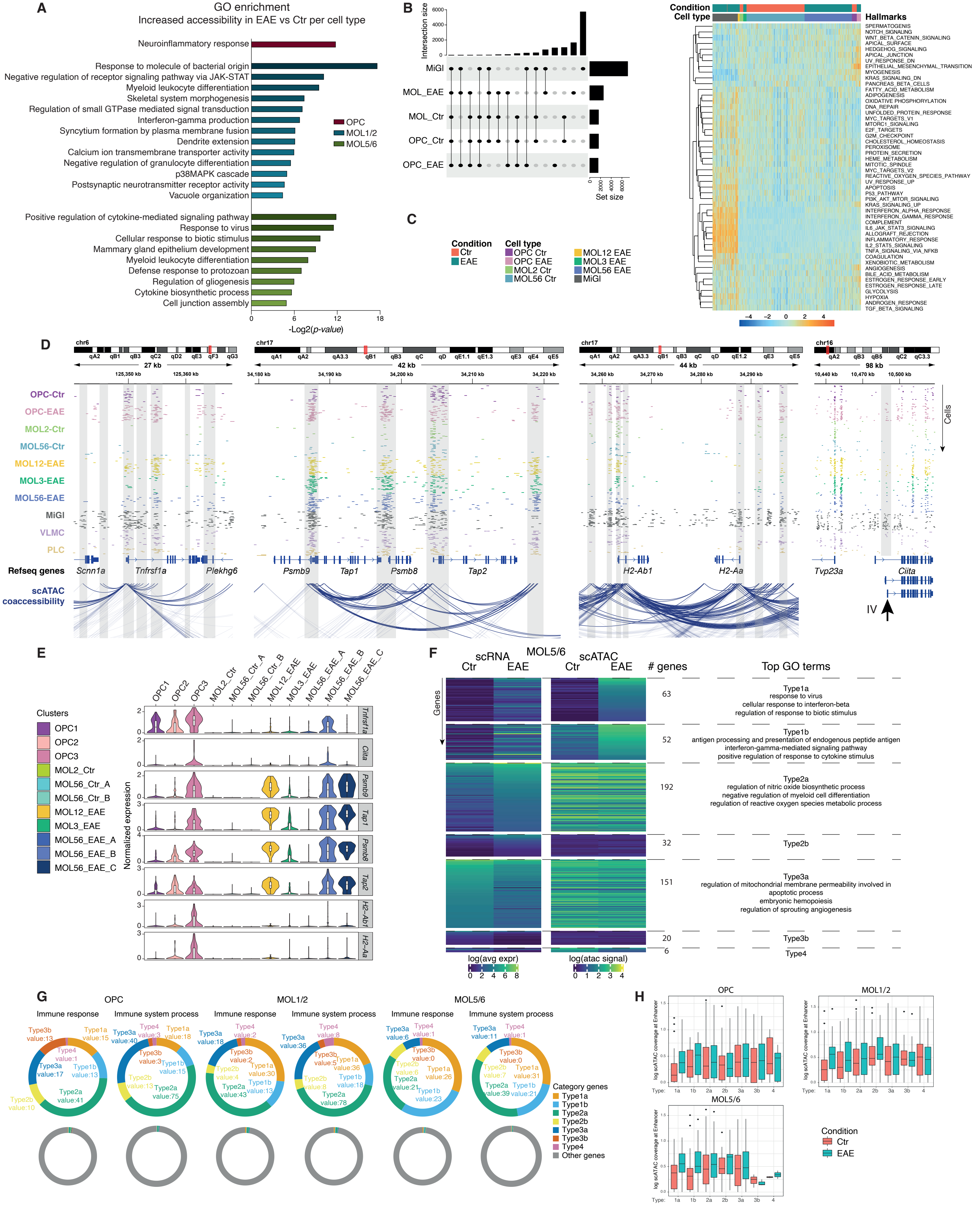

Figure S2. Single-cell ATAC-Seq in EAE mouse model of MS; Gene Ontology, hallmarks and patterns of coverage at promoters and enhancers, related to Figures 1 and 2. A, Gene Ontology enrichment analysis (biological processes) of the nearest genes to enriched accessibility peaks in EAE are shown for OPC, MOL1/2 and MOL5/6 populations. **B**, Upset plot with proportions of the promoter peaks with chromatin accessibility in microglia that also presented chromatin accessibility OLG clusters. **C**, Gene set enrichment analysis (GSEA) of gene loci have open chromatin in OPCs/OLs and MiGl. **D**, Integrative Genomics Viewer (IGV) tracks of chromatin accessibility in 50 randomly selected individual cells for each selected cluster, with MiGl clusters grouped together. scATAC accessibility connections are shown. Highlighted with grey boxes are regions with differential accessibility in specific clusters or promoter priming and connections between regulatory regions. Genomic coordinates are shown. Highlighted with a grey box and arrow is *Ciita* promoter region IV. **E**, Violin plots showing normalized RNA expression values per OLG cluster for selected genes (data from Falcao *et al.*, 2019). **F**, Genes in MOL5/6 are clustered based on gene expression differences between EAE vs. Ctr and correlation with chromatin accessibility activity score (accessibility over 500 bp promoter region). Top GO terms are shown for Type1-4. **G**, Distribution of immune genes found in GO terms as 'immune response' (GO:0002250) and 'immune system process' (GO:0002376) within the different RNA/chromatin accessibility subtypes define in Figure 2. Only a minority of genes in these immune GO categories are expressed or present open chromatin in OLG (bottom row). **H**, Normalized aggregated scATAC coverage over the putative enhancers (as predicted by ArchR) of Type 1-4 genes (from Figure 2a and S2F), in OPCs, MOL1/2 and MOL5/6 from Ctr and EAE conditions.

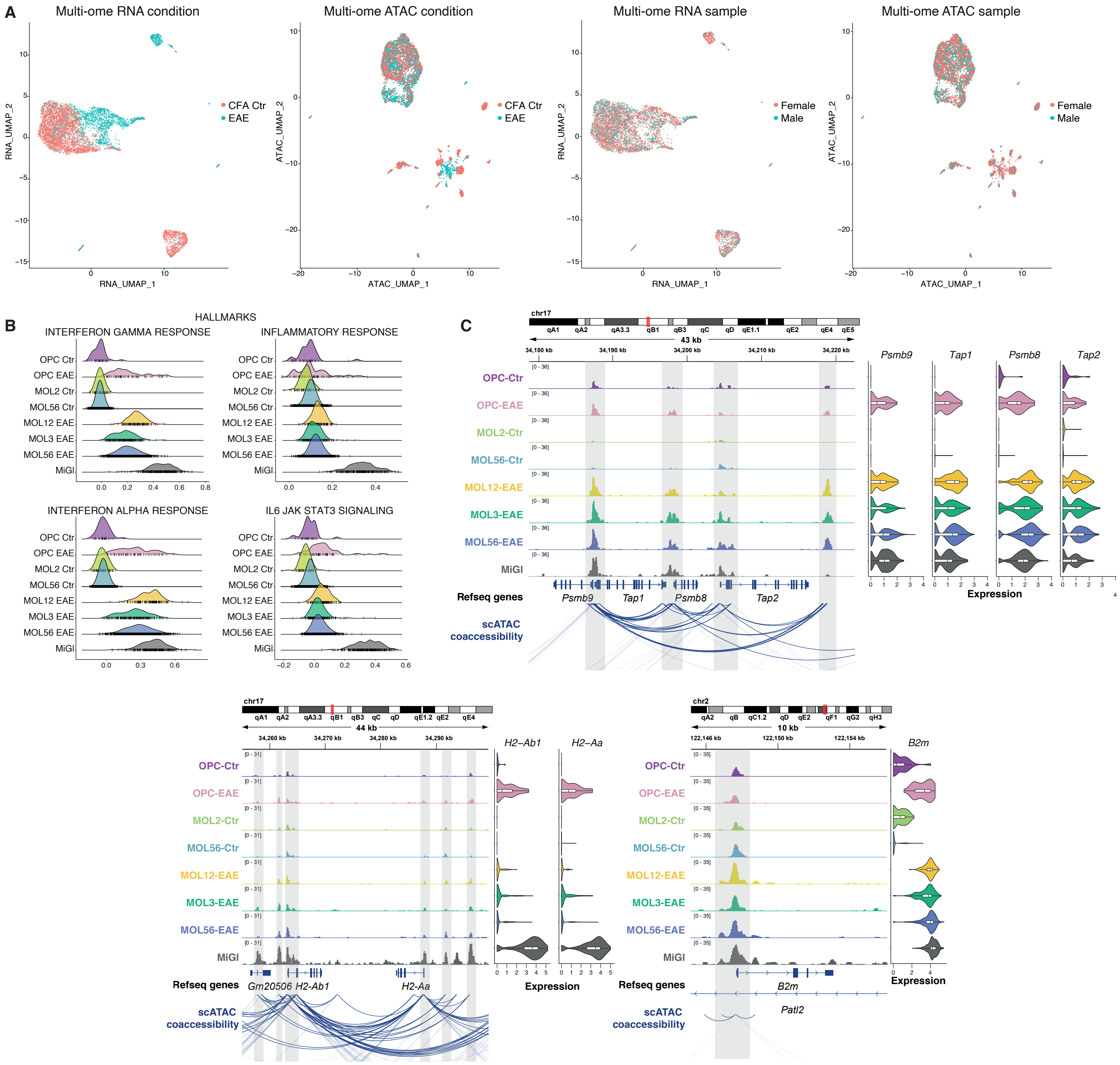

Fig S3. 10x Genomics multi-ome (simultaneous single cell chromatin accessibility and transcriptomics) of Sox10-GFP cells sorted from the spinal cord of Ctr (2) and EAE mice, related to Figure 2.

A, UMAP based on chromatin accessibility and transcriptome. Shown are condition (EAE vs. Ctr), sample type (GFP vs. non-GFP) and sex.

B, Gene set enrichment analysis (GSEA) of the nearest genes to enriched accessibility peaks for immune Hallmarks categories.

C, Left - Integrative Genomics Viewer (IGV) tracks of chromatin accessibility for each selected cluster, with MiGl clusters grouped together. scATAC co-accessibility connections are shown. Highlighted with grey boxes are regions with differential accessibility in specific clusters or promoter priming and connections between regulatory regions. Genomic coordinates are shown. Right – Violin plots depicting the expression of selected genes in each individual cluster.

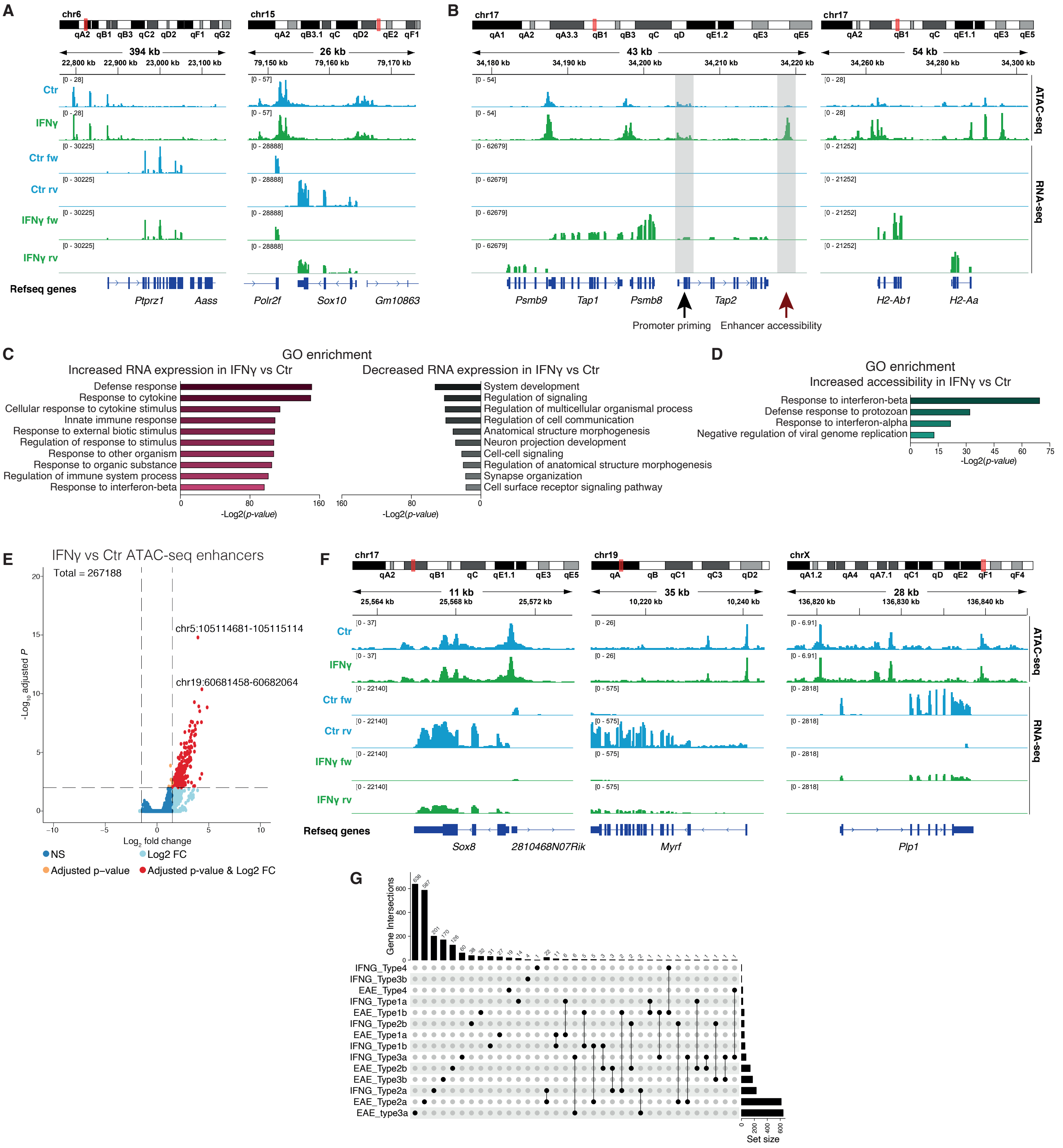

Figure S4. Chromatin landscape upon treatment of mouse primary OPCs with IFNγ, related to Figure 3

A, B, IGV tracks of RNA-seq and ATAC-seq from Ctr-OPCs and OPCs treated with 100 ng/ml IFNγ for 48 hours, centered at loci for marker genes of OPCs (*Ptprz1*) and OLG (*Sox10*) **(a)** and MHC-I pathway and MHC-II genes **(b)**. Highlighted with grey boxes and arrows are regions with differential accessibility in specific conditions or promoter priming, black arrows indicate promoter regions and red arrows indicate putative enhancers. Merged tracks of 3 replicates are shown for ATAC-seq and 4 replicates for RNA-seq. **C,** Top 10 Gene Ontology biological terms for genes upregulated upon IFNγ treatment in OPCs and downregulated upon IFNγ treatment. **D,** Gene Ontology biological terms for genes with increased chromatin accessibility at 500 bp promoter regions upon IFNγ treatment in OPCs. **E,** Volcano plot for chromatin accessibility at annotated enhancers between IFNγ-treated and Ctr-OPCs. Loci with statistical significance are shown in orange and genes with statistical significance and log2 fold change above 1.5 are shown in red. **F,** IGV tracks for genes with decreased expression in IFNγ treatment. **G,** Upset plot with intersection between genes with open chromatin in OPCs in vivo in EAE and in OPCs upon IFNg treatment.

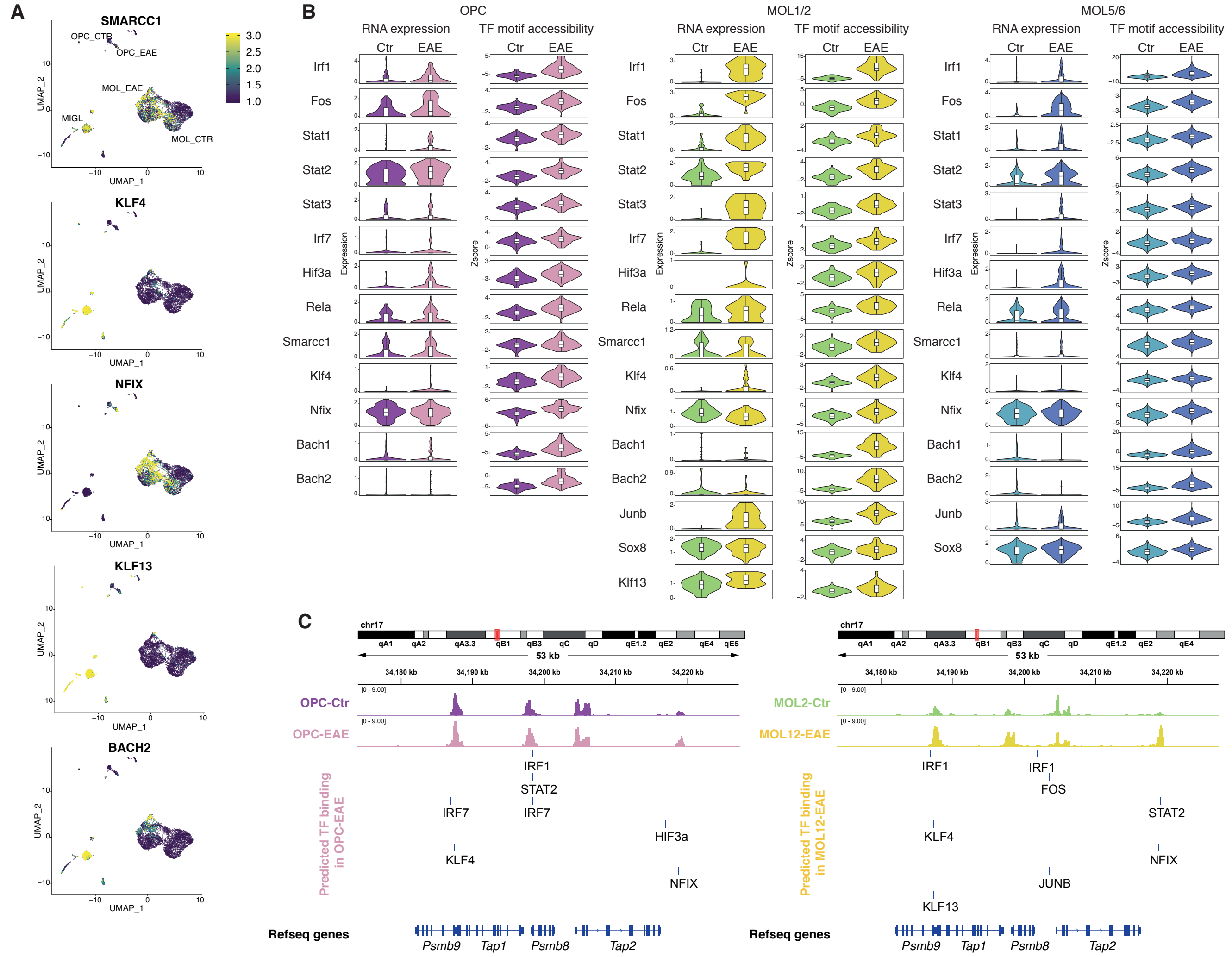

Figure S5. Transcription factor motif variability in oligodendroglia in the EAE mouse model of MS, related to Figure 4.
A, TF motif variability projected on top of UMAP clustering. **B,** Violin plots showing RNA expression values of selected TFs and their TF motif accessibility score in OPCs, MOL1/2 and MOL5/6, comparing cells derived from CFA-Ctr and EAE. **C,** IGV tracks of merged single cell chromatin accessibility of different populations and TF predicted binding sites in OPCs and MOL1/2 cells derived from EAE mice in the *Psmb9*-*Tap2* locus.

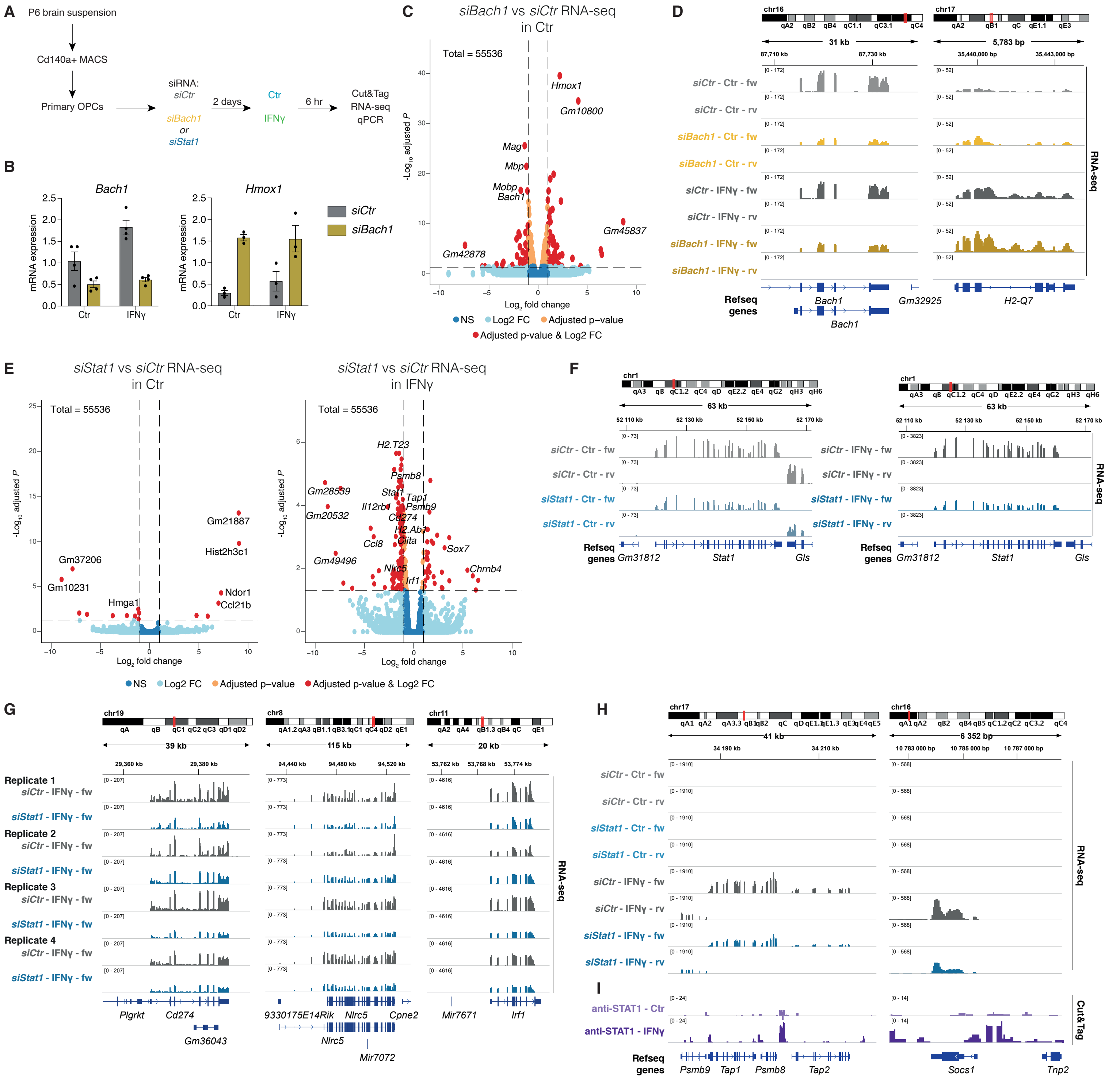

Figure S6. BACH1 and STAT1 transcription factor knock down in primary OPCs upon IFNγ, related to Figure 4

A, Schematic illustration of the experimental set-up of transcription factor knock down experiments. **B**, qRT-PCR targeting selected genes upon transfection of primary OPCs with siRNAs targeting *Bach1*. **C,** Volcano plots showing differential expressed genes in RNA-seq upon transfection of primary OPCs with siRNAs targeting *Bach1*. 4 biological replicates were performed. Genes with adj p-value < 0.05 and log2 fold change above 1.5 are shown in red. **D**, IGV tracks for RNA-seq upon transfection of primary OPCs with siRNAs targeting *Bach1*. **E**, Volcano plots showing differential expressed genes in RNA-seq upon transfection of primary OPCs with siRNAs targeting *Stat1* before treating with IFNγ for 6 hours. 4 biological replicates were performed. Genes with adj p-value < 0.05 and log2 fold change above 1.5 are shown in red. **F**, **G**, **H**, IGV tracks for RNA-seq in IFNγ-treated and Ctr-OPCs after transfection with with siRNAs targeting *Stat1* for selected genes. **I**, IGV tracks showing STAT1 binding in OPCs upon IFNγ treatment and in Ctr-OPCs, assessed with CUT&Tag. 3 biological replicates were performed.

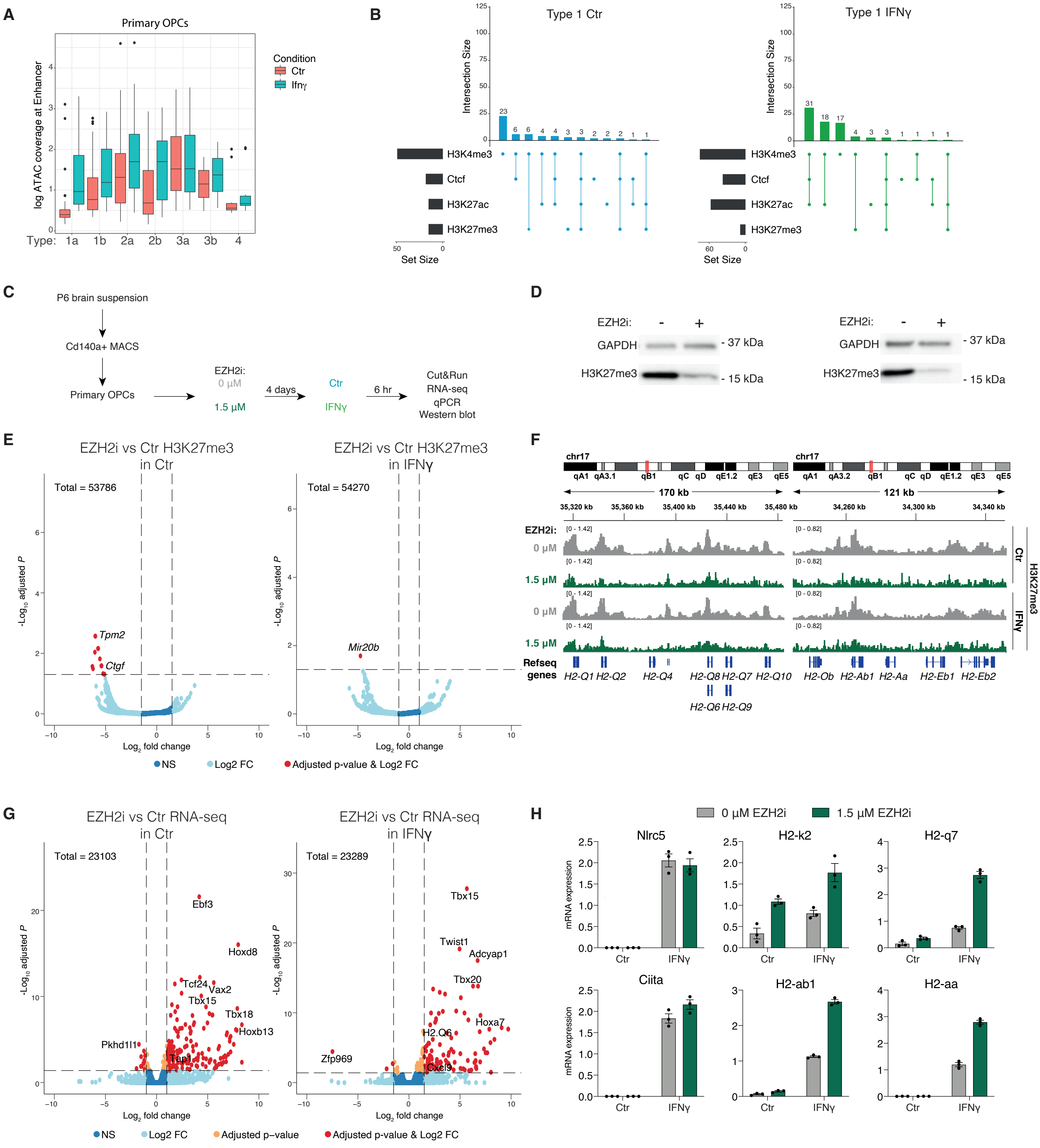

Figure S7, Chromatin accessibility and transcriptional changes upon treatment of mouse primary OPCs with the EZH2 inhibitor EPZ011989, related to Figure 3, 5 and 6.

A, ATAC coverage over enhancers (as predicted by ABC) of Type 1-4 genes (from Figure 3b) in OPCs treated with IFNγ. **B,** Upset plots of Cut&Run Ctr-OPCs and IFNγ treated peaks intersection in Type1 (genes with increased expression and chromatin accessibility with IFNγ). Top barplot shows the number of intersecting peaks per combination, left barplot shows the size of each peak dataset, the matrix shows the Cut&Run peaks sets (dots) and shared (connecting line) in each combination. **C,** Schematic overview of EZH2 inhibitor EPZ011989 (EZH2i) experiments. **D,** Western blot with antibodies against H3K27me3 and GAPDH (control) in EZH2i cells vs. Ctr in IFNγ-spiked OPCs, two replicates are shown. **E,** Volcano plots for differences in H3K27me3 occupancy between EZH2i vs. Ctr, in Ctr-OPCs (left) and OPCs upon IFNγ treatment (right), assessed with Cut&Run. Three replicates are performed. Genes with statistical significance and log2 fold change above 1.5 are shown in red. **F,** IGV tracks for H3K27me3 occupancy upon EZH2i vs. Ctr, in Ctr-OPCs and OPCs upon IFNγ treatment at MHC-I and MHC-II loci. Merged tracks of three replicates are shown. **G,** Volcano plots showing differential gene expression between EZH2i vs. Ctr, in Ctr-OPCs (left) and OPCs upon IFNγ treatment(right). Genes with statistical significance are shown in orange and genes with statistical significance and log2 fold change above 1 are shown in red. Three replicates are performed. **H,** qRT-PCR analysis of MHC-I and MHC-II pathway genes in OPCs upon treatment with 1,5 μM EZH2 inhibitor EPZ011989 (EZH2i) for 4 days, with subsequent co-treatment with 100 ng/ml IFNγ for 6 last hours. Error bars represent SEM, three replicates are shown.

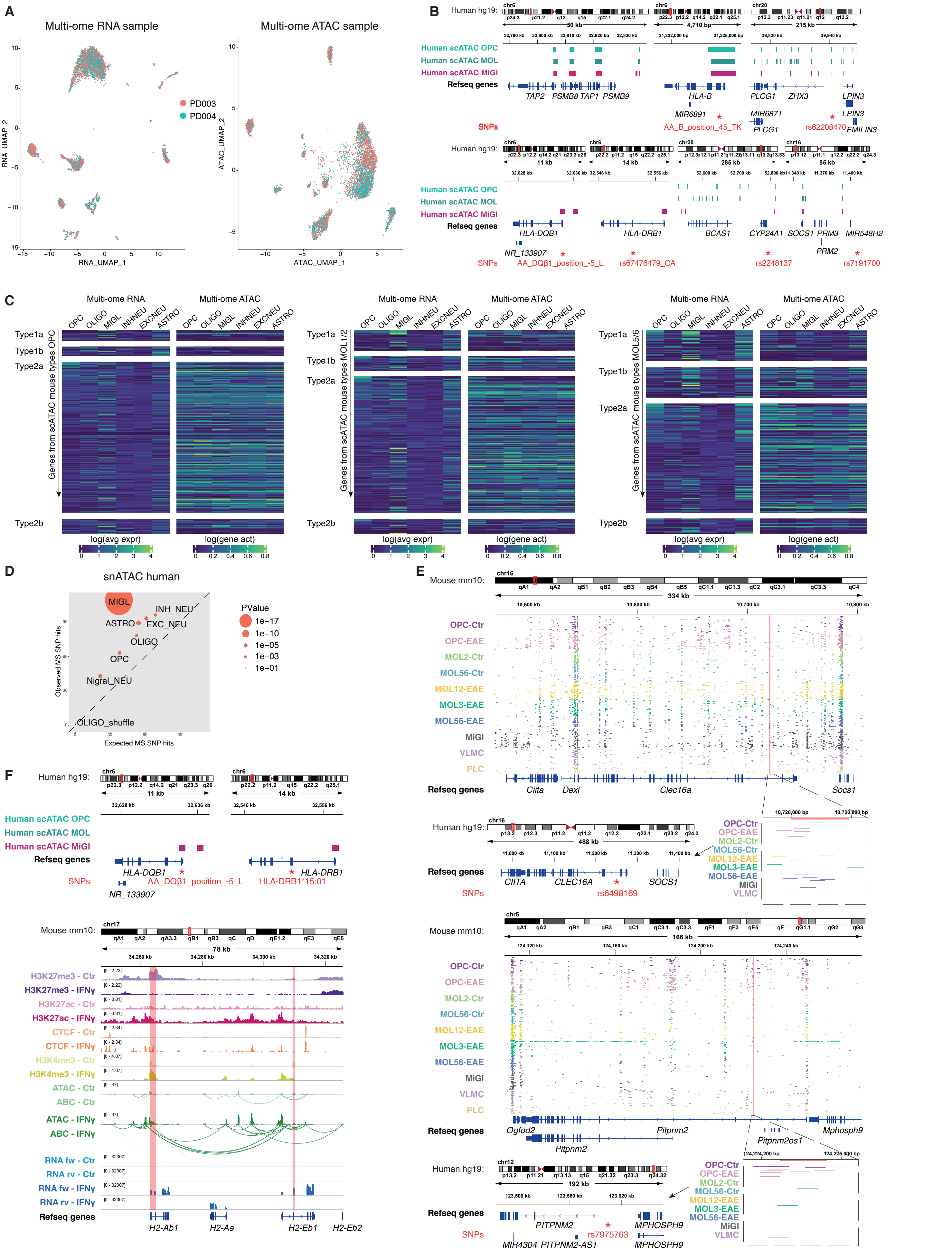

Figure S8, Chromatin accessibility at MS susceptibility loci in human and mouse oligodendroglia, related to Figures 2, 7 and 8. A, UMAP based on chromatin accessibility (right) and transcriptome (left) of 10x Genomics multi-ome of brain grey matter from 2 healthy individuals, by individual **B**, SNP coordinates for MS SNPs in the hg19 human genome reference and scATAC-seq data from human healthy individuals. **C**, Analysis of Type1 and 2 genes from mouse OLG (Fig. 2A) in the human multi-ome dataset. **D**, GREGOR overlap with MS associated GWAS variants. overlapping the human snATAC-seq peaks. Dot size scaled to adjusted p-value and adjusted p-values below 0.01 in red. **E,** SNP coordinates for two MS SNPs in the hg19 human genome reference and chromatin accessibility in scATAC-seq in corresponding locations in the mouse mm10 genome reference. IGV tracks of chromatin accessibility in 50 randomly selected individual cells from scATAC-seq are shown. Red boxes show scATAC-Seq peaks from mouse overlapping with SNP location. **F,** SNP coordinates for two MS SNPs in the hg19 human genome reference with corresponding chromatin accessibility regions derived from merged scATAC-seq populations from the adult brain from human healthy individuals. Corresponding locations in the mouse mm10 genome reference with IGV tracks for bulk ATAC-seq, RNA-seq, ABC model and Cut&Run against H3K27me3, H3K27ac, CTCF, H3K4me3 in IFNγ treated and Ctr-OPCs. Red boxes show chromatin accessibility peaks from mouse overlapping with SNP location and their ABC connections.
